# The Epidemiology and Determinants of Opportunistic Intestinal Parasites Among HIV-Positive Patients Attending Care and Treatment Centers in Northcentral Ethiopia

**DOI:** 10.1155/japr/3857677

**Published:** 2025-06-17

**Authors:** Yitbarek Mulie, Sissay Menkir, Abayeneh Girma

**Affiliations:** ^1^Department of Biology, College of Natural and Computational Science, Mizan-Tepi University, Tepi, Ethiopia; ^2^Department of Biology, College of Science, Bahir Dar University, Bahir Dar, Ethiopia; ^3^Department of Biology, College of Natural and Computational Science, Mekdela Amba University, Tulu Awuliya, Ethiopia

**Keywords:** antiretroviral therapy (ART), epidemiology, Ethiopia, HIV, opportunistic intestinal parasites (OIPs)

## Abstract

**Background:** Opportunistic intestinal parasites (OIPs) cause significant morbidity and mortality among HIV-positive people due to the decline of CD4+ T-cells. In Ethiopia, the burden of this infection is high due to poor personal and environmental hygiene. The present study is aimed at finding the epidemiology and determinants of OIPs in human immunodeficiency virus-infected patients attending antiretroviral therapy (ART) at Debre Tabor General Hospital.

**Methods:** A hospital-based cross-sectional study was conducted among 384 systematically selected patients attending the Debre Tabor General Hospital ART Clinic from December 2019 to February 2020. For parasitological examinations, wet mount, formol–ether sedimentation, and modified Ziehl–Neelsen staining methods were used. CD4 count was reviewed from medical records. Data were entered and analyzed using SPSS Version 23. Logistic regression was utilized to analyze the relationship between factors linked with OIPIs. Variables with *p* < 0.05 were considered to be statistically significant.

**Results:** The overall prevalence of OIPs was 17.9%. The most commonly identified parasites were *Cryptosporidium* species (8.59%), followed by *Cystoisospora belli* (6.77%) and *Cyclospora cayetenensis* (2.60%). Residence (AOR = 0.197; 95% CI = 0.053–0.734), CD4+ count (AOR = 49.08; 95% CI = 9.440–228.777), ART adherence (AOR = 7.427; 95% CI = 2.488–22.172), diarrhea (AOR = 7.063; 95% CI = 1.882–26.512), fingernail trimming (AOR = 3.665; 95% CI = 1.040–12.918), hand washing habit after toilet (AOR = 10.409; 95% CI = 1.398–77.497), and drinking water source (AOR = 14.721; 95% CI = 3.349–64.71) were determinants for OIPs.

**Conclusion:** The study indicated that the coinfection rate of OIPs is high among ART patients. It was also found that urban residence, poor ART adherence, individuals with diarrhea, irregular trimming of the fingernail, a lack of hand washing habits after the toilet, drinking unsafe water, and having a CD4+ count < 200 cells/*μ*L predicted the presence of OIPs.

## 1. Introduction

Opportunistic intestinal parasites (OIPs), such as coccidian parasites (like *Cryptosporidium*, *Cystoisospora belli*, and *Cyclospora cayetanensis*), *Microsporidia* species, and *Blastocystis hominis*, commonly infect individuals with weakened immune systems, especially those with Human Immunodeficiency Virus (HIV) [1, 2]. About 80% of patients with acquired immune deficiency syndrome (AIDS) pass away due to illnesses associated with opportunistic infections rather than the virus itself, such as OIPs, mostly when the level of CD4+ T cells drops below 200 cells/*μ*L [3–5]. Research indicates that HIV/AIDS patients with a CD4+ T cell count below 100/*μ*L often suffer from ongoing diarrhea. This can lead to dehydration, problems with electrolytes, abdominal pain, and losing weight [5–8].

Past research on OIP infections has shown that a range of factors play a role in spreading the infection in regions with tropical and subtropical climates. These include determinants like not keeping sanitary facilities clean, using unsafe water sources, not having enough toilets, not teaching people about how the infection spreads, not following good hygiene habits, not having enough income, and living in overcrowded conditions [2, 9, 10].

Different researchers reported the prevalence of coccidian parasites in Ethiopia among people with HIV [2, 3, 5]. According to the review done in Ethiopia from 2001 to 2019 [11], approximately 11% of people living with HIV/AIDS (PLWHA) were found to have *Cryptosporidium* infection based on a combined prevalence estimate. As per the finding by Hailu et al. [12], the overall epidemiology of *Cryptosporidium* spp. among HIV/AIDS patients from Wurgissa and Hawassa districts was 46.0%. *Cystoisospora belli* infection among HIV/AIDS patients was reported in different parts of Ethiopia at a different times [13–15], with a prevalence of 12%, 15.3%, and 22.5%, respectively. There are no adequate works of literature on the epidemiological information of *Cyclospora cayatenesis*, but a few studies [16–18] reported 2.8%, 3.7%, and 5.9% prevalence among HIV-positive patients, respectively. Generally, the epidemiology of coccidian parasites among people with HIV in the sub-Saharan region is still poorly understood [19].

In several rural villages within the Debre Tabor district, clean water is scarce, forcing community members to rely on unsafe water sources. This reliance on diverse water sources significantly increases the likelihood of illness from waterborne diseases. Additionally, the levels of environmental sanitation and personal hygiene in these rural villages are notably poor (South Gondar Health Office, personal communication, November 2019). Additionally, there is no information about the coinfection rate of OIPs among patients attending ART. Up-to-date information regarding OIP magnitude and predictors is vital for better treatment and interventions and may support national and international HIV END strategies and eradication programs. Therefore, the study is aimed at estimating the prevalence of OIPs and determining drivers among HIV-positive patients on ART treatment in Debre Tabor General Hospital, Northcentral Ethiopia.

## 2. Materials and Methods

### 2.1. Study Design, Period, and Area

A hospital-based cross-sectional study was conducted between December 2019 and February 2020 at Debre Tabor General Hospital in Debre Tabor town, located in Northcentral Ethiopia. Debre Tabor is a town situated 667 km away from Addis Ababa, elevated at 2706 m above sea level. Serving around 2.3 million individuals, the hospital boasts over 110 beds. The hospital has been providing HIV counseling, testing, treatment, and follow-up services since the 1990s. At the hospital's ART clinic, 2210 HIV/AIDS patients were actively receiving ART during the study period. The medical records, however, pointed to a greater figure.

### 2.2. Study Population

At Debre Tabor General Hospital, all HIV-positive individuals who registered at the ART clinic were included as a source of the population. All registered HIV/AIDS patients actively attending appointments or follow-up at the ART clinic of the hospital during the study duration were chosen for the research.

#### 2.2.1. Inclusion Criteria

This study encompassed all individuals with HIV/AIDS who provided written consent to participate and visited ART clinics during the examination period. Only those who expressed a desire to take part in the study were included.

#### 2.2.2. Exclusion Criteria

Those participants who have been taking antiparasitic medication during the last 3 months were excluded from this study. Also, those severely ill patients who were unable to respond to questions or with a history or diagnosis of acute or chronic disease–causing immune suppression (COVID-19, tuberculosis, asthma, cirrhosis, cancer, diabetes, cystic fibrosis, transplant recipients, and chronic obstructive pulmonary disease) were excluded from the study.

### 2.3. Sample Size Determination and Sampling Techniques

We used a formula to determine the size of the sample (*n*) for a single population proportion [20]. In this particular research location, as there has been no prior data available, we identified a prevalence percentage of 50% (*p* = 0.5), inserted a confidence level of 95% (*Z* = 1.96), and allowed for a margin of error of 5% (*d* = 0.05) in order to determine the necessary sample size.

 $$\begin{array}{c} n=\frac{\left(Z^{2}\right)\left(p\right)\left(1-p\right)}{{\left(d\right)}^{2}}=\frac{{\left(1.96\right)}^{2}\left(0.5\right)\left(1-0.5\right)}{{\left(0.05\right)}^{2}}=384, \end{array}$$

where *n* is the sample size, *Z* is the confidence level, *d* is the margin of error, and *p* is the expected prevalence or proportion.

After adding 10% (38) for contingency, the final sample size was 422 individuals. A systematic random sampling method was employed by calculating the *K* value (interval), where *N* = 2210, *n* = 422, and *K* = 2210/422 = 5. The first subject was recruited by the lottery method, so every fifth ART-following patient who came to the ART clinic from December 2019 to February 2020 was included in the sample until the required sample size was reached.

### 2.4. Study Variables

The study was focused on the following independent variables: sociodemographic status, environmental and personal hygiene, the status of ART adherence, clinical conditions, and the immune status of the patient. The prevalence of OIPs was considered the response variable in the present study.

### 2.5. Data Collection Technique

A structured questionnaire was prepared, pretested, and validated before 2 weeks of study at Felege Hiwot Referral Hospital in Bahir Dar on 20 people with HIV to evaluate the quality and strength of the questionnaire. The ART clinician and principal investigators provided a directional view and awareness of the study's objective to encourage active participation in filling out the questionnaire. The questionnaire was originally prepared in English and then translated to Amharic and back to English to obtain content validity. After modifying and assuring its quality, the pretested questionnaires were administered to respondents to generate data on risk factors of OIPs, such as questions related to sociodemographic characteristics, behavioral habits, hygienic and sanitary conditions, environmental conditions, and clinical information.

### 2.6. Data of CD4+ Lymphocyte Counts

The recent CD4+ T cell count data was taken from patients' medical records. Fluorescence-activated cell sorting (FACS) analysis, a specialized form of flow cytometry (Becton Dickinson Immunocytometry Systems, San Jose, California, United States), was used to determine the count at the Debre Tabor General Hospital ART clinic.

### 2.7. Stool Sample Collection and Preservation

The proper method for the collection of stool samples was provided to ensure the favorable condition of the stool sample. Also, an oral description and specific instructions on proper handling and avoiding contamination of the stool specimen were given to the entire participant by the investigator. Disposable plastic cups with an applicator spoon for the collection of stool samples were provided for study subjects by labeling them with their unique codes, and after that, they were told to bring about 5–10 g of fresh stool specimen each. Initially, a wet mount was prepared, followed by the preservation of the remaining sample in a 10% formalin solution. The collected stool samples were promptly preserved in 10% formalin until microscopy, which helps maintain the shape and size of protozoan and helminth parasites and prevents further development [21].

### 2.8. Parasitological Laboratory Examination Procedure

#### 2.8.1. Direct Wet Mount

A direct wet-mount smear was examined immediately by using normal saline. Observe under a light microscope at 100x and 400x magnifications to detect the presence of motile parasite stages. After preserving the remaining sample in 10% formalin, it was carefully examined using the formol–ether concentration technique along with a modified Ziehl–Neelsen staining method [21, 22].

#### 2.8.2. Formol–Ether Concentration Method

Briefly, 1 g of stool was taken from the preserved stool, mixed with a 10% formalin solution, and sieved through double-layered gauze. The filtrate was transferred to a 15-mL conical centrifuge tube and added to 7 mL of formalin saline using a plastic pipette and 3 mL of diethyl ether. Then, the content was shaken vigorously and centrifuged for 5 min at 3000 rpm. After collecting the sediment, a slide was prepared and examined under a light microscope at both 100x and 400x magnification to identify eggs, cysts, and larvae of various parasites.

#### 2.8.3. Modified Ziehl–Neelsen Acid–Fast Stain

A small portion of the fresh stool sample was processed for the detection of *Cryptosporidium*, *Cystoisospora belli*, and *Cyclospora cayetanensis* oocyst. Sediment from stool was spread thinly and left to dry before being fixed with methanol for 5 min. After that, it was stained with carbol fuchsine for a duration of 20 min. After washing the slides in tap water, they were decolorized with 1% hydrochloric acid in methanol for 1–3 min and stained with methylene blue for 1 min. The slides were then washed in tap water and observed under a light microscope with magnification using oil immersion objective lenses [22].

### 2.9. Quality Control

Before actual data collection began, experienced laboratory technologists pretested the questionnaire and other materials, such as the microscope, centrifuge, and chemicals used during data collection. The specimens were also checked for serial number, quality, and collection procedures. The stool samples were examined by two laboratory technicians in order to avoid observer bias. If conflicting results were found, a different technician examined the stool sample slide, and their findings were deemed conclusive. In addition to the color atlas, the laboratory technicians used known positive coccidian parasite samples as positive controls and performed staining. Furthermore, the researchers validated their staining procedure against the literature using known positive samples to ensure it was functioning as expected. Regarding CD4+ T cells, data collectors were trained, and after the data collection process, the collected data were double-checked for completeness by three investigators, and any incomplete or misfiled data were filed again. Next, the findings from the lab were detailed meticulously on a neatly organized document before being affixed to the survey.

### 2.10. Data Analysis

Descriptive statistics were used to understand the property and the pattern of data collected from respondents. The data obtained from questionnaires was entered through the EPI-INFO 7 computer program for precoding data and then exported to SPSS Version 23. A chi-square test was applied to determine the association between parasitic infection and CD4+ T cell count. The logistic regression model was employed to assess potential risk factors. The strength of associations was measured using odds ratios (OR) and 95% confidence intervals (CI). Multivariate regression analysis was then applied for variables with *p* < 0.25 in the bivariate analysis. The association between variables was considered statistically significant in the multivariate analysis only if the *p* value was < 0.05 [23].

### 2.11. Ethics Statement

In general, our research adhered to the guidelines set out in the Declaration of Helsinki [24]. Ethical clearance was obtained from the ethical review committee of Bahir Dar University College of Science (Reference No. PGRCSVD/106/2019, dated November 20, 2019) (Supporting Information 1: File S1). Every participant in the study provided written consent beforehand. If the participant was under 18, their legal guardians also gave written consent. Participants could decline or step back from the study without it impacting their care. This study adheres to established ethical research principles to ensure confidentiality of HIV patient data, and access to this sensitive information is restricted solely to Yitbarek Mulie and Sissay Menkir (Supporting Information 2: File S2). Participants' details were kept confidential using coded study numbers. If a participant tested positive, they were directed to medical help.

## 3. Results

### 3.1. Sociodemographic Characteristics of the Study Participants

A total of 384 HIV-positive individuals were included. Of these, 222 (57.8%) were females, and 215 (56.0) of the respondents were aged 30–49, with the mean (SD) age of 34.30 (± 11.073) years. The majority of the participants were urban residents, 262 (68.2%); married, 241 (62.8%); civil servants, 127 (33.1%); and above secondary educational status, 124 (32.3%) (Table 1).

### 3.2. Hygienic Habits and Clinical Characteristics of the Study Participants

The majority of the participants used tap water for drinking 256 (66.7%) and had an occasional hand washing habit before a meal 349 (90.9%), CD4+ T-cell counts above 500 cells/*μ*L 143 (37.2%), 274 (71.4%) had adherence to ART treatments, 268 (69.8%) had no animal contact, 194 (50.5%) practiced an open waste disposing mechanism, 309 (80.5%) nondiarrheic, 283 (73.7%) trimmed nails regularly, 283 (73.7%) and 220 (57.3%) used unprotected water for washing fruits and vegetables, 310 (80.9%) had used a latrine, 309 (80.5%) used unprotected water for washing purposes, 195 (50.8) had no handwashing habit after toilet, and 239 (62.2%) did not consider the safety of water and food while traveling (Table 2).

### 3.3. Major Intestinal Parasitic Species Identified Among the Examined HIV/AIDS Patients

**Figure 1:**
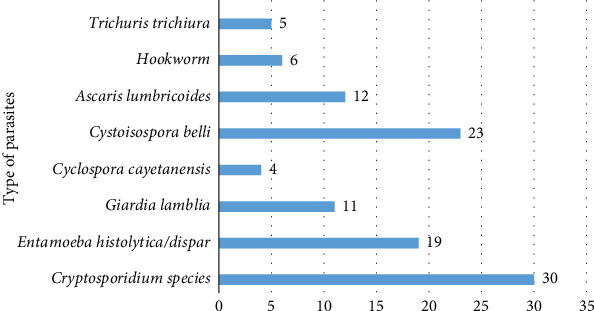
Intestinal parasites were detected in HIV/AIDS patients who were on ART in Debre Tabor General Hospital (DTGH).

Totally, 384 samples of stool were reviewed to detect infections caused by parasites within the intestines. Among these, 122 (31.7%) were found positive for an intestinal parasite (both opportunistic and nonopportunistic parasite species). The overall prevalence of opportunistic parasite infections was found to be 69/384 (17.96%). From 122 positive cases, 110 (28.7%) were infected with single parasites (Figure 1), while 12 (3.125%) had mixed infections with nonopportunistic protozoan and helminth parasites (Table 3).

In this study, eight species of intestinal parasites were detected. Of these parasites, five belonged to protozoans, and the remaining three belonged to helminthic parasites. The most frequent parasite species detected from the opportunistic intestinal protozoan parasites was *Cryptosporidium* species (8.59%), followed by *Cystoisospora belli* (6.77%) and *Cyclospora cayetenensis* (2.60%). Regarding the non-OIPs, *Entamoeba histolytica*/*dispar*, *Ascaris lumbricoides*, *Giardia lamblia*, hookworm, and *Trichuris trichiura* were 4.93%, 3.11%, 2.85%, 1.55%, and 1.3%, respectively (Figure 1).

There were (3.1%) cases of double infections with opportunistic intestinal protozoan–protozoan and protozoan–helminths observed. Among the double infections, all (12) cases displayed coccidian parasites (*Cryptosporidium* species, *Cyclospora cayetanensis*, and *Cystoisospora belli*) had three, six, and three mixed infections, respectively (Table 3).

### 3.4. Major Factors Associated With OIPs

In multivariate logistic regression analysis, patients who lived in rural areas were less likely to acquire OIPs than patients who had lived in urban areas (AOR = 0.197%, 95% CI: 0.053–0.734, *p* = 0.015). However, the rest of the sociodemographic factors were not significantly associated in a multivariate analysis (Table 4).

Patients whose CD4^+^ T-cell count < 200 cells/*μ*L were more likely to be infected by OIPs (AOR = 49.08, 95% CI: 9.440–228.777, *p* < 0.001). Patients who had poor adherence to ART treatment were more likely to be infected with OIPs (AOR = 7.427, 95% CI: 2.488–22.172, *p* < 0.001). Study participants who replied yes for diarrheal history were more likely to be infected with OIPs (AOR = 7.063, 95% CI: 1.882–26.512, *p* = 0.004). Patients who reported irregular trimming of fingernails were more likely to be infected with OIPs (AOR = 3.665, 95% CI: 1.040–12.918, *p* = 0.043). Moreover, patients who drank water from unprotected source and had no handwashing habit after the toilet were 14 and 10 times more likely to be infected with OIPs (AOR = 14.721, 95% CI: 3.349–64.71, *p* < 0.001) and (AOR = 10.409, 95% CI: 1.398–77.497, *p* = 0.022), respectively. However, in multivariate analysis, the majority of the study variables were not significant (Table 5).

### 3.5. Association of OIP With CD4+ T-Cell Counts

The proportion of OIPs was significantly higher in patients with a CD4^+^ count < 200 cells/*μ*L when compared with the other group with a CD4^+^ count > 200. There were 56 individuals with CD4+ counts < 200 cells/*μ*L, of whom 42 had OIP infections. *Cryptosporidium* species (*n* = 30) was the most common pathogen, followed by *Cystoisospora belli* (*n* = 23), *Cyclospora cayetanensis* (*n* = 4), and double infection (coccidian parasite with other non-OIPs) (*n* = 12). Of the 87 patients with a CD4^+^ count of 200–349 cells/*μ*L, OIPs were detected in 21 patients. Fifteen patients had *Cryptosporidium* species, and six had mixed infections (Table 6).

On the other hand, 98 study participants had a CD4^+^ cell count between 250 cells/*μ*L and 499 cells/*μ*L. From those, only four cases were infected: (2) with *Cryptosporidium* species, (1) with *Cyclospora cayetanensis*, and (1) with double parasitic infections. The remaining 143 study participants had a cell count above 500; only two cases were infected, one with *Cryptosporidium* and one with double parasitic infections (Table 6).

## 4. Discussion

Individuals with a weakened immune system are more susceptible to intestinal parasites. People with HIV/AIDS often have intestinal parasite infections. HIV weakens the immune system, making it easier for intestinal parasites to cause malnutrition, weight loss, and ongoing diarrhea, making the patient's health worse [25].

In the current study, the prevalence of OIPs among HIV/AIDS patients who attend ART was 17.96%. It is consistent with studies conducted in Arba Minch (17.2%) [18] and Jimma (15.38%) [26] and outside Ethiopia 16.49% in Cameroon [27]. The result was higher than the reports of 1.96% in Tehran, Iran [28], and 8.9% in Bahir Dar, Ethiopia [5]. However, the prevalence rate was lower than the result reported in Bahir Dar, Ethiopia (69.7%) [14], India (62.5%) [6], and Mexico (69%) [29]. The differences in prevalence rates may be attributed to variations in participants' awareness and understanding of the effective use of antiretroviral therapy (ART), both during treatment and prior to initiation. Increased knowledge about ART could lead to better adherence and management of health, ultimately reducing the incidence of OIPs among HIV/AIDS patients.

*Cryptosporidium* species was the most common OIP detected in HIV/AIDS patients who are on ART (8.5%). Similar results were reported in Arba Minch (8.63%) [18] and India (9.0%) [30]. However, the present prevalence was lower than the previous prevalence of 13.2% in Hawassa [31], 13.3% in Jimma [26], 15.68% in Eastern Tigray [32], 28.6% in Addis Ababa [13], and 33% in India [33]. However, the magnitude of *Cryptosporidium* sp. in HIV seropositive patients was markedly higher than the prevalence reported in the Komblecha (1.4%) [34], Dessie (1.5%) [35], Harar (2.2%) [36], and Gondar (3.1%) [3]. The predominance of *Cryptosporidium* species in the study settings may be attributed to: (1) environmental factors, including the consumption of untreated water from wells, rivers, or poorly maintained pipelines; swimming in contaminated ponds; using contaminated water to wash raw fruits and vegetables; poor hand hygiene (such as not washing hands with soap after defecation or before meals); and open defecation practices; (2) zoonotic factors, such as due to Debre Tabor's agrarian economy means many patients likely have direct contact with infected animals such as cattle, calves, goats, sheep, or their feces (due to the use of animal manure as fertilizer) and exposure to contaminated animal products; and (3) healthcare-related factors, including inadequate handwashing facilities in households and healthcare settings, delayed ART initiation in untreated HIV patients, cross-contamination risks, and the lack of routine stool PCR or antigen testing in DTGH.

The second OIP identified was *Cystoisospora belli,* which accounts for 6.7%. This study is consistent with findings performed in Eastern Tigray [32], South-Western Ethiopia [16], and Wolayita Sodo [17], where they reported 7.1%, 7.4%, and 8.5%, respectively. The result was higher than the reports of 0.71% in Addis Ababa [37], 1.0% in Bahir Dar [5], 2.2% in Harar [36], 3.9% in Jimma [26], and 2.7% in India [33]. Conversely, higher figures than the current study were reported in Butajira (14.4%) [38], Bahir Dar (15.5%) [14], Addis Ababa (22.5%) [13], and India (17.24%) [30]. The low epidemiology of *Cystoisospora belli* may be attributed to the frequent administration of drugs that combat OIPs, such as antihelminthics, as well as differences in the location of the studies [1, 39].

*Cyclospora cayetanensis* was the least OIP identified, resulting in 2.60% infection rates. The prevalence is comparable to a study conducted in Wolayita Sodo (2.8%) [17]. Conversely, this result was lower than the reports from South-Western Ethiopia (3.7%) [18], Jimma (3.9%) [26], and Arba Minch (5.90%) [18]. This finding is higher than research conducted in Hawassa (0.4%) [31], Eastern Tigray (0.42%) [32], and India (1.0%) [30]. This might be due to the fact that PLWHA have weakened immune systems, making them more susceptible to *Cyclospora cayetanensis* infection.

Study participants having a < 200 cells/*μ*L count were 49.08 times more likely to be infected by the OIPs compared to clients with any of the CD4 categories. This finding is in line with recent studies conducted elsewhere [2, 5, 28]. It is possible that opportunistic parasites in HIV/AIDS patients on ART can go away on their own as the immune system strengthens [3, 35, 40].

Individuals who did not have the habit of handwashing after the toilet were 10.4 times more likely to be infected with OIPs than their counterparts. Eshetu and colleagues [3] also reported that the prevalence of OIPs is associated with poor handwashing after defecation or before meals. This may be due to the contamination of hands with feces infected by parasites during defecation, so unwashed hands contain a pathogenic parasite that causes infections. Especially, *Cryptosporidium* species is 10–30 oocytes enough to be pathogenic. Handwashing can prevent about 30% of diarrhea-related illnesses.

The risk of acquiring OIP infection was 7.03 (95% CI: 1.882–26.512, *p* < 0.004) times higher among HIV-positive individuals with diarrhea as compared to their counterparts. This is in line with the previous studies conducted in Gondar [41], Harar [39], and outside Ethiopia from Nigeria [42] and France [43]. The reason for this is probably that these patients had low CD4+ T-cell counts. Noncompliance with the treatment plan could be another reason for ART not working well. To see positive results, sticking to the treatment plan is crucial. Unfortunately, many people in low-income countries struggle to follow the plan as needed.

In this finding, poor adherence to ART drugs was 7.42 times more likely to be infected by OIPs when compared to good adherence (AOR = 7.427, 95% CI: 2.488–22.172, *p* < 0.001). This study is consistent with the studies done in Bahir Dar [2] and Arba Minch [44]. This is because poor adherence to ART can lead to a decrease in CD4 levels, which weakens the immune system and increases the risk of OIPs [45].

In this finding, patients residing in rural areas were less likely to be at risk for OIP when compared with urban residents. The findings of the current study support the previous research conducted in Burkina Faso [46]. The tendency of people to cluster around cities (population density) causes a lack of access to clean water and increases anthropogenic factors that increase the pollution of water in urban areas. Few participants originate from rural regions. This is reflected in the lower occurrence of parasitism in these areas compared to the higher occurrence in urban areas.

In the current study, HIV/AIDS patients who had drinking water from unprotected sources were 14.721 times more likely to harbor OIPs than those using tap water. The outcomes are supported by a study done in Bahir Dar [5], Dessie [35], Gondar [41], and outside Ethiopia from Nigeria [47]. The present result is also consistent with a previous study conducted in Malaysia [48], which showed that those community residences using river water/untreated water were almost twice as likely to have had an OIP infection as those using tap or treated water, the risk of acquiring OIP infection (OR = 2.08; 95% CI = 1.36–3.21). This study's elevated OR, compared to previous Malaysian studies, may be attributed to the focus on immune-suppressed HIV/AIDS patients, unlike the broader demographic studied in Malaysia.

### 4.1. Limitations of the Study

Some OIPs were not detected because of the nonavailability of reagents and the nonapplicability of all techniques for *Microsporidia* and molecular methods to distinguish the species of parasites. Hence, the prevalence of OIPs among the study participants might have been underestimated.

## 5. Conclusion

OIPs affected around 17.96% of ART patients, with *Cryptosporidium* sp. being the most common species identified. This study also found that urban residence, unprotected sources of drinking water, absence of hand washing habit after toilet use, CD4+ T-cell count < 200 cells/*μ*L, individuals with diarrhea, poor ART adherence, and irregular trimming of fingernails were significantly associated with OIPs. Promoting access to clean water sources, health education and behavior changes, enhancing sanitation facilities, routine screening and treatment, and improving adherence to ART could contribute to reducing the burden of OIPs among HIV/AIDS patients in the study area.

## Figures and Tables

**Table 1:** Sociodemographic characteristics of patients with HIV/AIDS taking ART at Debre Tabor General Hospital (DTGH).

**Variables**	**Category**	**Frequency (%)**
Sex	Male	162 (42.2)
	Female	222 (57.8)

Age (year)	Below 18	32 (8.3)
	18–29	95 (24.7)
	30–49	215 (56.0)
	Above 50	42 (11.0)

Residence	Urban	262 (68.2)
	Rural	122 (31.8)

Marital status	Single	35 (9.1)
	Married	241 (62.8)
	Divorced	108 (28.1)

Occupation	Student	45 (11.7)
	Laborer	58 (15.1)
	Merchant	37 (9.6)
	Nonemployed	48 (12.5)
	Farmer	68 (17.8)
	Civil servant	128 (33.3)

Education level	No regular	71 (18.5)
	Primary	100 (26.0)
	Secondary	89 (23.2)
	Above secondary	124 (32.3)

*Note: n* (%) = number of individuals (percentage).

**Table 2:** Hygienic habits and clinical characteristics of patients with HIV/AIDS taking ART at Debre Tabor General Hospital (DTGH).

**Variables**	**Category**	**Frequency (%)**
Handwashing habit before the meal	Sometimes	349 (90.9)
	Always	35 (9.1)

CD4 count in cells/*μ*L	Below 200	56 (14.6)
	200–349	87 (22.7)
	350–499	98 (25.5)
	Above 500	143 (37.2)

Adherence to ART	Yes	274 (71.4)
	No	110 (28.6)

Diarrhea conditions	Yes	75 (19.5)
	No	309 (80.5)

Waste disposing mechanisms	Open defecation	194 (50.5)
	Closed defecation	190 (49.5)

Trimming fingernails	Always	283 (73.7)
	Sometimes	101 (26.3)

Animal contact	Yes	116 (30.2)
	No	268 (69.8)

Source of water for washing vegetables	Un protected	220 (57.3)
	Protected	164 (42.7)

Source of drinking water	Tap water	256 (66.7)
	Unprotected/open	128 (33.3)

Source of water for washing fruits	No wash	84 (21.9)
	Unprotected	201 (52.3)
	Protected	99 (25.8)

Use latrine	Yes	310 (80.9)
	No	74 (19.1)

Type of washing water	Unprotected	309 (80.5)
	Tap water	75 (19.5)

Handwashing habit after toilet	Always	74 (19.3)
	Sometimes	115 (29.9)
	No	195 (50.8)

Considering the safety of water and food when travel	Yes	145 (37.8)
	No	239 (62.2)

*Note: n* (%) = number of individuals (percentage). Protected water source = safeguarded from contamination and pollution (tap water and bottled water). Unprotected water source = vulnerable to contamination (stream, river, spring water well, rain water, and other sources).

Abbreviation: ART = antiretroviral therapy.

**Table 3:** The prevalence of mixed infection of coccidian parasites with nonopportunistic intestinal parasites among HIV/AIDS patients in DTGH.

**Detected opportunistic parasite (no. of patients)**	**No of patients with mixed infection ** **n** ** (*%*)**	**Coinfected with (** **n** **)**
*Cryptosporidium* species (33)	3 (0.78)	*Ascaris lumbricoide*s (1) *Giardia lamblia* (2)

*Cyclospora cayetanensis* (10)	6 (1.56)	*Ascaris lumbricoides* (1) *Giardia lamblia* (3) Hookworm (2)

*Cystoisospora belli* (26)	3 (0.78)	*Entamoeba histolytica/dispar* (2) *Ascaris lumbricoides* (1)

Total (57)	12 (3.125)	12

*Note: n* (%) = number of parasites (percentage).

**Table 4:** Bivariate and multivariate logistic regression analysis of opportunistic intestinal parasitic infection with sociodemographic factors among ART attending patients in DTGH, from December 2019 to February 2020 (*N* = 384).

**Risk factors**	**Category**	**Number of examined cases (%)**	**Rate of OIP number (%)**	**COR (95*%* CI)**	**p** ** value**	**AOR (95*%* CI)**	**p** ** value**
Sex	Male	162 (42.2)	35 (21.6)	1.52 (0.903–2.57)	0.114	8.60 (0.34–2.115)	0.742
	Female	222 (57.8)	34 (15.3)	1			

Age	Below 18	32 (8.3)	5 (15.6)	0.93 (0.265–3.24)	0.904		
	18–29	95 (24.7)	14 (14.7)	0.86 (0.321–2.33)	0.773		
	30–49	215 (56.0)	43 (20.0)	1.25 (0.520–3.006)	0.618		
	Above 50	42 (11.0)	7 (16.7)	1			

Marital status	Single	35 (9.1)	11 (31.4)	2.45 (1.016–5.92)	0.46		
	Married	241 (62.8)	41 (17.0)	1.09 (0.59–2.03)	0.768		
	Divorced/widowed	108 (28.1)	17 (15.9)	1			

Occupation	Civil servant	128 (33.3)	9 (7.1)	1			
	Student	45 (11.7)	7 (15.6)	2.44 (0.85–6.98)	0.098	2.789 (0.53–14.535)	0.223
	Laborer	58 (15.1)	21 (36.2)	7.5 (3.16–17.8)	0.023	4.615 (0.810–26.289)	0.085
	Merchant	37 (9.6)	3 (8.1)	1.17 (0.30–4.55)	0.82	0.556 (0.063–4.880)	
	Non employed	48 (12.5)	0 (0.0)	0.000 (0.000)	0.997		
	Farmer	68 (17.8)	29 (42.6)	9.8 (4.28–22.56)	0.018⁣^∗^	3.143 (0.481–20.531)	0.232

Education level	No regular education	71 (18.5)	26 (36.6)	0.58 (0.264–1.011)	0.002⁣^∗^	0.915 (0.235–3.557)	0.898
	Primary	100 (26.0)	23 (23)	0.296 (0.138–0.634)	0.004⁣^∗^	0.783 (0.186–3.300)	0.739
	Secondary	89 (23.2)	13 (14.6)	0.104 (0.042–0.255)	0.013⁣^∗^	0.850 (0.154–4.691)	0.852
	Above secondary	124 (32.3)	7 (5.6)	1			

Residence	Urban	262 (68.2)	36 (13.7)	1			
	Rural	122 (31.8)	33 (27.0)	2.33 (1.36–3.96)	0.002⁣^∗^	0.197 (0.053–0.734)	0.015⁣^∗^

*Note:* Adjusted ratio only computed for *p* < 0.25 in the bivariate analysis and *p* < 0.05 for multivariate analysis. 1 = reference value.

Abbreviation: AOR: adjusted odd ratios, CI: confidence interval, COR: crude odd ratio, OIP: opportunistic intestinal parasite.

⁣^∗^Significant association.

**Table 5:** Bivariate and multivariate logistic regression analysis of opportunistic intestinal parasitic infection with hygienic and clinical information among ART-attending patients in DTGH from December 2019 to February 2020 (*N* = 384).

**Risk factors**	**Category**	**Number of examined cases (%)**	**Rate of OIP number (%)**	**COR (95% CI)**	**p** ** value**	**AOR (95% CI)**	**p** ** value**
CD4+ T-cell count in cells/*μ*L	Below 200	56 (14.6)	40 (71.40	86.87 (27.48–274.56)	< 0.001⁣^∗^	49.08 (9.440–228.777)	< 0.001⁣^∗^
	200–349	87 (22.7)	17 (19.5)	8.43 (2.736–26.03)	0.012⁣^∗^	2.678 (0.513–13.993)	0.243
	350–499	98 (25.5)	8 (8.2)	3.08 (0.90–10.559)	0.072⁣^∗^	3.107 (0.649–14.867)	0.156
	Above 500	143 (37.2)	4 (2.8)	1			

Perfect ART adherences	Yes	274 (71.4)	15 (5.5)				
	No	110 (28.6)	54 (49.1)	16.65 (8.77–31.60)	< 0.001⁣^∗^	7.427 (2.488–22.172)	< 0.001⁣^∗^

ART started in months ago	Below 24 months	290 (75.5)	48 (16.6)	1.311 (0.661–2.59)	0.438		
	25–59 month	31 (8.1)	8 (25.8)	1.75 (0.74–4.15)	0.202		
	Before 60 months ago	63 (16.4)	13 (20.6)	1			

Diarrhea condition	Yes	75 (19.5)	32 (42.7)	5.47 (3.08–9.69)	< 0.001⁣^∗^	7.03 (1.882–26.512)	0.004⁣^∗^
	No	309 (80.5)	37 (12.0)	1			

Waste disposing mechanism	Open defecation	194 (50.5)	54 (27.8)	4.50 (2.436–8.31)	0.018⁣^∗^	0.37 (0.106–1.332)	0.130
	Closed defecation	190 (49.5)	15 (7.9)	1			

Trimming fingernails	Always	283 (73.7)	27 (9.5)	1			
	Sometimes	101 (26.3)	42 (41.6)	6.750 (3.855–11.89)	< 0.001⁣^∗^	3.665 (1.040–12.918)	0.043⁣^∗^

Animal contact	Yes	116 (30.2)	45 (38.8)	6.444 (3.675–11.298)	0.012⁣^∗^	1.104 (0.324–3.759)	0.875
	No	268 (69.8)	24 (9.0)	1			

Source of water for washing vegetable	Unprotected	220 (57.3)	58 (26.4)	4.98 (2.52–9.84)	0.002⁣^∗^	2.10 (0.610–7.24)	0.240
	Protected	164 (42.7)	11 (6.7)	1			

Source of drinking water	Tap	256 (66.7)	17 (6.6)	1			
	Unprotected/open surface water	128 (33.3)	52 (40.6)	9.619 (5.251–17.622)	< 0.001⁣^∗^	14.721 (33.49–64.71)	< 0.001⁣^∗^

Source of water for washing fruits	No wash	84 (21.9)	34 (40.5)	16.10 (5.424–48.00)	0.002⁣^∗^	0.792 (0.107–5.880)	0.819
	Unprotected	201 (52.3)	31 (15.4)	4.33 (1.484–12.64)	0.017⁣^∗^	0.994 (0.189–5.221)	0.994
	Protected	99 (25.8)	4 (4.0)	1			

Use latrine	Yes	310 (80.9)	44 (14.2)	1			
	No	74 (19.1)	25 (34.2)	3.15 (1.764–5.62)	0.013⁣^∗^	1.068 (0.278–4.111)	0.924

Type of washing water	Unprotected	309 (80.5)	66 (21.4)	6.519 (1.99–21.35)	0.012⁣^∗^	1.620 (0.190–13.826)	0.659
	Protected	75 (19.5)	3 (4.0)	1			

Handwashing habit before meal	Always	349 (90.9)	56 (16.0)	1			
	Sometimes	35 (9.1)	13 (37.1)	3.092 (1.471–6.45)	0.03⁣^∗^	1.327 (0.294–5.987)	0.713

Handwashing habit after toilet	No	195 (50.8)	54 (27.7)	9.064 (2.738–30.005)	< 0.001⁣^∗^	10.409 (1.398–77.497)	0.022⁣^∗^
	Sometimes	115 (29.9)	12 (10.7)	2.76 (0.75–10.13)	0.126	3.503 (0.409–29.996)	0.253
	Always	74 (19.3)	3 (4.1)	1			

Consider safety of water and food when you travel	Yes	236 (61.5)	59 (25.0)	4.60 (2.27–9.32)	0.052⁣^∗^	1.693 (0.525–5.464)	0.378
	No	148 (38.5)	10 (6.8)	1			

Eating food by sharing	Yes	145 (37.7)	54 (22.6)	1			
	No	239 (62.3)	15 (10.3)	0.395 (0.214–0.731)	0.06⁣^∗^	0.848 (0.259–2.771)	0.785

*Note:* Adjusted ratio only computed for *p* < 0.25 in the bivariate analysis and *p* < 0.05 for multivariate analysis. Prefect adherence = consistent and complete following of an antiretroviral therapy regimen by a patient, while poor adherence to ART refers to individuals not following their prescribed HIV treatment regimen consistently or correctly. 1 = reference value.

Abbreviation: AOR: adjusted odd ratio, CI: confidence interval, COR: crude odd ratio, OIP: opportunistic intestinal parasite.

⁣^∗^Significance association.

**Table 6:** The association of each coccidian parasite with the CD4^+^ T-cell count status of HIV-infected patients who were on ART in DTGH.

**Detected parasite**	**Frequency of parasite number (%)**	**Count CD4+ cells/*μ*L**				**Chi-square**	**p** ** value**
		**< 200** **N** = 56	**200–349** **N** = 87	**350–499** **N** = 98	**> 500** **N** = 143		
		**No.pos (%)**	**No.pos (%)**	**No.pos (%)**	**No.pos (%)**		
*Cryptosporidium* species	30 (43.4)	12 (21.4)	15 (17.24)	2 (2.04)	1 (0.69)	39.374	< 0.001⁣^∗^
*Cyclospora cayetanensis*	4 (5.79)	3 (5.3)	—	1 (1.02)	—	12.539	0.006⁣^∗^
*Cystoisospora belli*	23 (33.33)	23 (41)	—	—	—	143.29	< 0.001⁣^∗^
Double-parasitic infections	12 (17.48)	4 (7)	6 (6.89)	1 (1.02)	1 (0.69)	11.287	0.010⁣^∗^
Total	69 (100)	42 (70.49)	21 (24.13)	4 (4.08)	2 (1.3)	137.448	< 0.001⁣^∗^

*Note:* No.pos (%) = number of positive. *N* = total number of participant.

⁣^∗^Statistically significant at *p* < 0.05.

## Data Availability

The data that support the findings of this study are available from the corresponding author upon reasonable request.

## Supporting Information

Supporting Information 1 File S1. Ethics approval sheet.

Supporting Information 2 File S2. Yitbarek Mulie's full thesis under the guidance of Sissay Menkir.
