# Supplementary figures and images for: The Epidemiology and Determinants of Opportunistic Intestinal Parasites Among HIV-Positive Patients Attending Care and Treatment Centers in Northcentral Ethiopia
Source: J Parasitol Res. 2025 Jun 17;2025:3857677. doi: 10.1155/japr/3857677 (PMC12187436; doi:10.1155/japr/3857677)

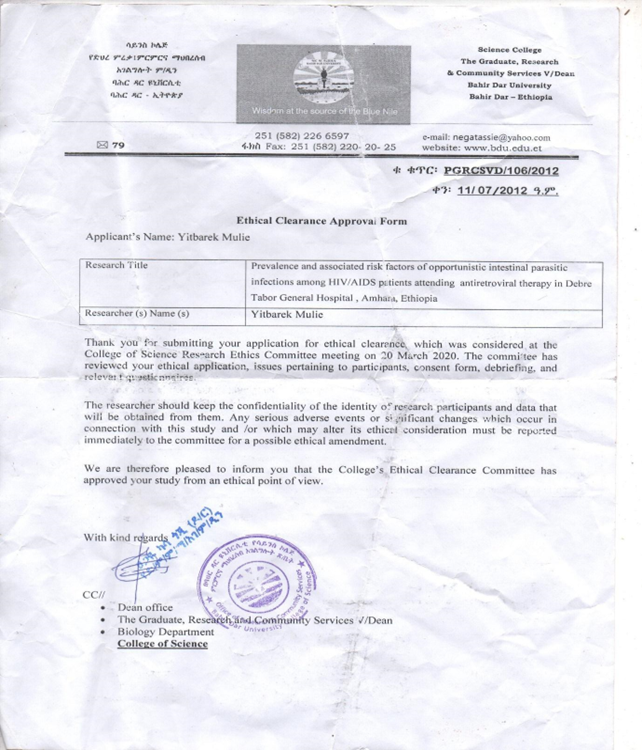

Supplement: Supporting Information 1 — File S1. Ethics approval sheet. [file 3857677.f1.png]
