# Supplementary material for: The Epidemiology and Determinants of Opportunistic Intestinal Parasites Among HIV-Positive Patients Attending Care and Treatment Centers in Northcentral Ethiopia
Source: J Parasitol Res. 2025 Jun 17;2025:3857677. doi: 10.1155/japr/3857677 (PMC12187436; doi:10.1155/japr/3857677)
Supplement: Supporting Information 2 — File S2. Yitbarek Mulie's full thesis under the guidance of Sissay Menkir. [file 3857677.f2.pdf]

DSpace Institution

DSpace Repository **http://dspace.org**

Biology Thesis and Dissertations

2021-03-03

PREVALENCE AND ASSOCIATED RISK FACTORS OF OPPORTUNISTIC INTESTINAL PARASITIC INFECTIONS AMONG HIV/AIDS PATIENTS ATTENDING ANTIRETROVIRAL THERAPY AT DEBRE TABOR GENERAL HOSPITAL, NORTH-WEST ETHIOPIA

YITBAREK, MULIE

http://ir.bdu.edu.et/handle/123456789/12022

Downloaded from DSpace Repository, DSpace Institution's institutional repository

BAHIR DAR UNIVERSITY

COLLEGE OF SCIENCE

DEPARTMENT OF BIOLOGY

PREVALENCE AND ASSOCIATED RISK FACTORS OF OPPORTUNISTIC INTESTINAL PARASITIC INFECTIONS AMONG HIV/AIDS PATIENTS ATTENDING ANTIRETROVIRAL THERAPY AT DEBRE TABOR GENERAL HOSPITAL, NORTH-WEST ETHIOPIA

BY

YITBAREK MULIE

JULY, 2020 E.C

BAHIR DAR UNIVERSITY

SCIENCE COLLEGE

BIOLOGY DEPARTMENT

PREVALENCE AND ASSOCIATED RISK FACTORS OF OPPORTUNISTIC INTESTINAL PARASITIC INFECTIONS AMONG HIV/AIDS PATIENTS ATTENDING ANTI-RETROVIRAL THERAPY AT DEBRE TABOR GENERAL HOSPITAL, AMHARA, ETHIOPIA

A THESIS SUBMITTED TO THE DEPARTMENT OF BIOLOGY, BAHIR DAR UNIVERSITY IN PARTIAL FULFILLMENT OF THE REQUIREMENTS FOR THE AWARD OF MASTER OF SCIENCE DEGREE IN BIOLOGY (BIOMEDICAL SCIENCES)

BY

YITBAREK MULIE

PRINCIPAL ADVISOR'S NAME

SISSAY MENKIR (ASSOCIATE PROFESSOR)

JULY, 2020 E.C

BHIR DAR

ADVISOR’S APPROVAL FORM

BAHIR DAR UNIVERSITY

SCIENCE COLLEGE

BIOLOGY DEPARTEMENT

Approval of Dissertation/thesis for defense

I hereby certify that I have supervised, read, and evaluated this thesis entitled “prevalence and associated risk factors of opportunistic intestinal parasitic infections among HIV/AIDS patients attending antiretroviral therapy at Debre Tabor General Hospital, North-West Ethiopia” by Yitbarek Mulie prepared under my guidance. I recommend the thesis/dissertation be submitted for oral defense.

Advisor’s name ____________________ Signature __________ Date___/____/_____

EXAMINER'S APPROVAL FORM

BAHIR DAR UNIVERSITY

SCIENCE COLLEGE

BIOLOGY DEPARTEMENT

Approval of Dissertation/thesis for defense result

We hereby certify that we have examined this thesis entitled "prevalence and associated risk factors of opportunistic intestinal parasitic infections among HIV/AIDS patients attending antiretroviral therapy at Debre Tabor General Hospital, North-West Ethiopia" by Yitbarek Mulie We recommend that the thesis is is approved for the degree of "Biomedical Science"

Board of Examiners

______________________	____________________	________________
External examiner's name	signature	Date

______________________	______________	__________________
Internal examiner's name	Signature	Date

______________________	______________	_________________
Chair person's name	Signature	Date

DECLARATION

I am a student of the biomedical Sciences of Bahir Dar University, and I am aware of my responsibility of the penal law, I declare and certify with my signature that my thesis entitled "Prevalence and Associated Risk Factors of Opportunistic Intestinal Parasitic Infections among HIV/AIDS Patients Attending Antiretroviral Therapy at Debre Tabor General Hospital, Amhara, Ethiopia" is entirely the result of my work. I have accurately and faithfully cited all my sources, including books, journals, handouts, and unpublished manuscripts, as well as any other media, such as the internet and significant personal communication.

I understand that

Literal citing without using quotation marks and marking the references
Citing the contents of work without marking the references and using the thoughts of somebody else whose work was published, as of our thoughts are counted as plagiarism.

I declare that I understood the concept of plagiarism and I acknowledge that my thesis will be rejected in case of plagiarism.

____

Date

……………………………………….

Signature of thesis writer

ACKNOWLEDGEMENTS

I would like to forward my deepest thankfulness to my advisor Dr. Sissay Minker for his insightful and unreserved professional guidance, reading the manuscript, and forwarding constructive comments throughout my thesis work.

Secondly, I would like to give credit for the role of study participants without their cooperative response this research might not be evident.

Besides, I would like to forward my cordial gratitude to Bahir Dar University for allowing me to study Biomedical Science and to conduct my MSc thesis.

Finally, it would have been impossible to prepare this thesis without the generous help of my families, especially my brother Eyaya Mulie played a significant role in this research work directly or indirectly by motivating, supporting, and encouraging me through ideas and finance.

TABLE OF CONTENTS

Contents Page

DECLARATION .... **iii**

ACKNOWLEDGEMENTS .... **iv**

TABLE OF CONTENTS .... **v**

LIST OF FIGURES .... **ix**

LIST OF TABLES .... **x**

LIST OF ABBREVIATIONS AND ACRONYMS .... **xi**

LIST OF APPENDICES .... **xii**

ABSTRACT .... **xiii**

1 INTRODUCTION **1**

1.1 Background 1

1.2 Statement of the Problem 3

1.3 Objectives of the study 4

1.3.1 General Objective 4

1.3.2 Specific Objectives 4

1.4 Significance of the Study 5

1.5 Limitation of the study 5

2 LITERATURE REVIEW **6**

2.1 Opportunistic Intestinal Parasites in HIV/AIDS Infected Individual 6

2.2 Reason of Intestinal Protozoa more commonly Seen in Immuno-compromised Hosts 7

2.3 Opportunistic parasitic infections in patients with human immunodeficiency virus/acquired immunodeficiency syndrome 7

2.4 Risk Factors for Opportunistic intestinal parasitic infections 9

2.5 Clinically Relevant opportunistic Intestinal Parasitic Infections 10

2.5.1 Cryptosporidiosis 11

2.5.1.1 Morphologic Description 11

2.5.1.2 Epidemiology 12

2.5.1.3 Life Cycle and Transmission 14

2.5.1.4 Pathogenesis 16

2.5.1.5 Clinical Manifestations 17
2.5.1.6 Diagnosis 17
2.5.1.7 Prevention Exposure 18
2.5.1.8 Prevention of Disease 19
2.5.1.9 Treating Disease 20
2.5.1.10 Prevention Recurrence 21
2.5.2 Cyclosporiasis 21
2.5.2.1 Morphologic Descriptions 21
2.5.2.2 Epidemiology 21
2.5.2.3 Life Cycle and Transmission 23
2.5.2.4 Clinical Symptoms and Pathogenesis 24
2.5.2.5 Diagnosis 25
2.5.2.6 Prevention 26
2.5.2.7 Treatments 26
2.5.3 Cystoisosporiasis (Formerly Isosporiasis) 26
2.5.3.1 Morphologic Description 27
2.5.3.2 Epidemiology 27
2.5.3.3 Life Cycle 28
2.5.3.4 Pathogenesis 29
2.5.3.5 Clinical Manifestations 30
2.5.3.6 Diagnosis 30
2.5.3.7 Preventing Exposure 31
2.5.3.8 Preventing Disease 31
2.5.3.9 Treating Disease 31
2.5.4 Blastocytosis 32
2.5.4.1 Epidemiology 32
2.5.4.2 Morphology: 33
2.5.4.3 Life Cycle and transmissions 33
2.5.4.4 Pathogenesis and Clinical Manifestations 35
2.5.4.5 Diagnosis of Blastocytis 35
2.5.4.6 Prevention 36
2.5.4.7 Treatment 36
2.5.5 Microsporidiosis 36
2.5.5.1 Epidemiology 37
2.5.5.2 Pathogenesis 38
2.5.5.3 Life cycle and transmission 38
2.5.5.4 Diagnosis 39
2.5.5.5 Preventing exposure 40
2.5.5.6 Preventing Disease 40
2.5.5.7 Treating Disease 41
2.6 Prevalence of Opportunistic Intestinal Parasites among HIV Seropositive Individuals in Different Parts of Ethiopia 41

2.7 Conceptual framework of associated risk factors with OIPI among HIV/AIDS patients 42

3 MATERIALS AND METHODS 43

3.1 Study Area 43
3.1.1 Operational Definitions...44

3.2 Study Design and Period...44

3.3 Study Population..45
3.3.1 Inclusion Criteria 45
3.3.2 Exclusion Criteria 45

3.4 Sample Size Determination and Sampling Techniques 45

3.5 Study Variables..45

3.6 Data Collection Techniques..46
3.6.1 Questionnaire survey 46

3.7 Data of $CD4^+$ Lymphocyte Counts 46

3.8 Stool Sample Collection and examinations 46
3.8.1 Stool Sample Collection and examination's 47
3.8.2 Parasitological Laboratory Examination Procedure 47
3.8.2.1 Direct wet mount...47
3.8.2.2 Formol-ether concentration method...47
3.8.2.3 Modified Ziehl Neelsen acid-fast stain...47

3.9 Data Quality Control...48

3.10 Data Analysis 48

3.11 Ethical Considerations 49

4 RESULTS 50

4.1 Socio-Demographic Characteristics of the Study Participants 50

4.2 Major Intestinal Parasitic Species Identified among the Examined HIV/AIDS Patients 52

4.3 Prevalence of Opportunistic Intestinal Parasites by Age and Sex 53

4.4 Major factors associated with opportunistic Intestinal parasites 55

4.5 Association of OIP with CD4+ T-Cell Counts 61

5 DISCUSSION 62

6 CONCLUSION 66

7 RECOMMENDATIONS...67

8 REFERENCES **68**

9 APPENDICES **81**

Appendix A: The prevalence of opportunistic intestinal protozoan parasites in Ethiopia. .81

Appendix B: Questionnaire to be completed by Study Participants (English version) 94

Appendix C: Written Consent Form (English version) 96

Appendix D: Questionnaire (Amharic version) 97

Appendix E: Consent Form (Amharic Version) 98

Appendix F: Ethical clearance 100

LIST OF FIGURES

Figures Pages

Figure 1: The Life cycle of *Cryptosporidium* species: Sour..15

Figure 2 Oocyst of *Cryptosporidium* in human faces..18

Figure 3: The Life cycle of *Cyclospora cayetanensis* 24

Figure 4 *Cyclospora* oocysts...26

Figure 5: The Life cycle of *Cystoisospora (Isospora) belli:* 29

Figure 6. Oocyst of *Cystoisospora belli* 31

Figure 7: The Life cycle of *Blastocystis* species...34

Figure 8: The Life cycle of *Microsporidia* species Source...39

Figure 9 Location of study area of Debre Tabor town...44

LIST OF TABLES

Tables page

Table 1 Socio-demographic, hygienic habit and clinical information of patients with HIV/AIDS who taking ART at Debre Tabor General Hospital (DTGH), ART clinic 51

Table 2: Intestinal Parasites detected in HIV/AIDS patients who are on ART in DTGH. 53

Table 3 the Prevalence of mixed infection of coccidian parasite with non-opportunistic intestinal parasites among HIV/AIDS patient in DTGH 53

Table 4 Prevalence of Major Opportunistic Intestinal Protozoan Parasitic Species by age and sex among HIV/AIDS patients Who Visited DTGH ART clinics 55

Table 5: Bivariate and multivariable logistic regression analysis of opportunistic intestinal parasitic infection with predicted risk factors among ART following patients in DTGH. 58

Table 6: The association of each coccidian parasites with $CD4^+$ T-cell count status of HIV-infected patients who are on ART in DTGH. 61

LIST OF ABBREVIATIONS AND ACRONYMS

AIDS	Acquired Immune Deficiency Syndrome
AOR	Adjusted Odds Ratio
ART	Antiretroviral Therapy
CDC	Center for Disease Control and Prevention
CD4	Cluster of Differentiation 4
CI	Confident Intervals
COR	Crude Odds Ratio
DTGH	Debre Tabor General Hospital
GI	Gastrointestinal
HAART	Highly Active Anti-Retroviral Therapy
HIV	Human Immunodeficiency Virus
IPIs	Intestinal Parasitic Infections
OIs	Opportunistic Infections
OIP	Opportunistic Intestinal Parasites
OIPI	Opportunistic Intestinal Parasitic Infection
OIPPI	Opportunistic Intestinal Protozoan Parasitic Infection
PCR	Polymerase Chain Reaction
PLWHIV	People Living with Human immunodeficiency virus
SPSS	Statistical Packages for Social Sciences
UNAIDS	Joint United Nation Program on AIDS
WHO	World Health Organization

LIST OF APPENDICES

Appendices pages

Appendix A: The prevalence of opportunistic intestinal protozoan parasites in Ethiopia. 81

Appendix B: Questionnaire to be completed by Study Participants (English version) 94

Appendix C: Written Consent Form (English version) 96

Appendix D: Questionnaire (Amharic version)...97

Appendix E: Consent Form (Amharic Version) 98

Appendix F: Ethical clearance 100

ABSTRACT

Opportunistic intestinal parasitic infections cause significant morbidity and mortality among HIV infected people due to the down regulation of the immune system. In Ethiopia the burden of this infection is high due to poor personal and environmental hygiene. The present study aimed to find the prevalence and associated risk factors of opportunistic intestinal parasitic infections (OIPIs) in HIV/AIDS patients attending antiretroviral therapy in Debre Tabor General Hospital. A hospital-based cross-sectional study was conducted among patients attending Debre Tabor General Hospital Antiretroviral Therapy (ART) Clinic from December 2019 to February 2020. Direct wet mount, formol-ether sedimentation, and modified Ziehl-Neelsen staining techniques were used for parasitological examinations. Data on CD4+T-cell counts were taken from the patients' medical records. Socio-demographic characteristics and potential associated risk factors for OIPIs were collected using structured and pretested questionnaires survey. All the data were analyzed using SPSS version 23. Three hundred and eighty-four ART patients participated in the study. The overall prevalence of intestinal parasitic infections was 31.17% while that of OIPIs alone was 17.9%. Among identified intestinal parasites, *Cryptosporidium* species accounts for the highest frequency (33/384, 8.59%), followed by *Cystoisospora belli* (26/384, 6.77%), and *Entamoeba histolytica/dispar* 19/384, 4.9%). Being rural residence (AOR=0.197, 95% CI: 0.053-0.734, P= 0.015, CD4+ T-cell count <200cell/µl (AOR=49.08, 95% CI: 9.440-228.777, P= 0.000), poor adherence of ART (AOR=7.427, 95% CI 2.488-22.172, P=0.00), diarrheal history less than three-month (AOR=7.063, 95% CI: 1.88226.512 P=0.004), regular trimming of the fingernail (AOR=3.665 95% CI: 1.040-12.918 P=0.043), lack of hand washing habit after toilet (AOR=10.409 95% CI: 1.398-77.497 P= 0.022) and drinking water from the unprotected source (AOR=14.721, 95 % CI: 3.349-64.71 P=0.000) were determinant factor for OIPIs. In conclusion, the study indicated that the co-infections rates of OIPs remain a considerable problem that requires improvement in routine screening for OIPI among ART patients, particularly with those with poor or declining $CD4^{+}$ T cell counts.

Keywords: Antiretroviral therapy, HIV/ AIDS patient, Opportunistic intestinal parasite, Ethiopia

1 INTRODUCTION

1.1 Background

Opportunistic intestinal parasitic infections (OIPI) are infections of parasite species that cause moderate or asymptomatic intestinal infections in immunocompetent people; however, they are fatal in immunocompromised individuals (Al-Qobati *et al.,* 2018). Because of the progressive decrease in immunity, human immunodeficiency virus (HIV) infected individuals are at greater risk of acquiring several opportunistic intestinal infections (OIPI) https://en.wikipedia.org/wiki/HIV/AIDS. In HIV/AIDS patients, gastrointestinal (GI) infections are quite common, and diarrhea may be a common clinical condition seen in them (Rao, 2016). As per the available literature, the incidence of GI infections in HIV/AIDS patients ranged between 30% and 90% (Hafih, 2018).

Several studies have suggested that coccidian parasites (*Cryptosporidium* spp, *Cystoisospora belli and Cyclospora cayetanensis), Microsporidia* species, and *Blastocystis hominis* are parasites frequently associated with HIV seropositive individuals. They also named OIPI (Zeynudin *et al.,* 2013; Laksemi *et al.,* 2020). They are important causal agents for gastrointestinal disorders like chronic/persistent diarrhea, malabsorption, dehydration, weight loss, and wasting (Ayeh Kumi *et al.,* 2009). This parasite reproduces both sexually and asexually in the intestinal epithelium of the host. The transmission also occurs through the fecal-oral route by ingesting food or water contaminated with oocysts (CDC, 2019).

Although there has been an improvement with the introduction of Antiretroviral Therapy (ART) for individuals with Human Immunodeficiency Virus/Acquired Immunodeficiency Syndrome (HIV/AIDS), the presence of OIPs creates a serious challenge in reducing associated morbidity and mortality. The prevalence has also continued to be reported in people living with HIV (PLWHIV) across the globe including Ethiopia (Faye *et al*., 2010; Mehdi *et al*., 2020).

Cryptosporidium is an intracellular obligate intestinal coccidian parasite that causes cryptosporidiosis. Over half of (50.08%) of the water-borne diseases were caused by *Cryptosporidium* species (Painter *et al.,* 2016). The infections remain as typical causes of constant diarrhea in patients with AIDS in developing nations, and with up to 74% of diarrheal stools from patients with AIDS showing the organism. In developed nations with

low paces of natural pollution and far-reaching accessibility of intense antiretroviral treatment, the frequency of cryptosporidiosis has diminished in an incidence of <1 case per 1000 person per year in-patient with AIDS (Buchacz *et al*., 2016). Most researchers reported that the prevalence of *Cryptosporidium* ranges between 30%-60% in industrialized countries and it reaches up to 95% in developing countries (Meyers, 2014).

From India, *Isospora* was detected in a range of 16% to 47% of patients with HIV with diarrhea. In two of the studies, 50% and 81.8% of individuals with *Isospora* infection had $CD4^+$ T lymphocyte ($CD4^+$) counts < 200 cells/µl (National Institutes of Health., 2019). *Cyclospora cayatenesis* was reported with a prevalence rate of 41.6% in Peru (Burstein, 2005). Another deliberate audit of 27 examinations from 14 sub-Saharan Nations, revealed a general prevalence of 18% (Fletcher *et al*., 2011).

In Ethiopia, setting the epidemiology of coccidian parasites in HIV/AIDS patients were reported by different researchers. According to the systematic and meta-analysis of Mehdi *et al* (2020) done from 2001 to 2019, the overall pooled prevalence estimate of *Cryptosporidium* infection among People Living with Human Immunodeficiency Virus (PLWHA) in Ethiopia was 11%. A single study from Bahir Dar also reported a 46% prevalence rate of *Cryptosporidium* species among HIV positive patients.

Cystoisospora belli infection was reported by three groups of researchers in a different part of Ethiopia in a different time with prevalence of 22.5%, 12% and 15.3% in HIV infected patients (Endeshaw Tokola, 2005, Abebe Alemu *et al*, 2011 & Shimelis Assefa *et al*., 2009) respectively. Isosporiasis is more prevalent worldwide in children, but the exact prevalence of the disease is not known (Alemeria *et al.,* 2019).

There are no adequate works of literature on the epidemiological information's of *Cyclospora cayatenesis* but a few studies reported 2.8%, 3.7%, and 5.9% prevalence among HIV/AIDS patients (Mohamed Awole *et al* 2003; Menbereleul Mathwose *et al*., 2014 & Gethanu Alemu *et al*., 2018) respectively. Generally, the epidemiology of coccidian parasites among HIV/AIDS patients in the sub-Sahara region is still poorly understood (Echoru *et al*., 2015).

Variations in the prevalence of infection were influenced by study design, geographic area, age, the immunologic status of the population studied, seasonal variability of the parasite, methods of detection used, and expertise of the microscopes (Chacin-Bonilla, 2010; Almeria

et al., 2019). Limited research in Ethiopia, indicated that OIPIs are associated with lower $CD4^+$ T cell count (<200 cells/μl), poor level of environmental sanitation and personal hygiene plus contamination of food and drinking water. This happens because of improper disposal of human and animal excreta. Patients are not also frequently been screened for these pathogens on regular follow-up at monitoring clinics (Gethanu Alemu *et al*., 2018). Therefore, they occur wherever there is poverty (Getachew Hailemariam *et al*., 2004; Sayyari *et al*., 2005 & Laksemi *et al*, 2020).

Data regarding the extent of opportunistic intestinal parasitic infections among HIV/AIDS patients is rare in different parts of Ethiopia, particularly in Debre Tabor district. Because of the shortage of clean water in many villages of rural parts of Ethiopia, the community was forced to use unprotected water. In such areas where people use water from different sources, the possibility of infections with various water-borne diseases was expected to be extremely high. Also, there was no information on the prevalence and magnitude of the OIPI among HIV/AIDS patients in the present study area. Therefore, the present study was conducted to fill the existing gap and enable interested ones in understanding the prevalence of OIPI among HIV patients and major risk factors that could predispose HIV patients to these debilitating parasitic infections among HIV patients in DTGH, Northwest, Ethiopia.

1.2 Statement of the Problem

The crisis and pandemic spread of HIV/AIDS has been the greatest challenge to public health in many developing countries including Ethiopia. Globally at the end of 2018, 770,000 people died due to AIDS-associated ailments and 37.9 million people had been living with HIV (UNAIDS, 2019). Researchers estimated that 80% of the deaths from AIDS were attributed to opportunistic infections rather than the virus itself, and of these, up to a third of death from AIDS was attributed to OIPI (Mehdi *et al*., 2020). In the evolution of HIV infection, gastrointestinal involvement was common and 90% of the patients were related to gastrointestinal illness (Céline *et al.*, 2013).

Nevertheless, with the introduction of combination antiretroviral therapy and also more effective prophylaxis against these opportunistic infections, the rate of infections has decreased. However, patients in resource-limited settings with advanced asymptomatic diseases, and very low blood $CD4^+$ T-cell counts have been advised to start the ART programs (Missay Assefa *et al*., 2013). Besides, due to poor adherence to ART OIPIs still

represent frequent causes of morbidity and mortality in most developing countries. Scholar reports indicated that the rate of adherence to ART in Ethiopia was 88.2%, which was low compared to the WHO Standard (>95%) (Muktar Abadiga, 2019).

Several studies in Ethiopia have evidenced that the prevalence of OIPs among those patients is still high. A study done in Bahir Dar city, Northwest Ethiopia on HIV sero-positive individuals indicated that the prevalence of opportunistic intestinal protozoan parasitic infections was 62.6% (Abebe Alemu *et al*., 2011). However, the prevalence of this parasite was not fully understood in other different corners of Ethiopia, because some of the diagnostic methods for those newly emerging opportunistic intestinal parasites were not available to peripheral health institutions (Abebech Yitagesu, 2018). Hence, information about the co-infections rates of OIPs and HIV/AIDS among patients attending ART was very limited. Particularly in Debre Tabor General Hospital (DTGH) ART clinics, there was no published epidemiological data that indicates its magnitude and risk factors. In this regard, the present study was initiated to assess the magnitude of OIPIs and associated risk factors among ART following patients at Debre Tabor General Hospital, Ethiopia.

1.3 Objectives of the study

1.3.1 General Objective

The main objective of this MSc thesis was to determine the prevalence and associated risk factors of opportunistic intestinal parasitic infections among HIV/AIDS patients attending ART at Debre Tabor General Hospital, Amhara Region, Ethiopia.

1.3.2 Specific Objectives

1. To identify the major opportunistic intestinal parasite species among HIV/AIDS patients who were attending ART at Debre Tabor General Hospital from December 2019 to February 2020

2. To determine the prevalence of opportunistic intestinal parasite infections among the HIV/AIDS patients were attending ART at Debre Tabor General Hospital.

3. To identify the major associated risk factors for the prevalence of OIP infections among the HIV/AIDS patients attending ART at Debre Tabor General Hospital.

1.4 Significance of the Study

The finding of this study would provide preliminary data to show which intestinal parasites are commonly found in the study area. It has a valuable contribution to the program planners, scholastic network, service providers, and health care professionals.

Above all the HIV/AIDS patients who were experiencing OIP infections would benefit from this study of controlling and recognizing right on time to bring down their morbidity and mortality because of this incapacitating infection. This examination will likewise make mindfulness for society, what are the major related risk factors that expose HIV/AIDS patients to OIP diseases.

1.5 Limitation of the study

In the present study, there were limitations. Due to resource constraints, this study was not able to perform sensitive tests like PCR for confirmation and the specific species of parasites primarily for *Microsporidium*. Secondly, the prevalence estimates of OIPIs in this investigation was based on a single stool sample, this may affect the estimation.

2 LITERATURE REVIEW

2.1 Opportunistic Intestinal Parasites in HIV/AIDS Infected Individual

Because of the progressive decrease in immunity, HIV-infected individuals are highly at risk of opportunistic intestinal parasitic infections (OIPI). In HIV patients, gastrointestinal (GI) infections are quite common, and diarrhea may be a common clinical condition seen in them. As per the available literature, the incidence of GI infections in HIV patients ranged between 30% and 90% (Hafiz, 2018).

Various enteric pathogens caused GI infections in HIV patients. Among these, parasites were important, and that they caused acute chronic diarrhea and weight loss. Common parasites that caused GI infections in HIV patients were *Cryptosporidium parvum, Cyclospora cayetanensis, Cystoisospora belli , Microsporidia, Strongyloides stercoralis, Entamoeba* species, and *Giardia lamblia* (Babu *et al.*, 2017). Some protozoan parasites like *Cryptosporidium, Cystoisospora belli, Microsporidian species, Cyclospora cayetanensis,* and *Blastocystis hominis* are frequently associated with acute and chronic diarrhea in HIV seropositive individuals. They also termed opportunistic intestinal parasitic infections (Zelalem Tekel-Mariam *et al.*, 2008).

Protozoan parasites are single-celled organisms, which are widely prevalent causing considerable public health problems in developing countries, especially in tropical and sub-tropical regions. Although the bulk of intestinal protozoan occurs as a free-living organism within the soil, a considerable number of protozoan parasites also exist as mutualism, commensalism, or parasitism (Gillespie, 2001). Numerous protozoan species inhabit the digestive tube of humans. The majorities of intestinal protozoa are non-pathogenic or only lead to mild disease. A number of these organisms can cause severe disease and life-threatening diarrhea in AIDS patients and other immune-compromised individuals (Chen *et al.*, 2012).

These opportunistic parasitic infections are linked to the absence of sanitation, lack of access to safe water, and improper hygiene, therefore, they occur wherever there is poverty (Sayyari *et al.*, 2005). Intestinal parasitic infections have public features that are endemic in inhabitants with low socioeconomic status, overcrowding, and poor hygiene, favorable warm, and tropical conditions for the transmission of the parasites. Limited access to clean water supply, improper disposal of human faces, malnourishment, lack of adequate healthcare

services, and peasant farming, favoring larval skin penetration, and oral-fecal spread (Getachew Hailemariam *et al*., 2004).

These factors are the reason behind the foremost proportion of the burden of the disease and death in developing countries (Mazigo *et al*., 2010). The parasites are important causal agents of gastrointestinal disorders like diarrhea, dysentery, vomiting, lack of appetite, malabsorption, weight loss, abdominal cramp/pain; flatulence's and related in HIV/AIDS infected individuals (Ayeh Kumi *et al*., 2009).

2.2 Reason of Intestinal Protozoa more commonly Seen in Immuno-compromised Hosts

Immunocompromised hosts are at higher danger for acquiring intestinal protozoan infections particularly within the placing of impaired or poor T-cellular function. The immune response in opposition to parasites is divided into two vast classes: innate immunity, which on my own seldom eliminates the parasite; and adaptive immunity, which is better suitable to thwarting the infection (maizels, 2009).

Intestinal protozoa commonly trigger a strong adaptive immune reaction mediated by way of T- cells. However, the immune reaction may not constantly be powerful. Those parasites have advanced mechanisms to prevent the immune reaction and continue to exist in the host. As an example, parasites can modulate the effector reaction utilizing inducing regulatory T-cells, which in turn can suppress anti-parasitic effector cells. The underlying mechanisms using which the intestinal parasites invade and cause infection within the immuno-compromised host to stay poorly understood (Marcos & Gotuzzo, 2013; Thom & Forrest, 2006).

2.3 Opportunistic parasitic infections in patients with human immunodeficiency virus/acquired immunodeficiency syndrome

Opportunistic intestinal parasitic infections are infections of parasite species that are mild or asymptomatic in immunocompetent humans; but, in immuno-compromised human beings, they come to be deadly. Opportunistic parasitic infections, which include worms and protozoa, are ignored tropical diseases that are targeted by using the sustainable improvement dreams to be eliminated through 2030 (Laksemi *et al,* 2020). Human Immunodeficiency Virus (HIV) infection causes acquired immunodeficiency syndrome (AIDS), that is a

complicated disorder in humans, and suppresses the immune functions (De silva *et al*., 2005; Shimelis Assefa *et al*., 2009; Noor *et al*., 2012 and Missaye *et al*., 2013).

Opportunistic parasitic infections are one of the maximum issues in patients with HIV/AIDS. The presence of opportunistic parasitic infections in HIV sufferers suggests that they are within the section of AIDS. Most of those infections are excessive and frequently contribute to the loss of life of inflamed individuals. Consequently, early detection and remedy need to be done to ensure good enough control (Shenoy *et al*., 2017).

Some opportunistic infections, parasites are the maximum usual causative pathogens that affect the morbidity and mortality of sufferers with HIV/AIDS. Parasites can trigger an immune response to contaminate the respiration tract, digestive tract, blood, brain, and other organs. The parasitic species that most customarily cause the opportunistic infections inside the human body are *Toxoplasma gondii, Cryptosporidium parvum, Cystoisospora belli, Cyclospora cayetanensis, Microsporidia, Cryptococcus neoformans, Pneumocystis carinii or jiroveci,* and *Entamoeba* (Laksemi *et al*., 2020).

UNAIDS facts at the current HIV/AIDS situation have mentioned that globally 1.7 million people became newly infected with HIV by at the stop of 2018, 37.9 million people globally had been dwelling with HIV with the aid of the give up of 2018 of these 800,000 (28%) are residing in the east and south Africa. Globally 770,000 humans have died from AIDS-associated ailments. From this determination, 62 % of 23.3 million humans are receiving treatment globally. The prevalence fee in Ethiopia stages from 0.7%-1.4% (UNAIDS 2019).

Consequently, the co-infection of HIV/AIDS opportunistic parasitic contamination is a first-rate purpose of morbidity and mortality international; a maximum of them are raising diseases. Know-how of parasites that motive opportunistic infections in HIV/AIDS is needed, specifically within a long time, where HIV has triggered a huge burden on worldwide wealth and health. Accordingly, early detection and spark of treatment may be mounted to reduce deaths due to HIV/AIDS (Nissapatorn & Sawangjaroen, 2011: Laksemi *et al*., 2020).

This overview affords a top-level view of the etiologic agent of opportunistic infections amongst nations, epidemiology, and occurrence, lifecycle, treatment, exam strategies, prevention, and remedies.

2.4 Risk Factors for Opportunistic intestinal parasitic infections

Contamination threat elements for HIV/AIDS are man, unemployment, living in an urban area, and marriage. However, other studies have observed that women, homemakers, and trading are threat factors for HIV/AIDS. In HIV/AIDS sufferers, the price of a specific intestinal parasitic contamination relies upon at the endemicity of the parasite inside the community (Rao, 2016; Varatharajalu & Kakuturu, 2016). Intestinal parasites are broadly Distributes partially because of the low degree of environmental and personal hygiene, fecal contamination of meals and ingesting water, and poor housing centers (Laksemi *et al*., 2020). *C.parvum* and that *C.belli* may be transmitted from human to human through anal-oral contact (Laksemi *et al*., 2020).

Assessing cutting-edge $CD4^+$ cell count allows us to become aware of the fame of intestinal parasite infection amongst HIV sufferers. Chance factors related to a higher prevalence of opportunistic intestinal parasitic infection amongst HIV patients have been low $CD4^+$ counts, persistent diarrhea, poor residing conditions, and bad nutrients. Another research stated employment Status, $CD4^+$ T-cell count, diarrhea, working status, and get access to a latrine because of the threat factors related to a higher incidence of intestinal parasites amongst HIV patients (Laksemi *et al*., 2020).

The association between the $CD4^+$ T-cellular be counted and intestinal protozoa infection has additionally been mentioned in the preceding research from Ethiopia, India, and Malaysia. Degree of HIV/AIDS, $CD4^+$ level, the status of adherence, and hemoglobin stage has also been suggested as chance elements for opportunistic intestinal parasitic infections. CD^+ count <200 cells/µl poses the best danger for opportunistic infection in HIV/AIDS patients (Gedle Derje *et al*., 2017).

Threat elements for *Cyclospora cayetanensis* are the intake of uncooked culmination and veggies, drinking untreated water, swimming in rivers, contact with soil or animals, agricultural paintings, and terrible hygiene (Laksemi *et al*., 2020).

Danger elements for *C.parvum* infection are non-Hodgkin's lymphoma, leukemia, lymphoproliferative ailment, malnutrition, immunosuppressive tablets, cancers, and hemodialysis. Risk elements for *C.belli* encompass lymphoblastic leukemia, grownup T-cell

leukemia, Hodgkin's sickness, non-Hodgkin's lymphoma, lymphoproliferative disorders, renal transplant, and liver transplant (Faisal and Bokhari, 2020).

Risk factors of *Blastocystis hominis* are common, and anyone can have the organism in his or her stools. You might be at higher risk if you travel or live where hygiene is inadequate or where the water might not be safe or if you handle infected creatures, such as pigs and poultry (Tan *et al*., 2008).

Risk factors associated with *Microsporidia* contamination include sexual members of the family among guys, use of intravenous capsules, exposure to swampy water or irrigated areas, publicity to water with feces, and habit of swimming pools and hot tubs, or contact with water (Faisal and Bokhari, 2020).

Intestinal protozoan parasites are transmitted using the fecal-oral route and tend to exhibit similar life cycles consisting of a cyst/oocyst and trophozoite stages. This involves the ingestion of food or water contaminated with cysts/oocysts. Some of the trophozoites will develop into cysts/oocysts instead of undergoing replication. Factors that increase the chance of ingesting materials contaminated with fecal material play a role in the transmission of these intestinal protozoa. In general, situations involving close human-to-human contact and unhygienic conditions promote transmission (Chen *et al*., 2007; Laksemi *et al*., 2020).

2.5 Clinically Relevant opportunistic Intestinal Parasitic Infections

Parasitic infections in the digestive tract continue to be a burden for human beings infected with HIV, even inside the era of the use of antiretroviral therapy (ART). $CD4^+$ relies on HIV patients is a chance component for opportunistic parasitic infections with manifestations of diarrhea. In AIDS with excessive immunosuppression, commensal intestinal parasites end up opportunistic, inflicting fatal prognostic diarrheal diseases in HIV patients (Harms & Feldmeier, 2002).

Numerous species of parasites motive diarrhea in HIV patients, which includes *C. parvum, Giardia lamblia, Microsporidia*, and *C.belli* a few studies have reported that the most not unusual intestinal parasitic species located in diarrhea experienced through HIV sufferers are *Blastocyst species., C. parvum, Microsporidia, and S. stercoralis*. There also are parasites called classical opportunistic agents, which can be *C.parvum, I.belli, Cyclospora cayetanensis*, and *Microsporidia* (Harms & Feldmeier, 2002).

2.5.1 Cryptosporidiosis

Cryptosporidiosis is caused by an intracellular obligate protozoan parasite called *Cryptosporidium* species. Starting in 2019, there were in any event 35 named *Cryptosporidium* species, as perceived by the host specificity, morphology, and atomic science considers. Other than people, the parasite can contaminate numerous different types of creatures, for example, warm-blooded animals, winged creatures, reptiles, and is pathogenic to immunocompetent and immunocompromised hosts like HIV/AIDS patients (khan*et al*., 2018; Nadar *et al*., 2019).

2.5.1.1 Morphologic Description

Entirely four steps of *Cryptosporidium* (trophozoite/type I meront, type II meront, microgamont, and macrogamont/ oocyst) form spherical or cone-shaped cells within epithelial cells lining the host's gastrointestinal tract. Except for unfertilized macrogamonts, each mature stage produces a smaller banana or bullet-shaped penetrating form that is asexual (sporozoite, type I merozoite, type II merozoite) or sexual (microgamete) (Klassen-fischer *et al*., 2008).

Trophozoites are one µm to 2.5 µm and contain eight types I merozoite. The parasitophorous vacuole lies in the microvillus border of the cell, just below the plasma membrane. This intracellular but extra cytoplasmic location differs from that of related coccidian that resides in intracytoplasmic vacuoles (Klassen-fischer *et al*., 2008).

Type II meronts are 3.5 µm and contain four type II merozoites. The nuclei of first- or second-generation meronts become smaller during division and migrate toward the periphery. Microgamonts are two µm and contain 14 to 16 peripherally arranged microgametes. Macrogamonts are spherical and are four µm x 5 µm. They contain a large, centrally placed nucleus with a prominent nucleolus (Klassen-fischer *et al*., 2008).

Mature oocysts of *Cryptosporidium* are spherical, refractile, 5 µm x 7 µm and contain four sporozoites. Each of the four sporozoites is 2.4 µm x 0.69 µm to 4.5 µm x 0.95 µm and has no sporocyst. Merozoites are 0.4 µm x 1 µm and contain a Golgi apparatus, endoplasmic reticulum, and nucleus. Microgametes are bullet or rod-shaped not more than 1 µm to 2 µm long. Unlike some other members of this phylum, *Cryptosporidium* microgametes have no flagellum (Klassen-fischer *et al*., 2008).

2.5.1.2 Epidemiology

The first manifestation of cryptosporidiosis was reported by Nine *et al* (1976), and the diseases become the major concern when notification of the first 21 patients was given, 14 who died of chronic diarrhea caused by *Cryptosporidium* species infection (Cimerman *et al*., 1999).

Ingestion of defiled water has been liable for huge episodes of *Cryptosporidium*. The biggest overall episode revealed in Milwaukee in the USA in 1993 brought about a gauge of >400 000 individuals influenced. In England, episodes have primarily been accounted for related to open and private water supplies and pools, with the Boucher report giving suggestions on lessening such dangers (Horne *et al*., 2017).

Control of these episodes is trying because of various transmission courses; the exceptionally irresistible nature of the parasite, and opposition of the oocysts to chlorine-based disinfectants at levels ordinarily utilized in most pools (Naseer *et al*., 2018). Timely announcing of suspected cases by essential consideration can help general wellbeing associates distinguish whether they are associated with a source for example pools to control a potential outbreak (as found in the recent outbreak in the West Midlands) (Horne *et al*., 2017).

Contamination happens through the ingestion of *Cryptosporidium* oocysts. Appropriate oocysts in excrement can be transmitted legitimately through contact with people or creatures tainted with *Cryptosporidium*, especially those with looseness of the bowels. *Cryptosporidium* oocysts can defile recreational water sources, for example, pools, lakes, open water supplies, and may continue despite standard chlorination (Cama *et al*., 2007).

Cryptosporidium causes approximately 250 to 500 million cases of diarrhea per year in developing nations of Asia, Africa, and Latin America. *Cryptosporidium* is the most common parasitic cause of diarrhea in the United Kingdom and infected 28,636 persons in the United States between 2006 and 2008 (Klassen-Fischer *et al*., 2008).

The asymptomatic carriage rate may be as high as 13% among immunocompetent individuals. Cryptosporidiosis develops in an estimated 10-15% of patients with AIDS in the United States and 30%-50% of patients with AIDS in the developing world. The serological prevalence of *Cryptosporidium* ranges between 30%-60% in industrialized countries and reaches 95% in tropical and developing countries (Klassen-fischer *et al*., 2008).

The other recent study revealed that the prevalence rate of cryptosporidiosis among diarrhea patients with HIV/AIDS is significantly higher than 10%. Immunocompromised diarrhea tends to be chronic and causes increased morbidity and mortality in these patients (Nadar *et al.*, 2019).

The parasite did not only cause diarrhea but it also manifested several symptoms in the lungs of HIV/AIDS patients. Coccidian parasites (*Cryptosporidium* species, *C.belli*, and *Cyclospora* species) are the most common enteric parasites in immunocompromised patients, which can cause severe, deadly diarrhea (Nadar *et al*., 2019).

The three species that most ordinarily contaminate people are *Cryptosporidium hominis, Cryptosporidium parvum, and Cryptosporidium meleagridis.* Diseases are typically brought about by one animal varieties, yet combined contamination is possible (Cama *et al*., 2007; Wanyiri *et al*., 2014).

Cryptosporidiosis stays a typical reason for constant Diarrhea in patients with AIDS in creating nations, with up to 74% of diarrheal stools from patients with AIDS showing the organism. In created nations with low paces of natural pollution and far-reaching accessibility of intense antiretroviral treatment, the frequency of cryptosporidiosis has diminished. In the United States, the occurrence of cryptosporidiosis in patients with HIV is currently <one case per 1,000 individuals' years (Buchacz *et al*., 2016).

Even though the contamination is self-constraining in many patients, in the extremely youthful, more seasoned individuals, and immunosuppressed people, it very well may be a weakening sickness. *Cryptosporidium* happens worldwide and is the fourth most basic reason for gastrointestinal contamination in the United Kingdom (Cama *et al.*, 2007).

Because of the expanding significance of the parasite, the WHO has remembered this for the Neglected Diseases Initiative as of late. As gastrointestinal manifestations can result from a scope of conditions, just some of which will be infectious, microbiological affirmation is required to affirm the determination (Horne *et al.,* 2017). Information accessible from the general strength of England for 2000 to 2012 shows that broadly the quantity of confirmed cases from tests ranges from 3000–6000 every year, with tops in the spring and autumn (Horne *et al*., 2017).

Youngsters more youthful than 2 years might be increasingly vulnerable to contamination, possibly due to expanded fecal-oral transmission right now and because of an absence of protective immunity. Waterborne pandemics in industrialized nations influence all ages (Wang *et al*., 2018 Ajjampur *et al*; 2008; Nair *et al*., 2008, & Wanyiri *et al*., 2014).

Person to person transmission of *Cryptosporidium* is normal, particularly among explicitly dynamic men who have intercourse with men. The hatching period changes, however, are overall between 5–7 days (Cama *et al*., 2007; Flanigan *et al.,* 1992). Disease, for the most part, brings about an intense ailment enduring as long as 3 weeks in any case sound individuals, albeit asymptomatic contaminations are normal and can be a wellspring of disease for other people (Cama *et al*., 2007).

2.5.1.3 Life Cycle and Transmission

Cryptosporidiosis is caused by the ingestion of mature oocyst of Cryptosporidium species through contaminated water, food, person to person contact with infected patients (family members, health care workers, users of communal swimming pools, travellers) and animal to human contact (Xiao and Ryan, 2008).

The life cycle of *Cryptosporidium* is monoxenous, completed within the gastrointestinal tract of a single host (Fayer *et al.,* 2018). The oocyst is the only exogenous stage and is approximately 4-6 µm in diameter, with distinct inner and outer layers and four fully developed and infectious sporozoites. Ingestion of oocysts initiates infections. After exposure to gastric acid bile salts and proteolytic enzymes in the upper gastrointestinal tract, excystation of the sporozoites occurs through a small intestine at the end of the oocyst wall. Released motile sporozoites probe and attach with their apical membrane (luminal surface) of enterocytes (Fayer *et al.,* 2018).

An infection has also been reported on other sites (often contiguous with the intestinal tract), such as the biliary tract, pancreatic ducts, sinuses and respiratory tract, which are also lined with epithelial cells. This ultimately places it in an intracellular but extracytoplasmic compartment below the cell's outer membrane, termed the 'parasitophorous vacuole' a 'feeder organelle' (located at the base of the parasitophorous vacuole) forms between the developing intracellular parasite and the host cell. This distinctive electron-dense structure is presumed to permit an exchange of molecules with the host cell (Xiao and Ryan, 2008).

After the invasion, sporozoites differentiate into rounded trophozoites, which after asexual reproduction (merogony or schizogony), become type I meronts (or schizonts) with six to eight merozoites are curved parasites with a double inner membrane and an apical complex of rings and micronemes. The rupture of type I meronts release mature merozoites, which can further invade adjacent epithelial cells and become either type I or II meronts. The cycling of type I meronts is thought to partially explain the ability of *C.parvum* to persist in the human host. Type II meronts have four merozoites that invade host cells to undergo sexual reproduction (gametogenic) and become male or female gamonts, which can be seen as early as 36 hours post-infection (CDC, 2019).

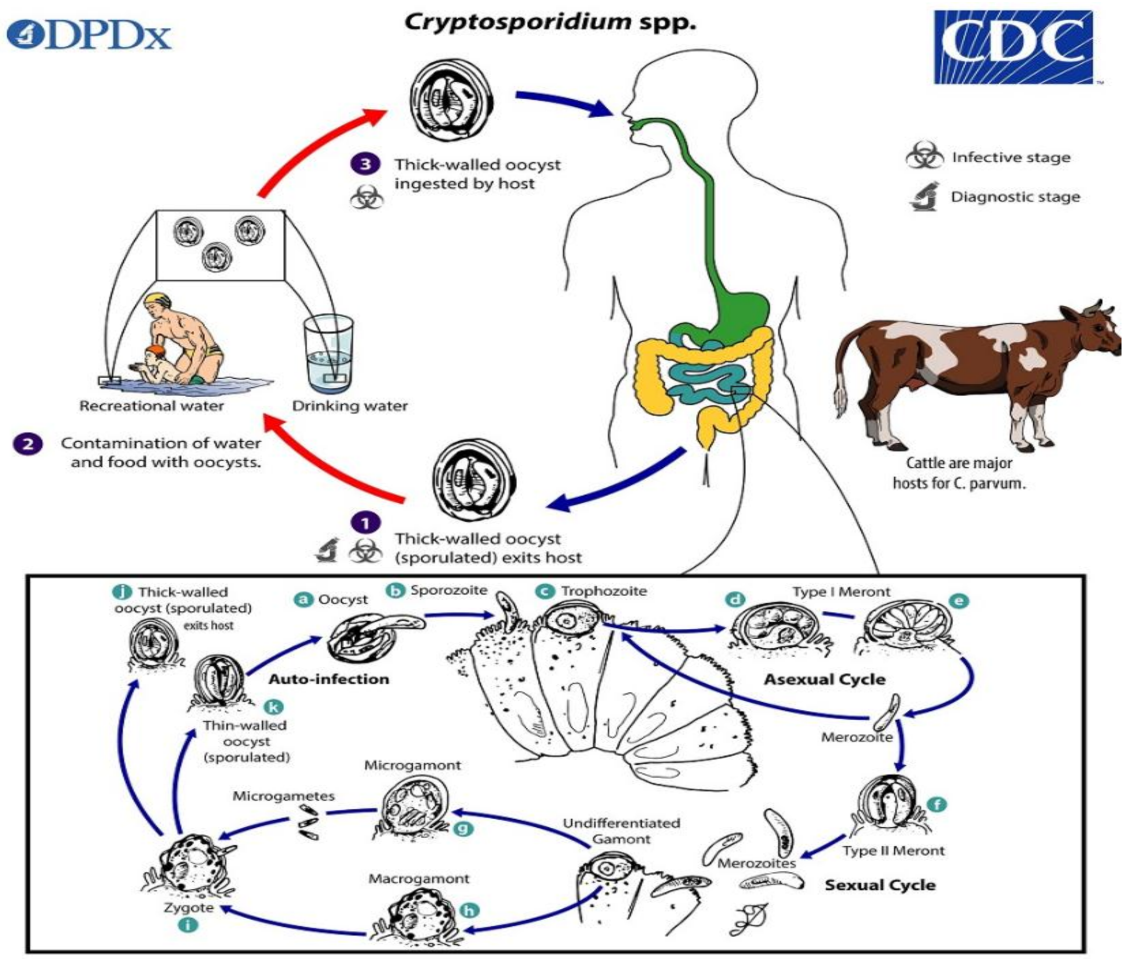

Figure 1: The Life cycle of *Cryptosporidium* species: Source: (CDC, 2019)

Mature micro (male) and macro (female) gamonts attach, fuse, and form the zygote, which develops into either a thick-walled or thin-walled oocyst, each with four fully infectious sporozoites. Thin-walled oocysts are associated with auto infections of the intestine, providing a second mechanism by which *C.parvum* can autoinfect the host, resulting in

persistent infections. In contrast, thick-walled oocysts are capable of surviving for long periods from oocyst ingestion to the excretion of infectious oocysts, which is approximately 4-22 days for humans as illustrated in figure one above (CDC, 2019).

2.5.1.4 Pathogenesis

Cryptosporidium oocysts are irresistible, requiring less than 10 oocysts to cause human sickness for some segregates. The oocysts are irresistible following discharge and the existing pattern of the parasite produces shapes that reinvade the digestive system. The area of the parasite in the digestive system is intracellular however extracytoplasmic, which may add to the stamped opposition of *Cryptosporidium* species to treatment. Huge quantities of oocysts are discharged and are resistant to brutal conditions, remembering chlorine at levels typically applied for water treatment (Okhuysen *et al*., 1999; Chappell *et al*., 2006).

Upon oocyst excystation, four sporozoites are discharged which follow their apical closures to the outside of the intestinal mucosa (Keusch, *et al.,* 1995). A sporozoite-explicit lectin adherence factor has been recognized as the operator of connection to the intestinal surface (Keusch, *et al.,* 1995). After the sporozoite connection, it has been estimated that the epithelial mucosa cells discharge cytokines that activate inhabitant phagocytes (Goodgame, 1996).

These initiated cells discharge dissolvable variables that expand intestinal emission of water and chloride and restrain ingestion. These dissolvable variables incorporate histamine, serotonin, adenosine, prostaglandins, leukotrienes, platelet-enacting element, and they follow up on different substrates, including enteric nerves and the epithelial cells themselves (Goodgame, 1996).

Thus, epithelial cells are harmed by one of two models:
Cell demise is immediate after effect of parasite intrusion, duplication and expulsion or Cell harm could happen through T cell-intervened aggravation, delivering villus decay, and grave hyperplasia (Goodgame, 1996). Produces a twisting of villus design or joined by supplement malabsorption. Test proof supporting this pathogenic speculation exists in a pig model framework, where diminished intestinal sodium retention has been corresponded with "both diminished villus surface zone and hindrance by prostaglandin E2 created by provocative cells" (Goodgame, 1996).

2.5.1.5Clinical Manifestations

Patients with cryptosporidiosis most normally have the intense or subacute beginning of watery looseness of the bowels, which might be joined by vomiting, heaving, and lower stomach squeezing. Ailment seriousness can go from asymptomatic to plentiful, cholera-like Diarrhea (Checkley *et al.*, 2015).

More extreme indications will in general happen in vulnerable stifled patients, while transient looseness of the bowels alone is normal in patients with skillful resistant frameworks. Fever is available in around 33% of patients and malabsorption is normal. The epithelium of the biliary tract and the pancreatic conduit can be contaminated with *Cryptosporidium*, prompting sclerosing cholangitis and to pancreatitis optional to papillary stenosis, especially among patients with delayed infection and low $CD4^{+}$ checks (Naseer *et al*., 2018). Pulmonary *Cryptosporidium* contaminations additionally have been accounted for and might be under-perceived (Reina *et al*., 2016).

2.5.1.6 Diagnosis

Diagnosis of cryptosporidiosis has generally been made by minuscule distinguishing proof of the oocysts in the stool with corrosive quick recoloring or direct immunofluorescence, which offers higher affectability (Garcia *et al*., 2018).

Fixation strategies (e.g., formalin-ethyl acetic acid derivation) may encourage the diagnosis of cryptosporidiosis. Other indicative techniques are large progressively utilized. Antigen-recognition by chemical connected immunosorbent examines or an immunochromatographic test likewise is valuable; contingent upon the particular test, sensitivities allegedly run from 66% to 100%. Nevertheless, some immunochromatographic tests are tormented by bogus positive outcomes (Roellig *et al*., 2017).

Multiplex sub-atomic techniques are progressively utilized for analysis and can distinguish a more prominent number of cases than minute strategies. Cryptosporidial enteritis additionally can be analyzed from little segments of tissue from an intestinal biopsy (Ryan *et al*., 2017).

A solitary stool example is normally sufficient to conclusion cryptosporidiosis in people with plentiful diarrheal sickness, though rehash stool inspecting is prescribed for those with the milder disorder.

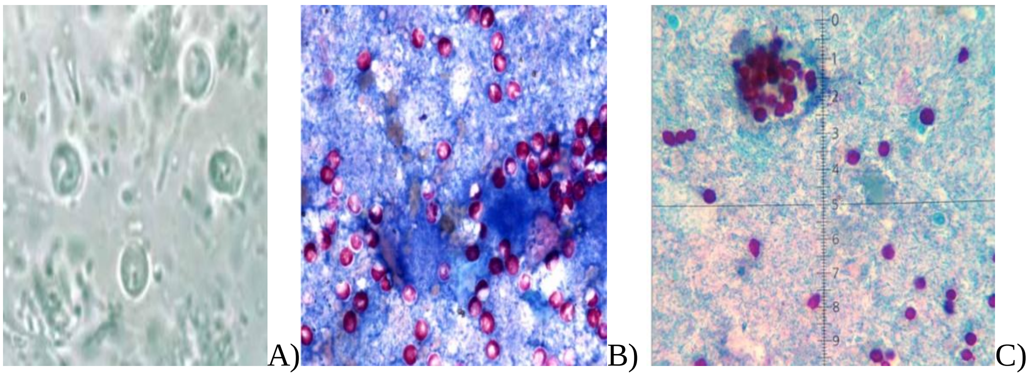

A) B) C)

Figure 2 Oocyst of *Cryptosporidium* in human faces

A) *Cryptosporidium* species in a wet mount. B) Oocyst of *Cryptosporidium* species from Ethiopia HIV/AIDS diarrhea patient stained with modified Ziehl Neelsen, magnification (1000X) (Endeshaw Tokola, 2005). C) Multiple *Cryptosporidium* species oocysts in the stool specimen in a polish traveller infected in India. Staining with a modified Ziehl-Neelsen method (Magn. 1000x) (Kłudkowska *et al.*, 2017)**.**

2.5.1.7 Prevention Exposure

People with HIV ought to be instructed and directed about the various ways that *Cryptosporidium* can be transmitted. Methods of transmission incorporate direct contact with individuals, including diapered youngsters, and creatures tainted with *Cryptosporidium*; gulping sullied water during recreational exercises; drinking defiled water, and eating debased nourishment (Huang & Zhou, 2007).

Trustworthy handwashing can diminish the danger of the runs in people with HIV, including looseness of the bowels brought about by *Cryptosporidium*. Patients with HIV ought to be encouraged to wash their hands after potential contact with human defecation (counting in the wake of diapering little kids). Handwashing likewise ought to be prescribed in relationship with the accompanying exercises: in the wake of taking care of pets or different creatures, after cultivating or some other contact with soil, before planning nourishment or eating, and when sex (Curtis & Cairncross, 2003).

People with HIV ought to maintain a strategic distance from unprotected sex, particularly rehearses that could prompt direct (e.g., oral-anal sex) or aberrant (e.g., penile-anal sex) contact with dung. They ought to be encouraged to utilize prophylactic obstruction strategies,

for example, condoms and dental dams during sex to lessen such exposures (Huang & Zhou, 2007).

According to the National Institutes of Health (2019), people with HIV especially those with $CD4^+$ tallies <200 cells/cells/µlought to maintain a strategic distance from direct contact with looseness of the bowels or stool from pets. They should wear gloves when taking care of excrement or cleaning regions that may have been debased by dung from pets. People with HIV ought as far as possible or maintain a strategic distance from direct presentation to calve and sheep.

Focusing on cleanliness and maintaining a strategic distance from direct contact with stool are significant when visiting farms, petting zoos, or different sites where animals are housed or displayed. People with HIV ought not to drink water straightforwardly from lakes or waterways. Waterborne disease likewise can come about because of drinking water during recreational exercises (National Institutes of Health, 2019).

According to the reports of National Institutes of Health (2019) People with HIV ought to be advised that lakes, waterways, salt-water seashores, some pools, recreational water parks, and decorative drinking fountains might be debased with human or creature squander that contains *Cryptosporidium*. They ought to abstain from swimming in water that is likely infected, and ought to abstain from swallowing water while swimming or playing in recreational water.

Notwithstanding, people with HIV may think about drinking just sifted water, despite the complexities engaged with choosing fitting water channels, the absence of enforceable guidelines for the exclusion of *Cryptosporidium* oocysts, the expenses of the items, and the trouble of utilizing the items reliably. Note that ice produced using polluted outlet water likewise can be a wellspring of disease (Huang & Zhou, 2007).

2.5.1.8 Prevention of Disease

Since endless cryptosporidiosis happens essentially in patients with cutting edge immunodeficiency, the initiation of ART before the patient turns out to be seriously immunosuppressed ought to forestall this malady (Holmberg *et al*., 1998; Fichtenbaum *et al*., 2000).

Rifabutin and possibly clarithromycin has taken for Mycobacterium avium complex prophylaxis has been found to secure against cryptosporidiosis. Rifaximin, which is utilized for counteraction of voyagers' Diarrhea, additionally has been utilized to treat cryptosporidial looseness of the bowels (Gathe *et al*., 2008). Nevertheless, it is indistinct whether rifaximin can ensure against cryptosporidiosis. Information is deficient, nevertheless, to warrant a suggestion to utilize rifaximin, rifabutin, or clarithromycin as chemoprophylaxis for cryptosporidiosis (Gathe *et al*., 2008).

2.5.1.9 Treating Disease

In the setting of extreme immune concealment, ART with safe rebuilding to a $CD4^+$ check >100 cells/cells/µl, for the most part, prompt goals of clinical cryptosporidiosis and is the backbone of treatment (Maggi *et al*., 2000; Schmidt *et al*., 2001 and Dillingham *et al*., 2009).

A few operators, including nitazoxanide, paromomycin, and spiramycin, have been researched in little, randomized controlled clinical preliminaries of grown-ups with HIV. No pharmacologic or immunologic treatment coordinated explicitly against *Cryptosporidium* has been demonstrated to be reliably compelling when utilized without ART (Cabada *et al.*, 2010).

In one investigation, grown-ups with HIV with cryptosporidiosis with $CD4^+$ checks >50 cells/cells/µl were treated with nitazoxanide 500 mg to 1,000 mg twice day by day for 14 days; the nitazoxanide treatment bunch had significantly higher paces of parasitological fix and goals of Diarrhea than the fake treatment gathering (Rossignol, 2006).

A meta-investigation of 11 distributed investigations of paromomycin in people detailed a reaction pace of 67%; nonetheless, there were barely any fixes, backslides were normal, and long haul achievement rates were just 33%. Two randomized preliminaries contrasting paromomycin and fake treatment exhibited restricted viability of the medication among patients with AIDS and cryptosporidiosis (White *et al*., 1994; Cabada *et al*., 2010; Article *et al*., 2000). One case arrangement recommended a superior reaction rate in patients getting paromomycin alongside ART (Paolo Maggi *et a*l., 2001). Paromomycin might be utilized rather than nitazoxanide related to ART, however never rather than ART (Maggi *et a*l., 2000).

2.5.1.10 Prevention Recurrence

No pharmacologic intercessions are known to be powerful in the prevention of repeat infections of cryptosporidiosis (National Institutes of Health, 2019).

2.5.2 Cyclosporiasis

Cyclosporiasis is an intestinal illness caused by the microscopic parasite *Cyclospora cayetanensis*. Cyclosporiasis is characterized by mild to severe nausea, anorexia, abdominal cramping, and watery diarrhea (Shields & Olson, 2003). People can become infected with *cyclospora* by consuming food or water contaminated with the parasite. People living or travelling in countries where cyclosporiasis is endemic may be at an increased rate of infections (Bhattachan *et al.*, 2017; Casillas *et al*., 2019).

2.5.2.1 Morphologic Descriptions

Cyclospora oocysts are eight µm to 10 µm. Hematoxylin-alone stained sections of duodenal biopsies show the intracellular stages. A later report recommends hematoxylin & eosin. All four asexual stages (sporozoite, trophozoite, schizont, and merozoite) have been observed in the enterocytes. Immature schizonts (uninuclear trophozoites) are 2 µm to 3 µm in diameter. Merozoites are banana-shaped and 5 µm to 6 µm long, with a nucleus in the posterior third. (https://www.merckmanuals.com/professional/infectious-diseases/intestinal-protozoa-and-microsporidia/cyclosporiasis).

2.5.2.2 Epidemiology

Cyclospora cayetanensis is an apicomplexan *coccidiam* in the family *Eimeriidae* closely related to Eimeria species. *Cyclospora cayetanensis* is the only known species of the genus *Cyclospora* to infect human infection results in enteric disease, primarily diarrhea, but asymptomatic infection has been observed. Humans are the only hosts known (Chacín-bonilla, 2010).

The methods of transmission of *Cyclospora cayetanensis are* still not recorded, even if the fecal-oral transmission is a significant course. Direct individual-to-individual transmission is impossible. Unusual transmission can happen if a contaminated individual pollutes nature, the oocysts sporulate under the correct conditions and afterward defiled food and water are ingested (Almeria *et al*., 2019).

In created countries, hazard components and methods of transmission have been distinguished. Most cases have been identified with global travel or to nourishment borne

episodes brought about by imported produce from endemic districts (Casillas *et al*., 2019; Mansfield & Gajadhar, 2004).

Conversely, the hazard factors and courses of spread for *Cyclospora cayetanensis* in creating territories remain ineffectively comprehended. Factors identified with water, eating fresh nourishment, contact with soil, horticultural occupations, absence of handwashing, and factors related to low financial status have been connected to disease (Chacin-Bonilla, 2010).

In Ethiopia, Vegetables and crude organic products (avocado, lettuce, cabbage, carrot, tomato, banana and mango) are archived as hazard factors. Parasitic sullying of crude vegetables and natural products gathered from chosen nearby markets in Arba Minch town, Southern Ethiopia (Yonatan Kindie & Shiferaw Bekele, 2016). *Cyclospora cayetanensis* disease has been accounted for around the world, in both created and creating nations; however, it is generally basic in tropical and subtropical zones. The first documented cases were found in Papua, New Guinea in 1977 and 1978 (Chacin-Bonilla, 2010). At first distinguished endemic territories were Haiti, Guatemala, Peru, and Nepal, where the primary episodes were accounted for. As of now, endemic territories are viewed as Central and South America, a few nations in the Middle East (Egypt, Turkey), the Indian subcontinent with Nepal, and South East Asia, including Indonesia (Almeria *et al*., 2019).

During the 1990s, considers in unprotected populaces in endemic territories demonstrated pervasiveness levels in those specific gatherings around or higher than 10%. For instance, 11.2% of 964 outsiders in Nepal (Hoge *et al.*, 1993); 11% of 450 HIV-tainted patients in Haiti (Pape, 1994), 12.4% of 459 outside inhabitants in (Fryauff *et al.*, 1999), and 18% of 144 in youngsters in Peru were contaminated. During a similar period, commonness went from 0.3 to 0.5% in the USA (Wurtz, 1994) also, in the UK, where an aggregate of 6151 stools was tried from 5374 patients and only7 stools tests from 4 patients (0.1%) were certain (Clarke & Mcintyre, 2020).

In 2010, a survey of past investigations on endemic regions from 22 nations (Mexico, Guatemala, Honduras, Brazil, Peru, Venezuela, Cuba, Turkey, Jordan, Saudi Arabia, China, Nepal, Bangladesh, Lao PDR, Thailand, Indonesia, Egypt, Nigeria, Uganda, Kenya, Tanzania, and Mozambique) was distributed (Chacín-Bonilla, 2010). This survey detailed that the disease rates in those nations up to 2010 went from 0% to 13% (normal 1.7%) in 47,642 immunocompetent people, most with looseness of the bowels, from endemic

territories going to social insurance habitats. In light of a similar survey, rates from coordinated asymptomatic controls differed from 0% to 4.2% (normal 0.4%) (Fletcher *et al.*, 2011).

In a similar Metadata investigation, higher commonness rates were seen in immunocompromised people; among 3340 immunocompromised people, for the most part, HIV/AIDS patients with Diarrhea, predominance ran from 0% to 36% (average 4.5%) (Chacín Bonilla, 2010). The most elevated predominance was seen in Peru (41.6%) Burstein Alva, S. (2005). Another deliberate audit of 27 examinations from 14 sub-Saharan nations, uncovered a general pervasiveness of 18% (Fletcher *et al.*, 2011).

Over 11,500 cases have been accounted for to date in North America (Canada and USA). Of those, just in the USA over the most recent four years (2016–2019) have there been very nearly 4500 cases and checking, since the outbreak season is not finished for the current year. Excluding North America, more than 2500 cases have been reported (Almeria *et al.*, 2019). In Colombia, Saudi Arabia, Malaysia, Tanzania, and Cameroon, infection rates of 2.6%, 5.9%, 4.9%, 1.2%, and 3.6%, respectively, have been found (Nsagha *et al.*, 2016).

Considering that many countries do not include the parasite in their diagnostic protocols, these numbers are an underestimate of the real cases related to *Cyclospora cayetanensis* infection. Most outbreaks due to *Cyclospora cayetanensis* described to date have been related to fresh produce consumption. *Cyclospora cayetanensis* outbreaks have been mostly reported in North America, probably due to better detection methods and disease surveillance that have helped in tracking outbreaks (Almeria *et al.*, 2019).

Variations in prevalence of infection may be influenced by study design, geographic area, age, and immunologic status of the population studied, seasonal variability of the parasite, methods of detection used, and expertise of the microscopes (Chacin-Bonilla, 2010).

2.5.2.3 Life Cycle and Transmission

The life cycle of *Cyclospora cayetanensis*: The oocysts of *Cyclospora cayetanensis* are spherical, measuring 8-10μm in diameter and the mature oocyst contains two sporocysts. When freshly passed in stools, the oocyst is not infective (thus, direct fecal-oral transmission cannot occur; this differentiates *Cyclospora* from other important coccidian parasites) (CDC, 2020).

In the environment, sporulation occurs after days or weeks at temperatures between 22°C to 32°C, resulting in a division of the sporont into two sporocysts, each containing two elongate sporozoites. Fresh produce and water can serve as vehicles for transmission and the sporulated oocysts are ingested (in contaminated food or water). Washing does not easily remove oocysts from fruits and vegetables. The infectious dose is unknown but is presumed to below. Animal reservoirs have not been found (Klassen-fischer *et al*., 2008).

The oocysts excyst in the gastrointestinal tract, release the sporozoites, which invade the epithelial cells of the small intestine. Inside the cells, they undergo asexual multiplication and sexual development to mature into oocysts, which will be shed in stools (CDC, 2020). Figure four below shows the life cycle of this parasitic organism.

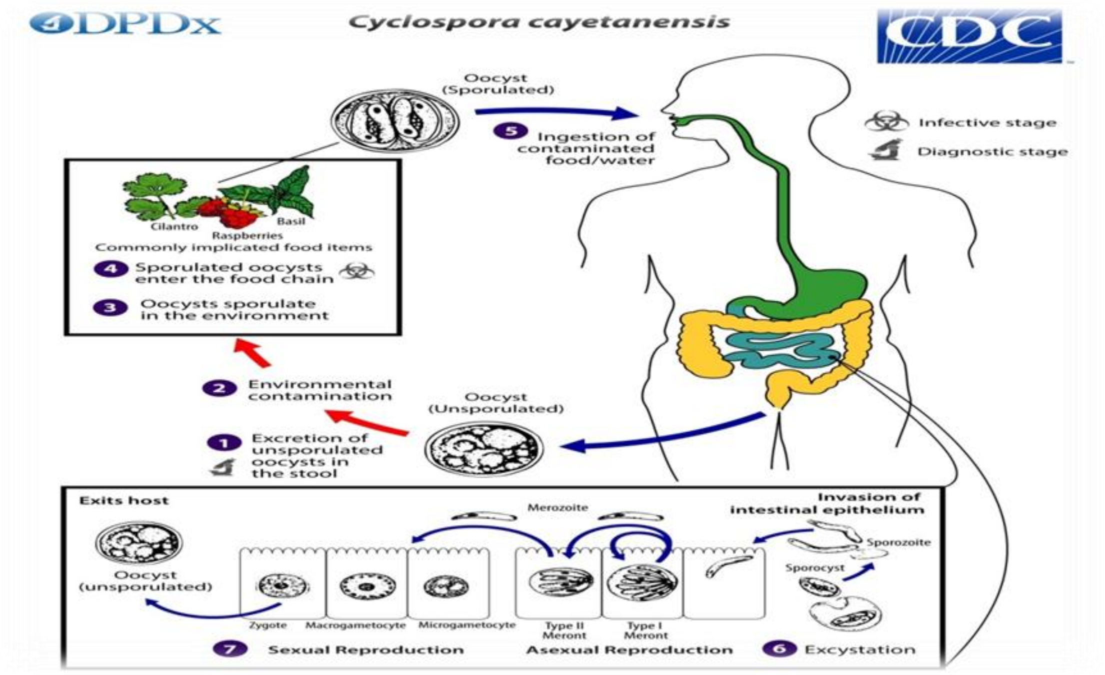

Figure 3: The Life cycle of *Cyclospora cayetanensis*: Source: (CDC, 2019)

2.5.2.4 Clinical Symptoms and Pathogenesis

The pathogenesis fundamental these side effects have not been characterized.
Jejunal biopsies have indicated mucosal adjustments in intestinal villi, diffuse edema, and invasion by incendiary cells, receptive hyperemia, vascular widening, and clog of vessels within the sight of the parasite, which is perfect with irritation of the upper intestinal tract (Ortega *et al*., 1997).

The components of *Cyclospora cayentanensis* pathogenesis are not completely comprehended. Concerning Giardia and *Cryptosporidium*, it is accepted that the life forms are profoundly irresistible and that dosages lower than 100 sporulated oocysts convey a high likelihood of disease. Like other known types of *Cyclospora*, the fundamental site of contamination of *Cyclospora cayentanensis* is the small digestive system (Almeria *et al.,* 2019).

The parasite's particular site is accepted to be, where it meddles with assimilation. Investigations of the gastrointestinal district of tainted patients have uncovered aggravation of the lamina propria, surface epithelial confusion, villous decay, and blunting, and crypt hyperplasia of jejunal tissue. Sexual and biogenetic type of *Cyclospora* has been found in the cytoplasm of enterocytes (intestinal epithelial cells) even though it is as of now obscure whether the pathogenesis of this parasite is expected to enterocyte brokenness or poisons discharged straightforwardly by the parasite. Ongoing examinations have demonstrated that there is an astounding measure of hereditary assorted variety among certain hereditary successions. This proposes the plausibility of polyparasitism, concurrent disease with various strains of the parasite (Almeria *et al.,* 2019).

The incubation dated of *Cyclospora cayetanesis* is 2–22 days. Symptoms include watery diarrhea, abdominal cramping, nausea, anorexia, fatigue, and weight loss. In HIV-positive patients C. *cayetanesis* produces prolonged diarrhea, wasting, and weight loss (Ortega *et al*., 2020).

2.5.2.5 Diagnosis

After formal-ether fixation, circular oocysts 8-10 µm in distance across is seen. Under ultra-violet brightening, the oocyst divider shows brilliant blue fluorescence.

With adjusted Ziehl-Neelsen recoloring, as utilized for diagnosing of *C.caytenensis*, the oocysts recolor corrosive quick. In any case, some don't take up the stain at all and rather show up as shiny, wrinkled circles. Size is a significant rule in the identification of any protozoan blister and estimation of the oocyst will assist with building up the right finding (Almeria *et al.,* 2019). Oocyst of *Cyclospora cayetanensis,* are twice as large in comparison with *C. parvum* and are not sporulated (do not contain sporocysts upon excretion).

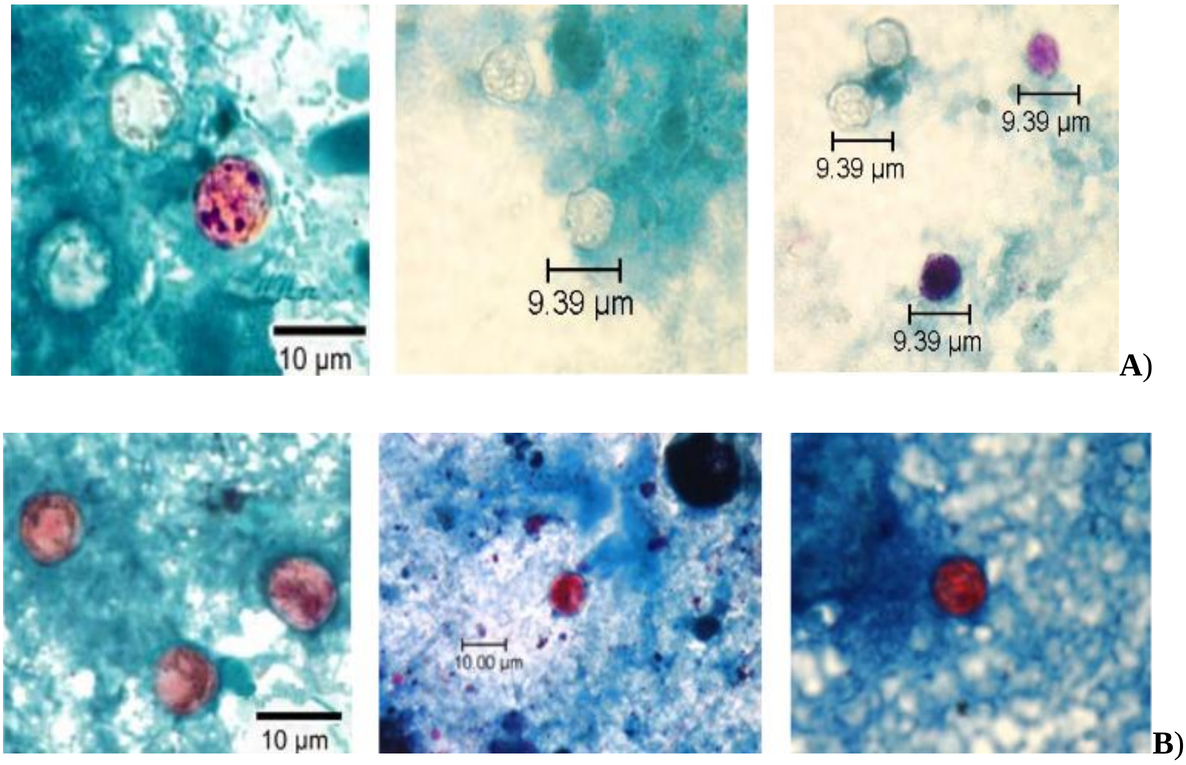

Figure 4 *Cyclospora* oocysts A)**.** *Cyclospora cayetanensis* oocysts stained with modified acid-fast technique (CDC,2020). B). *Cyclospora cayetanensis* oocysts stained with the safranin stain technique (CDC, 2020).

2.5.2.6 Prevention

Avoid food or water that may have been polluted with stool. When traveling, use safe food and water habits. Available from: www.cdc.gov/parasites/cyclosporiasis.

2.5.2.7 Treatments

Treatment consists of supportive care, maintenance of fluid and electrolyte balance, symptomatic relief and antibiotic therapy. Cotrimoxazole (Trimethoprim-sulfamethoxazole) effectively eradicates the organism (Klassen-fischer *et al*., 2008; Gruenberg and Guglielmo, 2019).

2.5.3 Cystoisosporiasis (Formerly Isosporiasis)

Cystoisosporaisis (formerly known as isosporiasis) is an intestinal disease of human-caused by the coccidian parasite *Cystoisospora belli* formerly *Isopora bell* (CDC, 2020).

2.5.3.1 Morphologic Description

Isospora oocysts are ellipsoid and approximately 20 µm to 33 µm by 10 µm to 19 µm. The smooth, thin cyst wall is composed of 2 layers. The outer layer is tough, impermeable to fluids, and environmentally resistant; the inner layer is membranous. Sporocysts are 12 µm by 7 and µm to 9 µm. Sporozoites are slender and crescent-shaped (Klassen-fischer *et al.*, 2008).

The internal structures of sporozoites and merozoites are similar to those of other coccidian and include polar rings, rhoptries, micronemes, conoids, microtubules, and amylopectin granules. Trophozoites are spherical. Macrogamonts contain a single large, centrally located nucleus and wall-forming bodies. Microgamonts contain multiple nuclei that migrate to the periphery, elongate and protrude from the surface, and bud into mature flagellated microgametes 5 µm to 6 µm long (Klassen-fischer *et al*., 2008).

2.5.3.2 Epidemiology

An irresistible illness that influences people and creatures, and it is brought about by the parasites *Isospora hominis, Isospora natalensis* and *Cystoisospora belli* . The sort *Isospora* has a place with the family *Eimeridae*, of the suborder *Eimeriina*, and is a piece of the subclass *Coccidia* (Sporozoa class), alongside the variety Sarcocystis, *Toxoplasma, Cryptosporidium* and *Cyclospora*. Around 200 types of *Isospora* have been depicted; nonetheless, obviously, just a single animal category (*Cystoisospora belli* , otherwise called *Cystoisospora belli*), taints people (Oddó *et al.*, 2018).

Before the rise of AIDS, Isosporiasis was fundamentally viewed as a tropical or sub-tropical disease-endemic of immature nations, or an ailment of explorers (Klassen-fischer *et al.*, 2008). In, cases showed in America, Europe, Africa, Asia, and Australia have been related (AIDS) (Michiels *et al*., 1994).

One investigation discovered positive assessment discoveries in up to 15% of Haitians tainted with AIDS. In creating nations, 8-40% of patients with AIDS are tainted. Cystoisosporiasis is the underlying AIDS-characterizing ailment in around 2-3% of patients with AIDS who are from Africa. Among patients with AIDS who are from South America, 10% with incessant Diarrhea have isosporiasis. In patients with AIDS who are from Haiti and Africa, 7-20% with

nonstop looseness of the bowels has cystoisosporiasis (Rodríguez-Pérez *et al*., 2019; Rodriguez-Morales & Castañeda-Hernández, 2019).

In the United States of America, Cystoisosporiasis is increasingly normal in people with AIDS, 0.2-3% of who have stools constructive for *Cystoisospora belli* (*C.belli*). In any case, this expanded commonness has been diminished by the across the board utilization of Pneumocystis jiroveci pneumonia (PCP) prophylaxis with trimethoprim-sulfamethoxazole (TMP-SMZ) among patients with HIV disease (Velasco-Hernandez *et al*., 2002).

Even though *Isospora* (*Cystoisospora*) *belli* finishes its life cycle in people, the oocysts shed in the defecation of contaminated people must develop (sporulate) outside the host, in the earth, to get infective. Based on restricted information, the development procedure is finished in around 1 to 2 days; however, it may happen all the more quickly in certain settings. The disease results from the ingestion of sporulated oocysts, for example, from defiled nourishment or water. After ingestion, the parasite attacks enterocytes in the small digestive system. Eventually, youthful oocysts are delivered and shed in stool (Certad *et al., 2003)*. .

Individuals of any age are vulnerable to *C.belli* disease, although it will, in general, be progressively genuine in newborn children and little youngsters, perhaps because of the danger of lack of hydration right now. *C.belli* can cause extreme Diarrhea in newborn children. No sex inclination for contamination has been noted, besides the sexual orientation conveyance of individuals with AIDS, the hazard factor most normally connected with this ailment (Certad *et al., 2003)*.

2.5.3.3 Life Cycle

Cystoisospora (Isospora) *belli* completes its life cycle in humans as illustrated in figure two. The oocysts shed in the feces of infected individuals must mature (sporulate) outside the host, in the environment, to become infective. The maturation process is completed in approximately 1 to 2 days but might occur more rapidly in some settings. Infections result from the ingestion of sporulated oocysts, such as from contaminated food or water. After ingestion, the parasite invades enterocytes in the small intestine. Ultimately, immature oocysts are produced and shed in the stool (Lindsay *et al*., 1997).

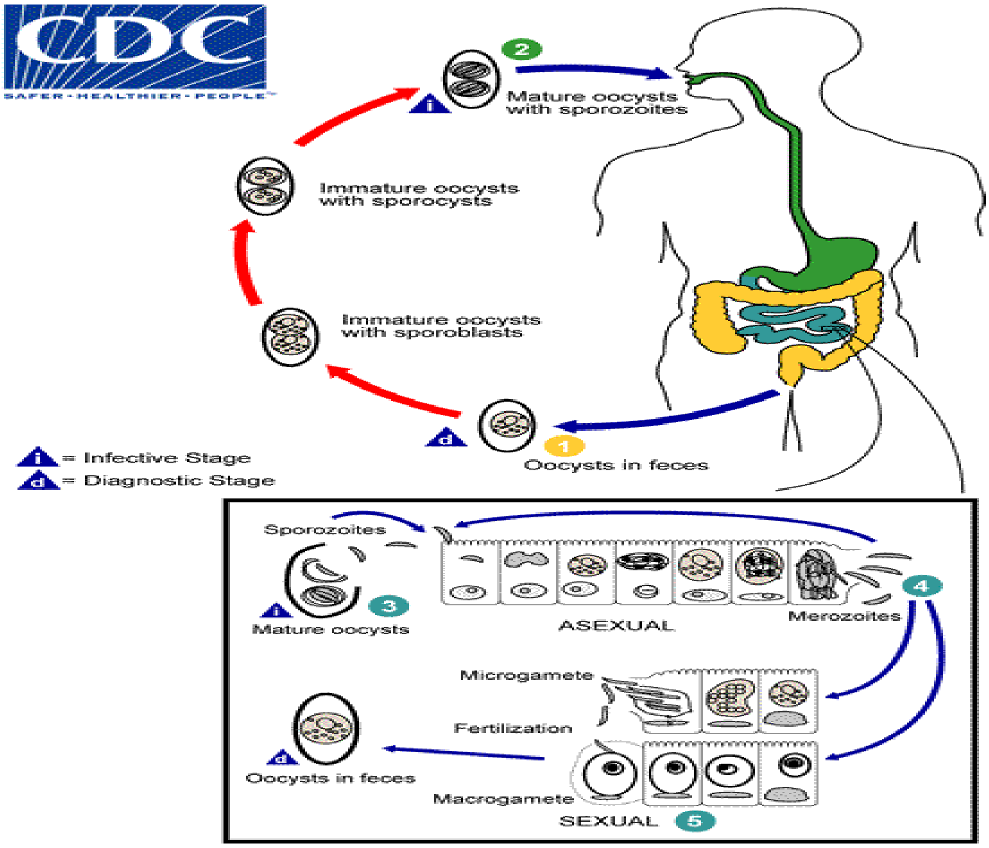

Figure 5: The Life cycle of *Cystoisospora (Isospora) belli:* Source*:* (CDC, 2019**)**

2.5.3.4 Pathogenesis

C.belli contamination is most regularly seen in immune-compromised people or in people who have as of late made a trip to tropical territories, in individuals who are systematized, or in people who live in poor clean conditions. The incubating time frame ranges from 3 to 14 days. Side effects start the roughly multi-week after ingestion of the oocysts and last 2-3 weeks, with slow improvement. Contamination in immune-compromised individuals may proceed uncertainly.

C.belli can cause stamped villous decay and grave hyperplasia in the small digestive system. Provoking invades in the lamina propria incorporate eosinophils, neutrophils, lymphocytes and plasma cells. The exact system causing these progressions is obscure; however, they bring about steatorrhea and malabsorption. The disease of the biliary tract by *C. belli* is likewise conceivable. The parasite can finish its life cycle in the biliary tract and oocysts can be seen in bile. Stages are situated in the bile conduit epithelium.

Extreme drying out is the most well-known complexity and quite often happens in patients who are youthful or immunocompromised. Acalculous and cholecystitis have been accounted for in patients with AIDS. Tissue attack and scattering have been accounted for on dissection discoveries in a couple of patients with AIDS. Colitis in patients with AIDS has been occasionally announced. Receptive joint pain is uncommon yet has been accounted for in immunocompromised patients.

2.5.3.5 Clinical Manifestations

The most common manifestation is watery, non-bloody diarrhea, which can be related to abdominal pain, cramping, anorexia, nausea, vomiting, and low-grade fever (Dehovitz *et al*., 1986). The diarrhea is profuse and prolonged, particularly in immunocompromised patients, leading to severe dehydration, electrolyte abnormalities like hypokalemia, weight loss, and malabsorption. Acalculous cholecystitis/cholangiopathy (Walther & Topazian, 2009) and reactive arthritis (Oddó *et al*., 2018) have been reported. Myalgias and Headache in rare case otherwise, minimal abdominal tenderness could also be present (DeHovitz *et al*., 1986).

2.5.3.6 Diagnosis

Typically, infection is diagnosed by detecting *Isospora* oocysts (dimensions, 23–36 µm by 12–17 µm) in fecal specimens (Lindsay *et al.,* 1997). Oocysts could also be shed intermittently and at low levels, even by patients with profuse diarrhea. Diagnosis is facilitated by repeated stool examinations with sensitive methods, like modified acid-fast techniques, on which oocysts stain bright red, and UV microscopy, under which they autofluorescence of *I. belli* oocyst walls have been reported and may be utilized in diagnosis. Autofluorescence was superior to iodine staining in one study (Guiguet *et al*., 2007) and (Knobloch *et al*., 2002). A real-time PCR assay has been developed to detect *I. belli* in stool samples (Tan *et al*., 2008). Infection can also be diagnosed by detecting oocysts in duodenal aspirates/mucus or developmental stages of the parasite in intestinal biopsy specimens (Lindsay *et al*., 1997). Extra intestinal infection, like within the biliary tract, lymph nodes, spleen, and liver, has been documented in postmortem examinations of HIV- infected patients (Lindsay *et a*l., 1997).

A) 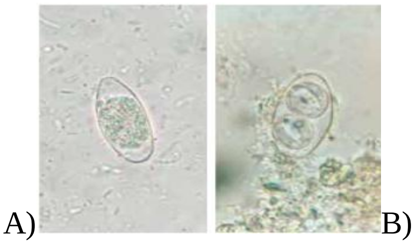 B) 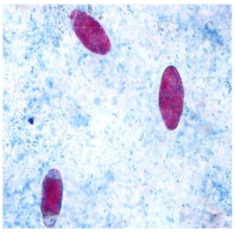 C) 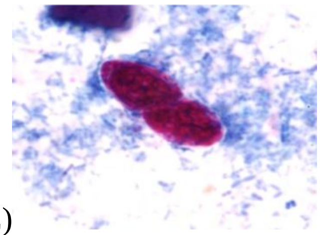

Figure 6. Oocyst of *Cystoisospora belli*

A) *Cystoisospora belli* Oocysts left: unpopulated; right: sporulated with 4 sporozoites in each of the two sporocysts. Unstained Size: 20-33 µm by 10-19 µm (WHO, 2019). Oocyst of *Cystoisospora belli* stained with modified Ziehl Neelsen magnification (1000X). B) Unsporulated oocyst C) Sporulated oocyst (Endeshaw Tokola, 2005)

2.5.3.7 Preventing Exposure

Because *C.belli* acquired by ingesting infected water or food, avoiding potentially contaminated food or water in isosporiasis endemic areas may help prevent infection (National Institutes of Health, 2019).

2.5.3.8 Preventing Disease

In some settings, chemoprophylaxis with trimethoprim-sulfamethoxazole (TMP-SMX) has been related to a lower incidence or prevalence of isosporiasis (Anglaret *et al*., 1999). In an exceedingly randomized, placebo-controlled trial, daily TMP-SMX (160/800 mg) was protective against isosporiasis in persons with early-stage HIV infection World Health Organization clinical stage two or three at enrollment) (Anglaret *et al*., 1999; Wiwanitkit, 2001).

2.5.3.9 Treating Disease

Clinical management includes fluid and electrolyte support for dehydrated patients and nutritional supplementation for malnourished patients. TMP-SMX is the antimicrobial agent of choice for the treatment of isosporiasis. It's the sole agent whose use is supported by substantial published data and clinical experience. Therefore, potential alternative therapies should be reserved for patients with documented sulfa intolerance or in whom treatment fails. Three studies in HIV -infected patients in Haiti have demonstrated the effectiveness of assorted (Siberry *et al*., 2013; National Institutes of Health, 2019; CDC, 2020).

2.5.4 Blastocytosis

Blastocytosis is an intestinal tract illness caused by the microscopic parasite *Balstocystis* species. *Balstocystis* species is a common microscopic unicellular protozoan and one of the most unusual parasites found worldwide. This parasite firstly described in the medical literature with the aid of Alexeieff and become considered as innocent yeast at that time. But, *B. hominis* is now getting recognition as an agent of the human intestinal complaint (Doherty, 2002; Abdel-hafeez *et al*., 2012).

2.5.4.1 Epidemiology

The pathogenic potential of *Blastocystis* remains controversial, however >1 billion individuals worldwide are colonized by *Blastocystis*, asymptomatic colonization is common and pathogenicity has yet to be robustly demonstrated in vivo.

B. hominis is a eukaryotic, cosmopolitan parasite, infecting various animals ranging from birds, pigs, horses, amphibians, reptiles, to insects. The prevalence of *B. hominis* is 1.5-10% in developed countries, where it can cause deterioration of health in immune-compromised patients. It can be harmful to HIV-positive patients or organ-transplant patients who receive immunosuppressive agents (laksemi *et al,* 2020). On the other hand, the prevalence of *B.hominis* infection was 72% in people with HIV/AIDS in Indonesia (Sadaf *et al*., 2013). Research in the United States and Paris showed that *B. hominis* infections were more frequent compared to *Entamoeba histolytica*, *G. lamblia*, and *C. parvum* in HIV/AIDS patients (Wawrzyniak *et al., 2013)*.

B. hominis in stool samples of symptomatic and asymptomatic individuals become evaluated as a likely reason for gastrointestinal troubles. As well as *B. hominis* is common in agents of travellers' diarrhea, it may purpose chronic or recurrent diarrhea in patients with AIDS and other immunodeficiencies. *B.hominis* changed taken into consideration to be a member of normal intestinal flora within the beyond, but in latest years it's been widely wide-spread as a very debatable pathogenic protozoan (Clark *et al*., 2013); Scanlan, 2012).

It is also said that it could be seen in nosocomial diarrhea instances. Contamination with *B.hominis* has a worldwide distribution and occurs in each youngster and adults. The occurrence of *B. hominis* in distinct areas is said to be among 2-65%. It has been reported that whilst *B. hominis* is being detected in 15-20% of acute gastroenteritis instances with the

direct microscopic investigation, detection feces can reach 65% with trichrome stain.

2.5.4.2 Morphology:

Blastocysts are a polymorphic protozoan with four morphological variants.

Vacuolar form: spherical, with a big vacuole centrally positioned that takes up about 90% of the cell. The cytoplasm and its contents are squeezed to a thin peripheral edge. Nuclei and enclosure bodies are observable in the thickest portion of the cytoplasm. The vacuole may be empty or contain thin or wool-like material. This form most commonly found in stool specimens available from http://www.atlas-protozoa.com/index.php.

Granular form: like to the vacuolar form, but with granulations in the cytoplasm or, more commonly, in the central vacuole (Tan, 2008).

Amoeboid form: It is infrequently stated, due to the heterogeneous descriptions of its morphology. This form is rarely seen in vitro cultures but has also been reported in dysenteric stool samplings (Tan & Suresh, 2006).

Cystic form: Due to its small size (2-5µm), it may be overlooked during a microscopic examination; it is a rare finding in cultures too. The cyst is round or oval, with a double wall. The cytoplasm contains 1 to 4 nuclei. Different ways of reproduction for *Blastocyst* have been hypothesized and so far, binary fission seems the most likely. When cysts are ingested they excyst in the large intestine and take on either the vacuolar, granular or the amoeboid form. The latter encyst during their passage through the large intestine and are eliminating in feces. http://www.atlas-protozoa.com/index.php.

2.5.4.3 Life Cycle and transmissions

The life cycle of *Blastocyst* species is not but understood, consisting of the infectious stage and whether or not (and which of the) diverse morphologic varieties of this polymorphic organism that have been recognized in stool or lifestyle represent distinct biologic stages of the parasite within the intestinal tract of hosts. The cyst form (3–5 µm) is postulated to be an infectious stage, but no longer showed (Clinical Reevaluation, 2007; Doyle *et al.,* 1990; Stenzel & Boreham, 1996). The foremost form observed in human stool specimens is referred to as the vacuolar (important body) shape and is of variable size (5-40 µm, now and then a good deal larger).

Replication seems to arise through binary fission. Other morphologic paperwork (e.g. ameboid and granular paperwork) also were referred to in stool samples and/or culture; their biological function and eventual developmental fate require similar investigation (Tan, 2008).

Blastocystis hominis is transmitted through fecal-oral infection, in a way just like different gastrointestinal protozoa. There are collecting reports that Blastocystis is related to intestinal disorders in people immunocompromised by HI or immunosuppressive remedy (Hailemariam Getachew *et al*., 2004) suggesting that Blastocystis is an opportunistic pathogen. The parasite is also common among food and animal handlers (Danchaivijitr *et al*., 2005).

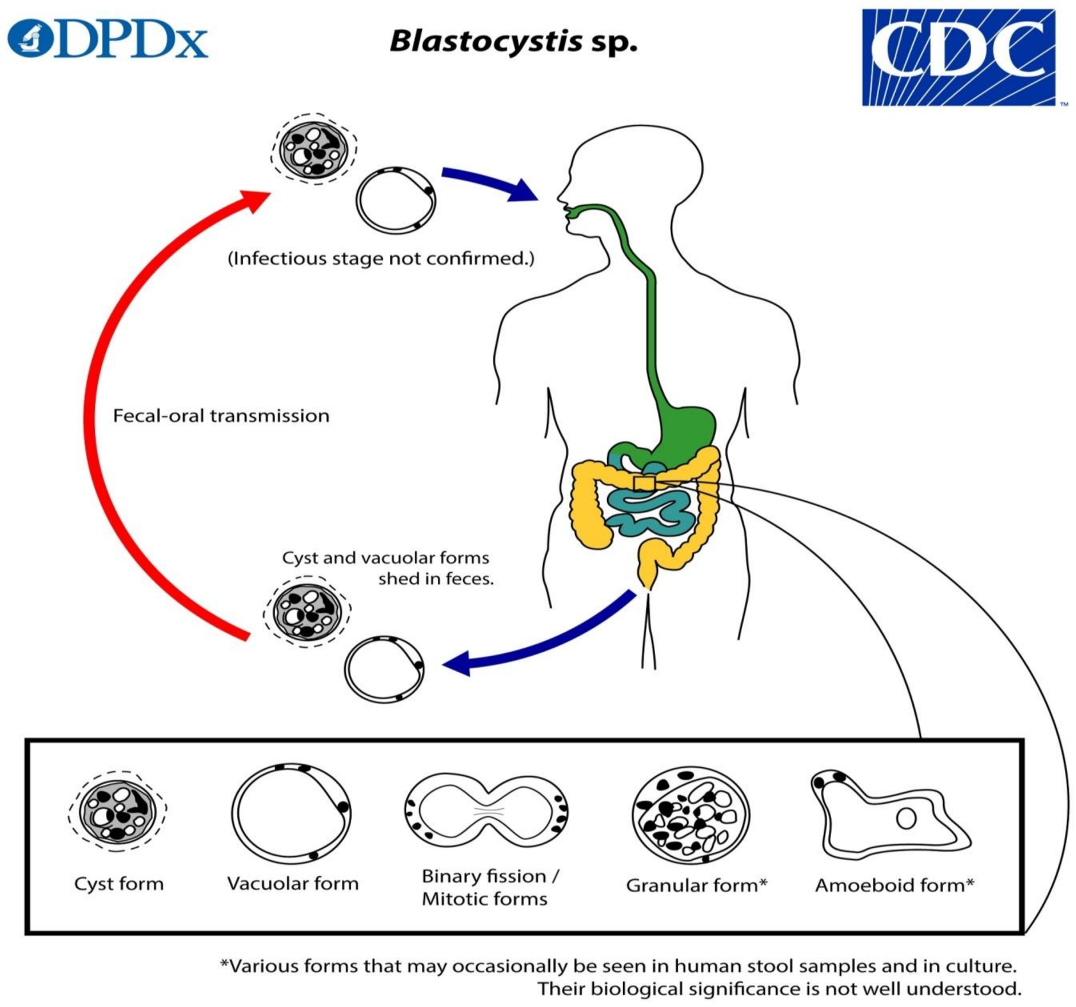

Figure 7: The Life cycle of *Blastocystis* species Source: (CDC, 2019)

2.5.4.4 Pathogenesis and Clinical Manifestations

Blastocystis hominis is an intestinal parasite that has been now not recognized as such as much as the recent beyond are emerging and increasing nowadays. The hassle has ended up greater extreme with the onset of the HIV/AIDS pandemic. Among those, the status of *Blastocystis hominis* as a cause of diarrhea is a debatable and no longer well-documented one (Jelinek *et al*., 1997).

The protozoa are commonly found in individual feces without any symptoms, which show this parasite is of low pathogenicity (laksemi *et al,* 2020). Reviews of asymptomatic and symptomatic *B. hominis* infections in people are international. *B.hominis* infections are predominantly stated from developing international locations of tropical and subtropical areas. Well identified medical signs and symptoms because of *B. hominis* in symptomatic people consist of stomach pain or pain, diarrhea, nausea, vomiting, flatulence, gastroenteritis, colitis, and other minor complaints (Stenzel & Boreham, 1996; laksemi *et al,* 2020).

2.5.4.5 Diagnosis of Blastocytis

Blastocystis poses great demanding situations to the diagnostic laboratory, for many reasons. The unsure pathogenesis of the protozoan does no longer inspire microbiologists to search for the organism in specimens. The organism may be instead nondescript even in stained arrangements and can be confused as yeast, *Cyclospora*, or fat globules. The pleomorphic nature of the parasite complicates identity. Lastly, the fecal cyst may predominate in cultures but are extremely tough to perceive without awareness methods due to their small size (3-5µm) (Sadaf *et al*., 2013; Wawrzyniak *et al*., 2013 & Chen *et al.,* 2014).

Blastocystis is historically recognized utilizing searching out vacuolar paperwork in direct stool specimens (Andiran *et al*., 2006). Even though this approach is rather insensitive (Termmathurapoj *et al*., 2004). Present-day diagnostic laboratories need to additionally consist of fecal cysts as an indicator of infections.

Those may be selectively concentrated by density-gradient tactics to increase sensitivity. In vitro cultures of stool specimens had been six and times greater sensitive while compared to easy smears and trichrome staining respectively (Termmathurapoj *et al*., 2004). However, the same have a look at revealed that the culture technique did fail to discover a few parasites that easy smears and trichrome staining did, indicating that no longer all Blastocystis isolates

can be effortlessly cultured in vitro (Sadaf *et al*., 2013; Wawrzyniak *et al*., 2013 & Chen *et al.*, 2014).

2.5.4.6 Prevention

The prevention mechanism of *Blastocystis* is to just like different gastrointestinal protozoa parasites (Sadaf *et al*., 2013; Wawrzyniak *et al*., 2013 & Chen *et al.*, 2014).

2.5.4.7 Treatment

They want to deal with individuals infected with *Blastocystis* has been equivocal, because of the uncertain pathogenesis of the organism and the remark that the ailment is frequently moderate and self-limiting. In cases in which remedy is warranted, at present, the primary desire of the chemotherapeutic agent metronidazole (Flagyl) is the most normally prescribed antibiotic as defined inside the literature (Moghaddam *et al*., 2005; Laksemi *et al*, 2020).

Various drug regimens for metronidazole had been prescribed ranging from 250–750 mg three times a day for 10 days 1.5 mg an afternoon for 10 days (Cassano *et al*., 2005). Metronidazole may be utilized in combination with other pills inclusive of paromomycin or co-trimoxazole (Trimethoprim/ Sulfamethoxazole) (Andiran *et al.*, 2006; Sadaf *et al*., 2013; Chen *et al.,* 2014; Wawrzyniak *et al*., 2013).

2.5.5 Microsporidiosis

Microsporidiosis is intestinal infection caused by *Microsporidia* species. *Microsporidia* is a minute intracellular obligate organism labelled as eukaryotes because it has membranes, intra-cytoplasmic membrane systems, and chromosome separation in mitotic-spindles, and is closest to fungus because it consists of chitin at the spore wall, however, is extra discussed as protozoa. *Microsporidia* has small, resistant spores; the phylum consists of 187 genera and 1500 species. Available from: (https://www.ncbi.nlm.nih.gov/books/NBK537166/).

The various almost 1500 species described, best 17 are pathogenic to humans, and some of them include *Enterocytozoon bieneusi, Encephalitozoon, Anncaliia, Enterocytozoon, Tubulinosema, Microsporidium African,* and *Trachipleistophora hominis*. In most people of cases, *E. bieneusi* and *Encephalitozoon intestinalis* have been the two species detected most usually in infected people (lobo *et al.*, 2012).

In evolved nations, the prevalence prices for Microsporidia contamination in HIV-seropositive people with diarrhea variety from 2% to 78% various via the degree of immunosuppression and treatment. In HIV-seropositive folks without diarrhea, infections range from 1.4 % to 4.3%. In individuals no longer infected with HIV, the seroprevalence rates range from 1.3% to 22% among blood donors, pregnant women, slaughterhouse employees, and humans with unknown causes of diarrhea likely as a result of Microsporidia contamination (Rodríguez-Pérez *et al.*, 2019).

2.5.5.1 Epidemiology

In America presently, most instances of microsporidiosis are mentioned in immunosuppressed adults, especially people with HIV-related immunosuppression. Research has located that *E. bieneusi* contamination of small intestinal enterocytes is detected in 15-34% of patients with AIDS with continual diarrhea and no different identified reasons.

International Microsporidia has a global distribution. Instances of Microsporidiosis had been mentioned in each developed and developing countries and among both immunosuppressed and immunocompetent people. Microsporidiosis has been mentioned in the Americas, Asia, Europe, and Africa (Ghoyounchi *et al.,* 2017; Shadduck & Pathobiology, 2019).

Maximum instances of intestinal and disseminated microsporidiosis in sufferers with HIV are pronounced in folks that are seriously immunocompromised ($CD4^{+}$< one hundred/µL); in those patients, morbidity can be huge. *E.bieneusi* infections often bring about protracted debilitating contamination with diarrhea, which may also remain for several months. *E.bieneusi* infections deliver a mortality of up to 56%. In addition to persistent diarrhea, malabsorption and losing can arise in men and women with AIDS. *E.bieneusi* is answerable for extra than 19% of intestinal microsporidiosis cases in this population; *E intestinalis* money owed for the remainder (Ghoyounchi *et al.*, 2017; Shadduck & Pathobiology, 2019)

Reviews of *E. bieneusi* infections are increasing amongst tourists and residents of tropical international locations who do not have HIV infection. *E. intestinalis* infection associated with continual diarrhea has been said in immunocompetent travelers. Microsporidiosis has no recognized gender, age, and racial predilection (Rodríguez-Pérez *et al.*, 2019; Anane & Attouchi, 2010).

2.5.5.2 Pathogenesis

Humans collect Microsporidiosis through the ingestion or inhalation of Microsporidia spores. Research has reported Encephalitozoon species with in the urinary tract in people with disseminated infections, suggesting that sexual transmission is viable. The spore is the infective form. Spores are environmentally resistant and are surrounded by using an outer electron-dense glycoprotein layer and an electron-lucent endospore layer composed in the main of chitin (Nsagha *et al*., 2016).

The spore extrudes its polar tubule and injects the infective sporoplasm into the host mobile. As soon as inside the cellular, it multiplies utilizing binary fission or schizogony. Improvement can occur immediately inside the host cellular cytoplasm or interior parasitophorous vacuoles. As mature spores increase and gather, the cellular expands, and in the end, ruptures, liberating the spores (Nsagha *et al*., 2016).

E.bieneusi contamination has additionally been found in renal transplant recipients with persistent weight reduction and diarrhea. A case of fatal myositis due to *B. algerae* contamination has been documented in a female with diabetes and rheumatoid arthritis who have been prescribed infliximab. *E.bieneusi* is normally determined most effective in enterocytes. *E.intestinalis* is extra (Nsagha *et al*., 2016). Invasive and produces disseminated disorder involving the small and big intestines, gallbladder, urinary tract epithelium, and respiration tract epithelium. Biliary tract involvement that progresses to cholangitis and cholecystitis is common in sufferers with AIDS who have *E. bieneusi* infection. Myositis due to Pleistophora infection has been documented (Nsagha *et al*., 2016).

2.5.5.3 Life cycle and transmission

The life cycle of Microsporidia includes three levels, termed infective, proliferative, and sporogony (Rodríguez-Pérez *et al*., 2019; Anane & Attouchi, 2010). Microsporidia can be transmitted through urine, soil, water, and food. Microsporidia contamination might also affect muscle mass, intestines, gall bladder, liver, kidneys, eyes, mind, lungs, skin, and nasal sinuses. Bowel Microsporidia maximum commonly arises in 30–50% of AIDS sufferers with chronic diarrhea (Rodríguez-Pérez *et al*., 2019; Anane & Attouchi, 2010).

Persons cause Microsporidial infections or animals infected with Microsporidia are feasible sources of infections and man or woman-to-character transmission via fecal-oral route may

be sizeable. The existence cycle includes repeated divisions of binary fission (merogony) or a couple of fission (schizogony) and spore manufacturing, sporogony. Both merogony and sporogony can arise inside the identical cellular at the same time (Gracia and Bruckner, 1997).

In appropriate host cells, the sporoblasts which can be launched from the spores come to be meronts after which meronts develop into sporonts, that is characterized by using a dense surface coat (Franzen & Müller, 2001). This surface coat later develops into the exospore layer of the spore wall and provides environmental safety for this infectious degree of the parasite. Sporont multiplies by way of binary fissions and divides into sporoblasts to change into mature spores. The figure below shows the life cycle of the Microsporidia species.

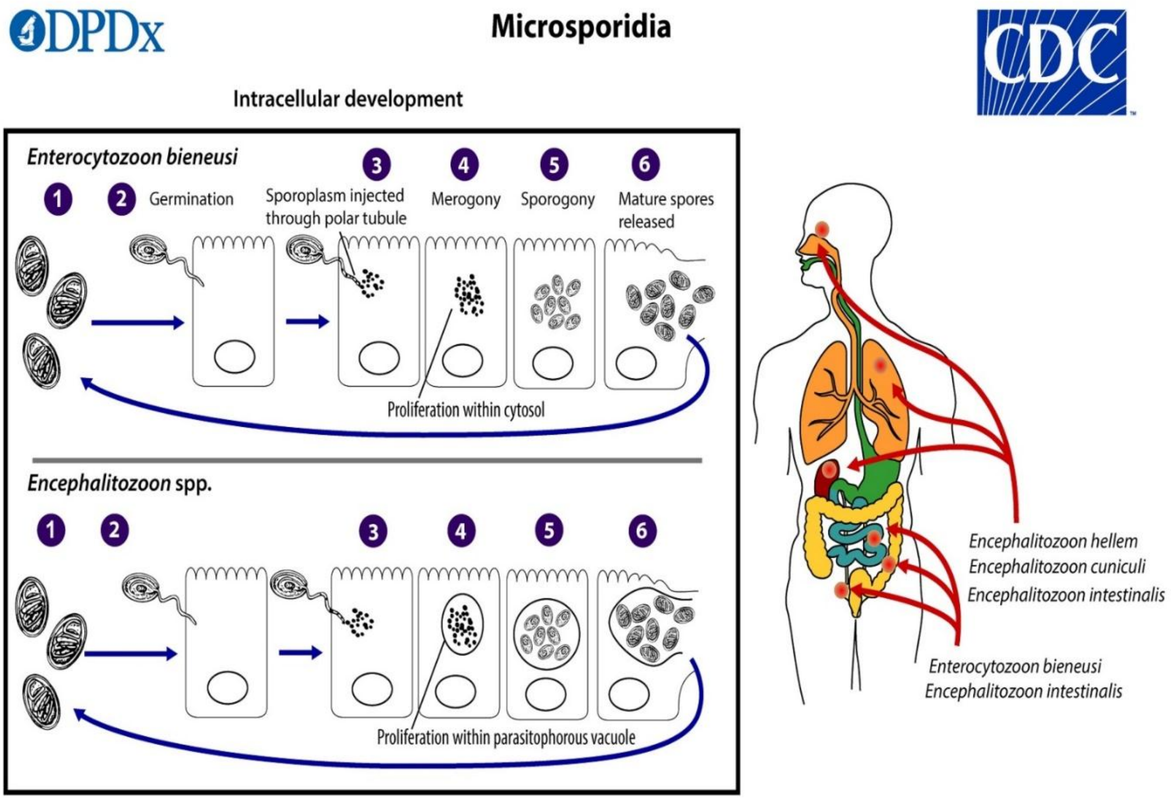

Figure 8: The Life cycle of *Microsporidia* species Source: (CDC, 2019)

2.5.5.4 Diagnosis

Microsporidia are small (1.5-2.5 µm x 2.5-four µm) oval obligate intracellular eukaryotic protozoan parasites that are living and multiply in the intestine of host cells and are diagnosed in human infections (Shadduck & Pathobiology, 2019). Analysis of Microsporidia is decided

by using the use of numerous strategies such as Giemsa, Gram, and or Ziehl–Neelsen staining, to discover spores containing polar filaments. contact preps and smears from biopsy, eye scraping, tissue specimens, and aspirations stained with Gram, Giemsa, or Trichrome can be identified in urine, stool, and duodenal fluid sample(Ghoyounchi *et al.*, 2017; Shadduck & Pathobiology, 2019).

There are versions in Microsporidia, which help become aware of genus and species; they consist of the wide variety of spores produced in sporogony, the manner spores are produced, host interactions with parasites, morphological traits of developmental degrees, nucleation, the region where contamination occurs, and serological and molecular analysis (Ghoyounchi *et al.*, 2017; Shadduck & Pathobiology, 2019). Molecular examination strategies for diagnosing Microsporidia encompass fluorescence, electron microscopy, ELISA, and Western blotting. Immunofluorescence tests the use of the chitin-binding Fluorochrome Uvitex 2B, Fungifluor, Calcofluor white, and Fungiqual are to be had for detecting (Garcia *et al.*, 2018).

2.5.5.5 Preventing exposure

Infected with AIDS who have $CD4^+$ counts <200 cells/cells/µl should avoid untreated water sources. Additional recommendations include general attention to handwashing and personal hygiene, avoiding eating undercooked meat or seafood, and limiting exposure to animals known to be infected with microsporidia. The precautions described in the section on cryptosporidiosis also apply to microsporidiosis (Ghoyounchi *et al.*, 2017; Shadduck & Pathobiology, 2019).

2.5.5.6 Preventing Disease

Because chronic microsporidiosis occurs primarily in patients with advanced immunodeficiency, appropriate initiation of ART before the patient becomes severely immunosuppressed should prevent this disease. No specific chemoprophylactic regimens are known to be effective in preventing microsporidiosis (Ghoyounchi *et al.*, 2017; Shadduck & Pathobiology, 2019)

2.5.5.7 Treating Disease

Data suggest that treatment with ART enables a patient's defences to eradicate microsporidia, and administration of ART with immune restoration (an increase in $CD4^+$ count to >100 cells/cells/µl) is associated with resolution of symptoms of enteric Microsporidiosis, including infection because of *E. bieneusi*. All sufferers therefore should be provided ART as a part of the initial management of *Microsporidia* infection. They have to take delivery of fluid support if they have symptoms of diarrhea and dehydration (Anane & Attouchi, 2010; Rodríguez-Pérez *et al*., 2019). Intestinal Microsporidia may be treated with metronidazole and albendazole (National Institutes of health, 2019).

2.6 Prevalence of Opportunistic Intestinal Parasites among HIV Seropositive Individuals in Different Parts of Ethiopia

Like in many other developing countries, infection with intestinal parasites are widely distributed in Ethiopia largely due to the low level of environmental and personal hygiene, contamination of food and drinking water that results from improper disposal of human excreta. Also, a lack of awareness of simple health promotion practices is a contributing factor (Endeshaw, 2005).

The report of the Ethiopian Ministry of Health (MOH, 2006) confirms that more than half a million annual visits to the outpatient services of the health institutions are due to intestinal parasitic infections. However, this report may be an underestimated, because most of the health institutions lack appropriate diagnostic methods to detect low levels of parasite burden. Also, some of the diagnostic methods for specific intestinal parasites, especially for the newly emerging opportunistic intestinal parasites are not available to peripheral health institutions. In Ethiopia, various cross-sectional studies were conducted on the prevalence of opportunistic and other intestinal parasites among HIV seropositive individuals (Abebech Yitagsu., 2018).

Based on the available literature *Cryptosporidium* species is the most studied common coccidian intestinal parasitic infection among HIV/AIDS patients. The highest prevalence of this infection reported from Gambie higher clinics Bahir Dar (43.6%) and the lowermost prevalence reported in Komblecha (1.4%).

Cystoisospora belli likewise commonly reported following to *Cryptosporidium* species 0.5% up to 22.5% prevalence rate from Komblecha and St. Paul Hospitals in Addis Ababa

respectively in the above study areas. *Cyclospora cayetanensis* is the least reported intestinal protozoan. The highest prevalence recorded in Arbaminch Hospital, which is 5.9%. *Balsocystis homins* is not commonly reported in different study in Ethiopia. But the highest prevalence rate recorded from Gambia higher clinics Bahir Dar 10.8%. All selected studies were used diagnostic methods for identification of this parasitic infection was Modified Ziel Neelson staining techniques only one study was used molecular diagnosis (Appendix A).

Based on review the commonly identified risk factors associated with these parasitic infections were $CD4^+$ cell count, poor sanitation, contact with cattle, and source of drinking water, poor handwashing, unwashed vegetables, and usage of toilet (appendix A).

2.7 Conceptual framework of associated risk factors with OIPI among HIV/AIDS patients

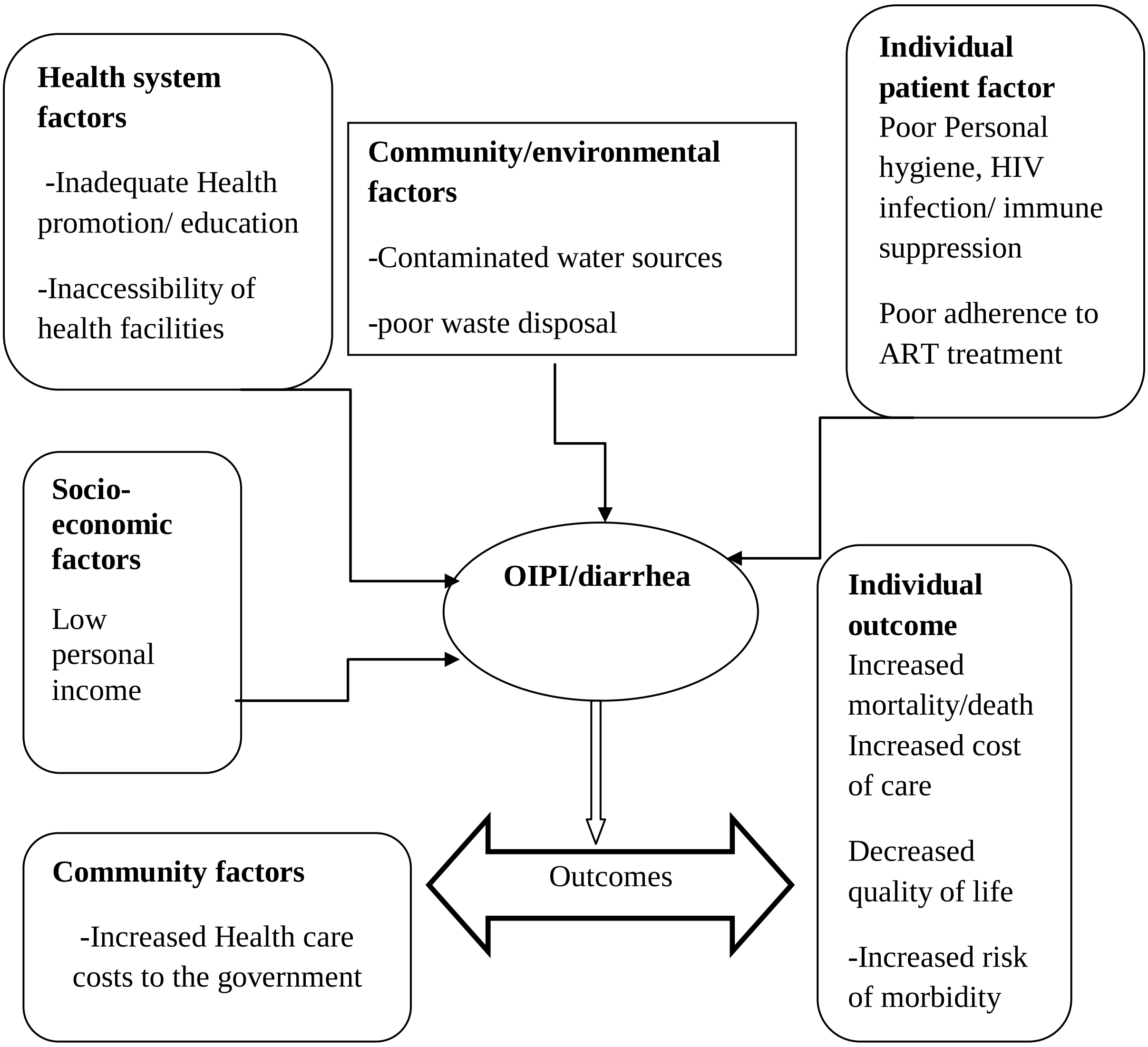

3 MATERIALS AND METHODS

3.1 Study Area

The study was carried out in Debre Tabor General Hospital in Debre Tabor city. Debre Tabor is located in the South Gondar Zone, about 100 kilometers southeast of Gondar and 50 kilometers east of Lake Tana in Amhara Region, Ethiopia,. Debre Tabor was the capital of Ethiopia under two Emperors Tewodros II and Yohannes IV. This historic town has a latitude and longitude of 11°51′N 38°1′E with an elevation of 2,706 meters (8,878 ft.) above sea level. The average annual rainfall of the area is 1553.7mm and average temperature ranges from 10°C-18℃(Debre Tabor city administration office, personal communication, 2020).

Debre Tabor town has one governmental (one general) Hospital, three governmental health centers, three private clinics, and three private pharmacies. Both governmental and private health institutions provide health care services on prevention and control of communicable diseases and numerous activities on non-communicable diseases through providing diagnostic, preventive, therapeutic, and rehabilitative services (DebreTabor city administration office, 2020). Its population is estimated to above 87,627 (Central Statistical Agency 2007).

Debre Tabor General Hospital is the largest Hospital in South Gondar Zone, has been providing services to the 2.3 million populations in its catchment areas of the Amhara Regional State. The hospital was founded in 1923 E.C Now the Hospital is emerging as a shining hot spot for advanced medical care and treatment in the North central parts of Ethiopia with a total capacity of above 110 inpatient beds in five major departments. It has a range of specialties in 57 occupational categories and a total of 325 active health professionals (DebreTabor Hospital, December 15, 2019).

The hospital has been providing counseling and testing service for HIV since the middle of the 1990s E.C. Screening, as well as treatment and follow-up of HIV/AIDS patients, is one of the routine services provided in the hospital. Currently, the hospital gives service to 2210 ART patients. The study area was selected by purposive sampling due to the presence of a relatively high load of HIV/AIDS patients receiving ART during the study period, no updated epidemiological data, and its relative accessibility. As a university hospital, it plays an important role in teaching medical and other health sciences, students (DebreTabor Hospital, December 15, 2019). The district has local access to clean water. As a result, people were

forced to use various unprotected water sources such as man-made well (authors' observation).

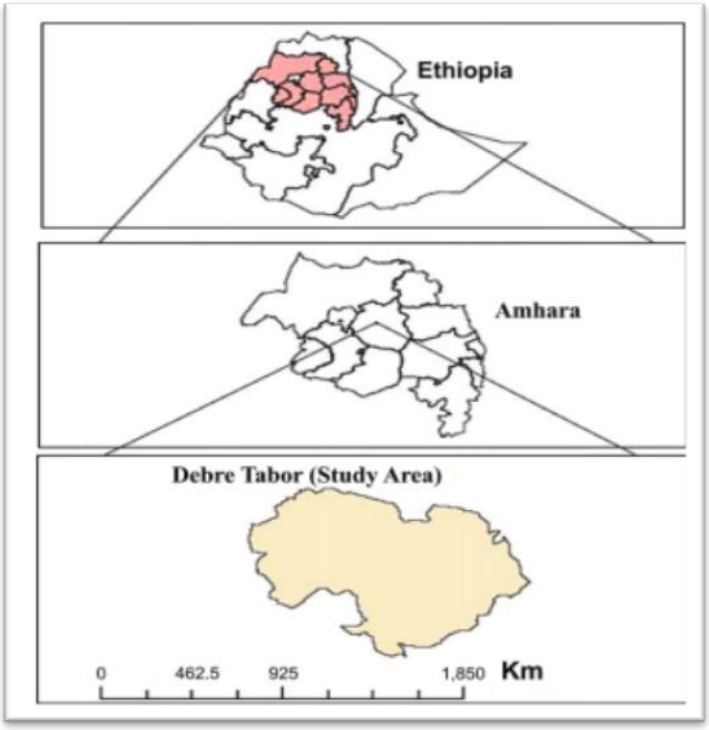

Figure 9 Location of study area of Debre Tabor town (Afera Halefom *et al*., 2018)

3.1.1 Operational Definitions

Adherence- Taking medications or other treatments exactly as instructed by a health care provider.

Antiretroviral Therapy- The daily use of a combination of HIV medicines (called an HIV regimen) to treat HIV infection**.**

ART patient- is a person who was found HIV positive eligible and started ART.

Immunocompromised- When the body is unable to produce an adequate immune response.

Diarrhea- is defined as the passage of loss or watery stool at least three times in 24 hours.

Opportunistic Parasites- are denoting an organism capable of causing disease only in a host whose resistance is lowered (by other diseases or drugs etc).

3.2 Study Design and Period

A hospital-based cross-sectional study was conducted for determining the prevalence of OIP infections and their associated risk factors among HIV/AIDS patients attending ART at Debre Tabor General Hospital, Amhara, Ethiopia from December 2019 to February 2020.

3.3 Study Population

All HIV positive patients who registered in Debre Tabor General Hospital's ART clinic were considered as the source of population. All the HIV positive patients on ART registered and who came to the ART clinic for their follow up during the study period were used as the study population.

3.3.1 Inclusion Criteria

All the HIV/AIDS patients who signed the consent (Appendix C) and attending ART clinics during the study period and who were willing to participate in the study were included in the present study.

3.3.2 Exclusion Criteria

Those participants who have been taking anti-parasitic medication during the last three months were excluded from this study. Also, those with history or diagnosis of any other acute or chronic disease-causing immune-suppression were excluded.

3.4 Sample Size Determination and Sampling Techniques

The sample size was determined using the single proportion formula (Charan & Biswas, 2013).

$n = (Z^{\wedge}2\ p(1-p))/e^{\wedge}2$ n = sample size, Z=confidence level, e = margin of error, P = expected prevalence or proportion.

Since in the study, the value of P (expected prevalence) was unknown P=50.0% was used to determine the sample size. Using a 5% precision level, 50% prevalence and 10% contingency was added, then the entire sample size was determined to be 422 individuals. The likelihood of all HIV/AIDS patients who were consented and that come into ART clinics for follow up treatments were included until the required sample size was come to be.

3.5 Study Variables

The study was focused on the following independent variables (socio-demographic status, environmental and personal hygiene, the status of ART adherence, clinical conditions, and

immune status of the patient). The prevalence of opportunistic intestinal infections was considered as the response variable of this study.

3.6 Data Collection Techniques

3.6.1 Questionnaire survey

A structured questionnaire was prepared and pre-tested with 20 HIV patients who did not participate in the study and who were attending the ART clinics of Debre Tabor General Hospital, to evaluate the quality and strength of the questionnaire. Directional view and awareness about the objective of this study was given to actively participate in filling the questionnaire by ART clinician and principal investigators. The questionnaire was originally prepared in English and then translated to Amharic local language and back to English to obtain content validity. After modifying and assuring its quality, the pre-tested questionnaires were administered for respondents to generate data on risk factors of OIPIs such as questions related to socio-demographic characteristics, behavioral habits, hygienic, sanitary conditions, environmental conditions and clinical information.

3.7 Data of $CD4^{+}$ Lymphocyte Counts

The recent CD4+ T cell count data was taken from patients' medical records. The count was determined by DebreTabor General Hospital by using fluorescence-activated cell scanning (FACS) analysis or flow cytometer (Becton Dickinson Immuno-cytometry system, and Jose, Calif., USA).

3.8 Stool Sample Collection and examinations

The proper method for the collection of stool samples was provided to ensure the favorable condition of the stool sample. Also, an oral description and specific instruction on proper handling and avoidance of contamination of the stool specimen were given to the entire participant by the investigator. Disposable plastic cups with applicator spoon for collection of stool sample was provided for study subjects by labeling them with their unique codes, and after which they were told to bring about 5-10 grams of fresh stool specimen each.

The collected stool samples were immediately preserved with 10 % of the formal solution up to the time of microscopy which helps in preserving the shape and size of protozoan and helminths parasites and preventing further development. The stool samples were marked or coded with the name of ART patients for the respective stool samples identification. The

stool samples were then processed using various standards operation laboratory parasitological examination procedures. Microscopic examination was carried out to identify the presence and type of OIPI (WHO, 2019).

3.8.1 Stool Sample Collection and examination's

The appropriate specimen containers and applicator sticks for each consented participant were provided by the investigator to bring 5-10 grams or 5-6 ml (if a sample is watery) from their fresh stool sample. Investigator followed the laboratory principles and techniques of the World Health Organization to get the appropriate samples. Each fresh stool specimen was processed using the following methods.

3.8.2 Parasitological Laboratory Examination Procedure

3.8.2.1 Direct wet mount

Direct wet mount smear was examined immediately by using normal saline. Observe under a light microscope at 100X and 400 X magnifications to detect the presence of motile parasite stages. The remaining sample was preserved with 10% formalin and examined by the formol-ether concentration technique and Modified Ziehl-Neelsen staining method (Garcia *et al.*, 2018; WHO, 2019).

3.8.2.2 Formol-ether concentration method

Briefly, one gram of stool was taken from the preserved stool and mixed with a 10% formalin solution and sieved through double-layered gauze. The filtrate was transferred to a 15 ml conical centrifuge tube and added 7 ml of formalin saline using a plastic pipette and 3 ml of diethyl ether. Then the content was shock vigorously and centrifuged for five minutes at 3000 rpm. Finally, the smear was prepared from the sediment and examined for the presence of ova, cyst, and larvae of different intestinal parasites under the light microscope with a magnification of 100X and 400X (Garcia *et al.*, 2018).

3.8.2.3 Modified Ziehl Neelsen acid-fast stain

A small portion of the fresh stool sample was processed for the detection of *Cryptosporidium, Cystoisospora belli,* and *Cyclospora cayetanensis* oocyst. A thin smear was prepared directly from the sediment of the concentrated stool and allowed to air dry. The slides were then fixed with methanol for five minutes and stained with carbol fuchsine for 20 minutes. After washing the slides in tap water, they were decolorized with 1% hydrochloric acid in methanol

alcohol for one to three minutes and stained in methylene blue for one minute. The slides were then washed in tap water and observed under a light microscope with magnification using oil immersion objective lenses (Garcia *et al*., 2018).

3.9 Data Quality Control

The questionnaires were first pre-tested before the collection of actual data and were checked for its completeness after collection. To make certain quality control, all the laboratory procedures including collection and handling of specimens were carried out following standard protocols (NCCLS, 1997). To ensure accurate identification of parasite species, bench aids for the diagnosis of the intestinal parasite (WHO, 2019) and diagrams of various parasite trophozoites, cyst, and oocyst was reviewed. To minimize missed parasite identification and discrepancy, each slide was examined by a laboratory technologist and finally by the principal investigator. The result of each laboratory examination was recorded carefully in a well-prepared format. The data were analyzed using the SPSS version 23-software program.

A direct stool examination was performed within 30 minutes to avoid delay. The negative result was reported after a single stool sample was tested at least three times. $CD4^+$ T cell classification and definition were made based on the WHO criteria. In all investigators was followed a standard protocol WHO to collect all the necessary data.

3.10 Data Analysis

Descriptive statistics was used to understand the property and the pattern of data collected from respondents. The data obtained from questionnaires were entered through the EPI-INFO 7 computer program for pre-coding data and then exported to SPSS version 23.

A Chi-square test was applied to determine the association of parasitic infection and immunity level. The logistic regression model was employed to assess potential risk factors. The strength of associations was evaluated by odds ratios (OR) and 95% confidence intervals (CI). Variables that showed co-linearity were lost, while the remaining model variables $P<0.25$ in bivariate analysis were entered in to multi-variable logistic regression. The given statistical test was reported significant when its value was less than 0.05.

3.11 Ethical Considerations

The data were collected after ethical approval was obtained from the research Ethical Review Committee of Science College, Bahir Dar University. Informed consent was obtained from the patient or families for children. Besides, the information kept by top confidentiality and privacy and collected in a unique code number.

4 RESULTS

4.1 Socio-Demographic Characteristics of the Study Participants

A total of 384 study participants were included in the study. Of these, 222(57.8%) were females and the remaining 162(42.2%) were males. The age of the study participants ranges from 9 to 65 years with mean of and standard deviations of 34.30 and 11.073 years respectively (Table 1).

Of the study participants, 262(68.2%) reside in the urban area, of whom 127(33.1%) were civil servants. Most study participants 313(82.5%) were educated and 241(62.8%) were married.

About 256 (66.6%) and 75 (19.5%) of the study participant's replied that they used tap water for drinking and washing purposes, respectively. Whereas the other 128 (33.3%) and 309 (80.552%) replied that they used unprotected water for drinking and washing purposes. Among 384 study participants, 94 (24.5%), 119 (31.0%) and 171 (44.5%) did already started ART before 25 months, 25 to 59 months, and 60 months ago, respectively. From those respondents, 90 (23.4%) had poor adherence to ART treatments.

Regarding their $CD4^+$ T cell counts, 56(14.6%), 87(22.7%), 98(25.5%) and143(37.2%) patients had $CD4^+$ T-cell counts below 200, between 200 and 349, 350 to 499 and above500 cells/µl of blood, respectively. The majority of (48.1%) patients had $CD4^+$ cell count lies between 200 cells/µl and 500 cells/µl. regarding, the history of diarrhea, 75 (19.5%) of study participants had this symptom in the last three months. Regarding environmental and personal hygiene, 194 (50.5%) respondents practice open waste disposing mechanism, 310 (80.9%) study participants had used latrine (table 2). From the total respondent's the habit of washing their hands before a meal and after the toilet was 90% and 19.27%, respectively. Regarding their latrine facility, 80.9% of sample populations were used in toilet houses.

The majority of the study participants (69.8%) had animal contact and 73.4% of the study participants had a regular practice of trimming their nails. About 52.3% and 57.3% of the respondents replied that they used unprotected water for washing fruits and vegetables, respectively. From the total study participants, the majority (62.2%) of them did not consider the safety of water and food while travelling.

Table 1 Socio-demographic, hygienic habit and clinical information of patients with HIV/AIDS taking ART at Debre Tabor General Hospital (DTGH), ART clinic from December 2019 to February 2020, cross-sectional study (N=384).

Variables	Category	Frequency	Relative frequency (%)
Sex	Male	162	42.2
	Female	222	57.8
Age (year)	Below 18	32	8.3
	18-24	95	24.7
	25-45	215	56.0
	Above 45	42	10.9
Marital status	Single	35	9.1
	Married	241	62.8
	Divorced	108	28.1
Occupation	Student	45	11.7
	Laborer	58	15.1
	Merchant	35	9.6
	Non-employed	48	12.5
	Farmer	68	17.7
	Civil servant	128	33.3
Education level	No regular	71	18.5
	Primary	100	26.0
	Secondary	89	23.2
	above secondary	124	32.3
Handwashing habit before the meal	Always	349	90.9
	Sometimes	35	9.1
CD4 count in cells/µl	Below 200	56	14.6
	200-349	87	22.7
	350-499	98	25.5
	Above 500	143	37.2

Characteristic	Category	Frequency	Relative frequency (%)
Adherence to ART	Y1es	90	23.43
	No	294	76.6
Diarrhea conditions	Yes	75	19.5
	No	309	80.5
Waste disposing mechanisms	Open deification	194	50.5
	Closed defecation	190	49.5
Trimming fingernails	Always	282	73.4
	Often	99	25.8
Animal contact	Yes	116	30.2
	No	268	69.8
Source of water for washing vegetables	Un protected	220	57.3
	Protected	164	42.7
Source of drinking water	Tap water	256	66.7
	Unprotected/open	128	33.3
Source of water for washing fruits	No-wash	84	21.9
	Unprotected	201	52.3
	Protected	99	25.8
Use latrine	Yes	310	80.9
	No	74	19.1
Type of washing water	Unprotected	309	80.5
	Tap water	75	19.5
Handwashing habit after toilet	Always	74	19.27
	Sometimes	115	29.94
	No	195	50.78
Considering the safety of water and food when travel	Yes	145	37.8
	No	239	62.2

Note: Protected water source=tap water and bottled water, Unprotected water source= stream, .river, spring water well, rain water and other sources

4.2 Major Intestinal Parasitic Species Identified among the Examined HIV/AIDS Patients

A total of 384 stool samples were examined for intestinal parasitic infections. Among these 122 (31.7%) were found positive for an intestinal parasite (both opportunistic and non-opportunistic parasite species). The overall prevalence of opportunistic parasite infections was found to be 69/384(17.96%) (Table3). There were also mixed infections with non-opportunistic protozoan and helminths parasites in 12 (3.125%) individuals.

In this study, eight species of intestinal parasite species were detected. Of these parasites, five were belonged to protozoan and the remaining three belonged to helminthic parasites. Of these 110 (28.7%) were infected with single parasites. The most frequent parasite species detected was *Cryptosporidium* species (8.59%) followed by *Cystoisospora belli* (6.77%) and *Cyclospora cayetenensis* (2.60%) from the opportunistic intestinal protozoan parasites. Regarding the non-opportunistic intestinal parasites, the prevalence of *Entamoeba histolytica/dispar, Giardia lamblia, Ascaris lumbricoides, Hookworm* and *Trichuris trichiura* was 4.9%, 2.86%, 3.1%, 1.56%, and 1.3%, respectively (Table 2).

There were (3.1%) cases double infections opportunistic intestinal protozoan with protozoan and helminths were observed. Among the double infections, all (12) cases displayed the coccidian parasites (*Cryptosporidium* species, *Cyclospora cayetanensis and Cystoisospora belli)* had three, six and three mixed infections, respectively (Table 3).

Table 2: Intestinal Parasites detected in HIV/AIDS patients who were on ART in DTGH from December 2019 to February 2020 (N= 384).

Detected parasite name	Frequency	Relative frequency (%)
**Monoparasitism**	**110**	**28.7**
**Protozoan parasites**	**87**	**22.6**
Cryptosporidium species	30	7.8
Entamoeba histolytica/dispar	19	4.9
Giardia lamblia	11	2.86
Cyclospora cayetanensis	4	1.04
Cystoisospora belli	23	5.98
**Helminths parasites**	**23**	**5.98**
Ascaris lumbricoides	12	3.1
Hookworm	6	1.56
Trichuris trichiura	5	1.3

Table 3: The Prevalence of mixed infection of Coccidian parasite with non-opportunistic intestinal parasites among HIV/AIDS patient in DTGH from December 2019

Detected opportunistic parasite (no. of patients)	No of patients with mixed infection n (%)	Co-infected with (n)
Cryptosporidium species (33)	3(0.78)	*Giardia lamblia* (2), *Ascaris lumbricoides* (1)
Cyclospora cayetanensis (10)	6(1.56)	*Giardia lamblia* (3), *Ascaris lumbricoides* (1) *Hookworm* (2)
Cystoisospora belli (26)	3(0.78)	*Ascaris lumbricoides* (1), *Entamoeba histolytica/dispar* (2)
**Total (57)**	**12(3.125)**	**12**

4.3 Prevalence of Opportunistic Intestinal Parasites by Age and Sex

The prevalence of OIPI among HIV patients with sex and age are summarized and presented in Table 5. The overall occurrence of OIPI in this study was 17.9%. The prevalence of

Cryptosporidium species, *Cystoisospora belli, and Cyclospora cayatenensis* in both sex and age were 33(8.5%) 26(6.77%) and 10(2.60%), respectively (Table 5).

The prevalence rate of OIPI was relatively higher in females 36(9.37%) than males 33(8.5%). *Cryptosporidium* species was detected in 17(4.42%) and 16(4.16%), *Cystoisospora belli* was detected in 13(3.38%) and 13(13.38%), *Cyclospora cayatenensis* was identified in 6(1.56%) and 4(1.04%) in male and female participants, respectively.

As shown in Table 5, the age of the participants was grouped into, below 18, 18-30, 31-50 and above 50. Among those age groups 5(1.30%), 23(5.98%), 38(9.89%), and 4(1.04%) were positive to OIPI respectively. The prevalence of OIPI was higher in the age group of 31-50 than in other age groups.

The prevalence of *Cryptosporidium* species, *Cystoisospora belli* and *Cyclospora cayatenensis* in term of age was 1.06%, 0.26 % and 0% (Below 18 year); 2.08%, 2.34% and 1.30% (18-30 year); 4.4%, 1.3% and 4.1% (31-50 year) and 1.04%, 0% and 0% (Above 50 year) respectively. According to the data *Cryptosporidium* species reported in all age groups. *Cyclospora cayatenensis* was not found below eighteen year and above 50-year. *Cystoisospora belli* was also, not seen above 50-year-old participants.

Table 4: Prevalence of Major Opportunistic Intestinal Protozoan Parasitic Species by age and sex among HIV/AIDS patients Who Visited DTGH ART clinics from December 2019 - February 2020 N=384.

Age group in years	Sex	No. (%)	The total prevalence of OIPI	Identified OIP species in number (%)		
				Cry	Cyclo	Iso
			No.Pos (%)	No.Pos (%)	No.Pos (%)	No.Pos (%)
Below 18	Male	19(4.9)	4(1.04)	3(0.78)		1(0.26)
	Female	13(3.4)	1(0.26)	1(0.26)		
	Both	32(8.33)	5(1.30)	4(1.04)		1(0.26)
18-30	Male	40(10.4)	8(2.08)	2(0.52)	3(0.78)	3(0.78)
	Female	84(21.9)	14(3.6)	6(1.56)	2(0.52)	6(1.56)
	Both	124(32.2)	23(5.98)	8(2.08)	5(1.30)	9(2.34)
31-50	Male	89(23.2)	21(5.46)	9(2.34)	3(0.78)	9(2.34)
	Female	111(28.9)	17(4.42)	8(2.08)	2(0.52)	7(1.82)
	Both	200(52.08)	38(9.89)	17(4.4)	5(1.30)	16(4.1)
Above 51	Male	14(3.6)	3(0.78)	3(0.78)		
	Female	14(3.6)	1(0.26)	1(0.26)		
	Both	28(7.29)	4(1.04)	4(1.04)		
All	Male	162(42.18)	36(9.37)	17(4.42)	6(1.56)	13(3.38)
	Female	222(57.8)	33(8.5)	16(4.16)	4(1.04)	13(3.38)
	Both	384(100)	69(17.9)	33(8.5)	10(2.60)	26(6.77)

Note: No. =number of examined, No.pos. =number of positive, Cry. =*Cryptosporidium* species, Iso=*Cystoisospora belli,* Cyclo=*Cyclospora cayatenensis*

4.4 Major factors associated with opportunistic Intestinal parasites

In this study, several factors were considered to assess factors that contribute to OIPIs. Binary and multivariable logistic analyses were calculated. Multivariable logistic analysis was conducted after adjusting variables that were p< 0.25 in the binary logistic analysis

The present study shows that males (9.11%) were more affected by OIPs than females (8.85%). However, the difference was not statistically significant (AOR=0.860, 95% CI: 0.3492-2.115 p=0.742). The high rate of OIPIs was observed in the age group of 30-49 years (17.9681%), but the difference was not significant (AOR=1.250, 95% CI: 0520-3.006, p=0.618).

In multivariate logistic regression analysis, patients who lived in rural residents were less likely to acquire OIPIs than patients who had lived in urban (AOR=0.197, 95% CI: 0.053-0.734, P= 0.015). Even though, the rest socio-demographic factors were significant in bivariate analysis, but not significance in multivariate analysis (Table 5).

Patients whose $CD4^+$ T-cell count was <200cell/µl were more likely to be infected by OIPs (AOR=49.08, 95% CI: 9.440-228.777, P= 0.000). Patients who had poor adherence to ART treatment were more likely infected with OIPs (AOR=7.427, 95% CI 2.488-22.172, P=0.00).

Study participants who replied yes for diarrheal history less than three months were more likely infected with OIPs (AOR=7.063, 95% CI: 1.882-26.512 P=0.004). Patients who responded often trimming of fingernails were more likely infected with OIPs (AOR=3.665 95% CI: 1.040-12.918 P=0.043).

Moreover, patients who drunk water from the unprotected source and no handwashing habit after the toilet were 4 times more likely infected with OIPs (AOR=14.721, 95 % CI: 3.349-64.71 P=0.000) and (AOR=10.409 95% CI: 1.398-77.497 P= 0.022), respectively. However, in multivariate analysis, the majority of the study variables were not significant (Table 6). `

.

Table 5: Bivariate and multivariable logistic regression analysis of opportunistic intestinal parasitic infection with socio-demographic factors among ART attending patients in DTGH, from December 2019 to February 2020 (N=384).

Risk factors	Category	Number of examined cases (%)	Rate of OIP number (%)	COR (95 % CI)	P value	AOR (95% CI)	p-value
Sex	Male	162(42.18)	35(21.6)	1.52(0.903-2.57)	0.114	8.60(0.34-2.115)	0.742
	Female	222(57.81)	34(15.3)	1.00□			
	18 and below	32(8.33)	5(15.6)	0.93(0.265-3.24)	0.904		
Age	18-29	95(24.73)	14(14.7)	0.86(0.321-2.33)	0.773		
	30-49	215(55.98)	43(20.0)	1.25(0.520-3.006)	0.618		
	Above 50	42(10.93)	7(16.7)	1.00□			
	Single	35(9.11)	11(31.4)	2.45(1.016-5.92)	0.46		
Marital status	Married	241(62.76)	41(17.0)	1.09(0.59-2.03)	0.768		
	Divorced/widowed	108(28.12)	13(15.9)	1.00□			
	Civil servant	128(33.33)	9(7.1)	1.00□			
Occupation	Student	45(11.71)	7(15.6)	2.44(0.85-6.98)	0.098	2.789(0.53-14.535)	0.223
	Laborer	58(15.1)	21(36.2)	7.5(3.16-17.8)	0.023	4.615(0.810-26.289)	0.085
	Merchant	37(9.63)	3(8.1)	1.17(0.30-4.55)	0.82	0.556(0.063-4.880)	
	Non employed	48(12.5)	0(0.0)	0.000(0.000	0.997		
	Farmer	68(17.7)	29(42.6)	9.8(4.28-22.56)	0.018*	3.143(0.481-20.531)	0.232
	No regular education	71(18.48)	26(36.6)	0.58(0.264-1.011)	0.002*	0.915(0.235-3.557)	0.898
Education level	Primary	100(26.04)	23(23)	0.296(0.138-.634)	0.004*	0.783(0.186-3.300)	0.739
	Secondary	89(23.17)	13(14.6)	0.104(0.042-.255)	0.013*	0.850(0.154-4.691)	0.852
	above secondary	124(32.29)	7(5.6)	1.00□			
Residence	Urban	262(68.22)	36(13.7)	1.00□			
	Rural	122(31.17)	33(27.0)	2.33(1.36-3.96)	0.002**	0.197(0.053-0.734)	0.015*

Note AOR: Adjusted odd ratios, CI: confidence interval, COR: Crude odd ratio, Reference category:˟ significance association*

Adjusted ratio only computed for p≤ 0.25 in the bivariate analysis and p<0.05 for multivariate analysis

Table 6 : Bivariate and multivariable logistic regression analysis of opportunistic intestinal parasitic infection with hygienic and clinical informations among ART attending patients in DTGH, from December 2019 to February 2020 (N=384).

Risk factors	Category	Number of examined cases (%)	Rate of OIP number (%)	COR (95 % CI)	P value	AOR (95% CI)	p-value
$CD4^+$ T-cell count in cells/µl	Below 200	56(14.58)	40(71.40	86.87(27.48-274.56)	0.00*	49.08(9.440-228.777)	0.000*
	200-349	87(22.65)	17(19.5)	8.43(2.736-26.03)	0.012*	2.678(0.513-13.993)	0.243
	350-499	98(25.52)	8(8.2)	3.08(0.90-10.559)	0.072*	3.107(0.649-14.867)	0.156
	Above 500	143(37.27)	4(2.8)	1.00□			
Perfect ART adherences’	Yes	274(71.35)	15(5.5)				
	No	110(28.64)	54(49.1)	16.65(8.77-31.60)	0.00*	7.427(2.488-22.172)	0.000*
ART started in months ago	Below 24 months	290(75.52)	48(16.6)	1.311(0.661-2.59)	0.438		
	25-59 month	31(8.07)	8(25.8)	1.75(0.74-4.15)	0.202		
	Before 60 month ago	63(16.40)	13(20.6)	1.00□			
Diarrhea condition	Yes	75(19.53)	32(42.7)	5.47(3.08-9.69)	0.000*	7.03(1.882-26.512)	0.004*
	No	309(80.46)	37(12.0)	1.00□			
Waste disposing mechanism	Open deification	194(50.52)	54(27.8)	4.50(2.436-8.31)	0.018*	0.37(0.106-1.332)	0.130
	Closed defecation	190(49.47)	15(7.9)	1.00□			
Trimming fingernails	Always	283(73.6)	27(9.5)	1.00□			
	Often	101(26.30)	42(41.6)	6.750(3.855-11.89)	0.000*	3.665(1.040-12.918)	0.043*
Animal contact	Yes	116(30.20)	45(38.8)	6.444(3.675-11.298)	0.012*	1.104(0.324-3.759)	0.875
	No	268(69.79)	24(9.0)	1.00□			
Source of water for washing vegetable	Un protected	220(57.29)	58(26.4)	4.98(2.52-9.84)	0.002*	2.10(0.610-7.24)	0.240
	Protected	164(42.70)	11(6.7)	1.00□			
	Tap	256(66.66)	17(6.6)	1.00□			

Source of drinking water	Unprotected/open surface water	128(33.33)	52(40.6)	9.619(5.251-17.622)	0.00*	14.721(3349-64.71)	0.000*
	No wash	84()21.09	34(40.5)	16.10(5.424-48.00)	0.002*	0.792(0.107-5.880)	0.819
Source of water for washing fruits	Un protected	201(52.34)	31(15.4)	4.33(1.484-12.64)	0.017*	0.994(0.189-5.221)	0.994
	Protected	99(25.78)	4(4.0)	1.00□			
Use latrine	Yes	310(80.72)	44(14.2)	1.00□			
	No	73(19.01)	25(34.2)	3.15(1.764-5.62)	0.013*	1.068(0.278-4.111)	0.924
Type of washing water	Unprotected	309(80.46)	66(21.4)	6.519(1.99-21.35)	0.012*	1.620(0.190-13.826)	0.659
	Protected	75(19.53)	3(4.0)	1.00□			
Handwashing habit before the meal	Always	349(90.88)	56(16.0)	1.00□			
	Sometimes	35(9.11)	13(37.1)	3.092(1.471-6.45)	0.03*	1.327(0.294-5.987)	0.713
Handwashing habit after toilet	No	195(50.78)	54(27.7)	9.064(2.738-30.005)	0.00*	10.409(1.398-77.497)	0.022*
	Sometimes	115(29.9)	12(10.7)	2.76(0.75-10.13)	0.126	3.503(0.409-29.996)	0.253
	Always	74(19.27)	3(4.1)	1.00□			
Consider safety of water and food when you travel	Yes	236(61.48)	59(25.0)	4.60(2.27-9.32)	0.052*	1.693(0.525-5.464)	0.378
	No	148(38.54)	10(6.8)	1.00□			
Eating food by sharing	Yes	145(37.7)	54(22.6)	1.00□			
	No	239(62.23)	15(10.3)	0.395(0.214-.731)	0.06*	0.848(0.259-2.771)	0.785

Note AOR: Adjusted odd ratios, CI: confidence interval, COR: Crude odd ratio, Reference category:* significance association

Adjusted ratio only computed for p≤ 0.25 in the bivariate analysis and p<0.05 for multivariate analysis

4.5 Association of OIP with CD4+ T-Cell Counts

The proportion of OIPIs was significantly higher in patients with a $CD4^+$ count <200 cells/µl when compared with the other group $CD4^+$ count > 200. The $CD4^+$ counts of 56 individuals were < 200 cells/µl. Of those 42 were infected by OIPI. *Cryptosporidium* species (n=30) was the most common pathogen followed by *Cystoisospora belli* (n=23), *Cyclospora cayetanensis* (n=4) and double infection (coccidian parasite with other non-opportunistic intestinal parasites) (n=12). Of (87) patients with $CD4^+$ count 200-349 cells/µl, OIPI were detected in (n=21) patients. Fifteen patients had *Cryptosporidium* and six were mixed infections.

On the other hand, 98 study participants had $CD4^+$ cell count between 250 cells/µl and 499 cell/µl. From those only four cases were infected, (2) with *Cryptosporidium* species, (1) with *Cyclospora cayetanensis* and (1) with double parasitic infections. The remaining 143 study participants had cell count above 500 only two cases were infected, one in *Cryptosporidium* and one in double parasitic infections (table 6).

Table 7: The association of each coccidian parasite with $CD4^+$ T-cell count status of HIV-infected patients who were on ART in DTGH from December 2019 to February 2020.

Detected parasite	Frequency of parasite number (%)	Count CD4+ cells/µl				Chi-square	P-value
		<200 N=56	200-349 N=87	350-499 N=98	>500 N=143		
		No.pos (%)	No.pos (%)	No.pos (%)	No.pos (%)		
Cryptosporidium species	30 (43.4)	12(21.4)	15(17.24)	2(2.04)	1(0.69)	39.374	0.000*
Cyclospora cayetanensis	4 (5.79)	3(5.3)	-	1(1.02)	-	12.539	0.006*
Cystoisospora belli	23(33.33)	23(41)	-	-	-	143.29	0.000*
Double-parasitic infections	12 (17.48)	4(7)	6(6.89)	1(1.02)	1(0.69)	11.287	0.010*
Total	69(100)	42(70.49)	21(24.13)	4(4.08)	2(1.3)	137.448	0.000*

Note: No.pos (%) = number of positive N= Total number of participant * = significanct association

5 DISCUSSION

Even after free ART availability, intestinal parasites are still a big concern (Bachur *et al*., 2008). Most morbidity and mortality of advanced AIDS is associated with opportunistic intestinal parasites that cause incapacitating infections in immunocompromised individuals with low immune status as compared to the immune-competent individuals. HIV induces an immune-deficient state that favors infection by intestinal parasites, which, in turn, contribute to worsening the patient's clinical condition by causing malnutrition, weight loss, and chronic diarrhea (Barcelos *et al*., 2018; Avilés *et al*., 2020).

The overall prevalence of IPI (31.17%) reported by this study was lower than the findings of other similar crossectional studies conducted among ART patients in different places, such as Gambie Hospital Bahir Dar 80.3% (Abebe Alemu *et al* 2011), Jimma Ethiopia 39.5% (Zeynudin Ahmed *et al.*, 2013), Wolayta, Ethiopia 63.5% (Menberluel Mathewos, 2014), Mettu Karl hospital 62.5% (Solomon Yeshanew, 2017). Jimma, Ethiopia 45.0% (Yonatan Kindie and Shiferaw Bekele, 2016); Cameroon, 82.3% (Dickson Shey Nsagha, Zida *et al.,* 2016*)*, Ouagadougou, Burkina Faso 73.3% (Abdourahamane, 2017), Karnataka, India 49% (Rajeshwari Prabhakar.R *et al*., 2015) Araba Minch Ethiopia 45.4% (Tigist Gerzmu *et al*., 2015), Butajira Ethiopia 35.9% (Dereje Gedle *et al.*, 2017), Bobo-Dioulasso, Burkina Faso 65.3%(Sangaré *et al.*, 2015), India 49.9% (Khalil *et al*., 2015). This lower rate of intestinal parasite observed in this study might be due to difference in the adherence of ART treatment.

However, it is almost in line with the ones reported from Dawero Ethiopia 30.6% (Fithamlak Bistegen *et al*., 2018), Arbaminch Ethiopia 28.18% (Getaneh Alemu *et al*., 2018), Eastern Ethiopia 28.6% (Zelalem Teklemariam *et al*., 2013), Gondar Ethiopia 29.1% (Tegegne Eshetu *et al*., 2017).

Also, this report is higher reported from Benine city, Nigeria 15.3% (Akinbo *et al.*, 2010), Felegehiwote Referal hospital Bahir Dar, 25.5% (Habtom Kiros *et al.*, 2015). Kombolcha Ethiopia 13.9% (Daniel Gebretsadik *et al*., 2018). Dessie Ethiopia 17.7% (Assefa Messaye *et al.*, 2013), France 17% (Pavie *et al*., 2012) and Aksum Ethiopia 26.4% (Tuom Gebrewahid *et al.*, 2019). This incompatible prevalence might be due to dissimilarities of topographical, sample size, the study population, study procedures and presence or absence of better follow-up and better cognizance of patients themselves in implementing treatment and prevention against intestinal parasites.

In the present study, the prevalence of OIPI among HIV/AIDS patients who attend ART was 17.96%. It is almost in line with studies from Arbamich 17.2% (Getaneh Alemu *et al*., 2018) and Jimma 15.38% (Zeynudin Ahmed *et al.*, 2013). This prevalence also has lower than the result from India 49% (Rao, 2016) and 34.9% (Namaji *et al*., 2020). This might be the characteristics and habit of study population dissimilarities

As far as the diversity of parasites, eight were identified. Five of them are protozoan and the remaining three were helminths. As shown in table 2 classic opportunistic pathogens (*Cryptosporidium* species, *Cystoisospora belli, Cyclospora cayetanensisi*) were sensed more common in the study area.

Cryptosporidium species was the most common OIP detected in HIV/AIDS patients who are on ART (8.5%). Similar results were seen studies from Ethiopia Arbaminch 8.63% (Getaneh Alemu *et al*., 2018), Wloayita Sodo 9.6% (Blatu *et al*., 2019), Dire Dwa eastern Ethiopia10% (Abebech Yitagesu 2018), Gondar 8.4% Nigus Telel, Yirgalem 9.4% (Alemsegde *et al.*, 2015, Addis Abeba 8.1% (Haile Eysues Adam *et al*., 2013). Also, the result of this study is small when equating with studies in different portions of Ethiopia from Addis Ababa 28.6%, (Endeshaw Tokola *et al.*,2005), Bahir Dar 15.3% (the nearest to the study area) Abebe Alemu *et al* (2011), Jimma 15.4%, Eastern Tigray 15.68%, Hawassa (20.1). The Lower rate infection observed in this study might be due to ART treatment as it elicits immunological responses. In contrast, it is higher reported in Adama, Afar and Dire Dawa 5% (Haile-eyesus Adamu and Beyene Petros, 2009), Dessie 1.5% (Assefa Missaye *et al*., 2013), Gondar 3.1%(Tegegne Eshetu e*t al*., 2017), Komblecha 1.4% (DanielGebretsadik *et al*., 2018).

The second most reported OIP was *Cystoisospora belli*, which accounts for 6.7%. The result is comparable with the findings from Wolayita Sodo 8.5% (Menbereleul Mathwose *et al*., 2014), Eastern Tigray 7.1% (Dinku Senbeta *et al.*, 2017), South Western 7.4% (Mohamed Awole *et al*., 2003).

This finding also higher which qualified from Alert Hospital Addis Ababa 0.71% (Birhualem Taye *et al*., 2014), Jimma Hospital, 3.9% (Teklemariam Zelalem *et al.*, 2013), Adama, Afar and Dire-Dawa 1.5% (Haile-eyesus Adamu and Beyene Petros, 2009), Hiwot Fana Specialized University Hospital 2.2% (Teklemariam Zelalem *et al*., 2013). However, it is lower than reported from Butajira 14.4% (Dereje Gedel *et al.*, 2017), St. Paul Hospitals in Addis Ababa 22.5% (Endeshaw Tokola, 2005), Gambie Higher clinic Bahir Dar city 15.3%

(Abebe Alemu *et al.*, 2011) and Hawassa 12.2% (Shimelis Assefa *et al.*, 2009), Southwest Ethiopia 17% by (Teklemariam Zelalem, *et al.*, 2008).

Cyclospora cayetanensis was the least reported from OIPI, which reason for 2.60% infection rates in this study. The prevalence is comparable to a study conducted from Othona Hospital Wolayita Sodo 2.8% (Menbereleul Mathwose *et al.*, 2014), South western Ethiopian 3.7% (Mohamed Awole *et al.*, 2003). However, it is almost consonant with study in Jimma, Gondar, Addis Ababa 3.9%, 2.7%, 3.1% respectively. However, the prevalence is lowered when compared from Hawassa (0.4%), Eastern Tigray (0.42%). The presence of these emerging opportunistic parasites in this study indicated that HIV infected individuals may accelerates the evolution of AIDS due to the down regulation of the immune system.

This study also tried to articulate some verified and theorized linked factors and showed an indiscriminating OIPI among study participants by age category, sex, level of education, occupation, marital status, and others. However, it revealed that $CD4^+$ status, history of diarrhea, poor adherence to ART treatment, not regularly washing hands after toilet, drinking water from unprotected sources, residing in urban had statistically significant associations with opportunistic intestinal parasitic infections.

This study designated that OIPIs were found significantly associated with participants having < 200 cells/ul count compared to participants with any of $CD4^+$ categories. For instance, having <200 cells/µl count was 49.08 times more likely to be infected by the parasites than those who were had $CD4^+$ cell count above 500 cells/µl (AOR=49.08, 95% CI: 9.440-228.777, P= 0.000). The association of these parasites for lower 200 cells/µl $CD4^+$ count is comparable with studies done in other parts of Ethiopia (Gondar, Dessie, Addis Ababa, and Hawassa). Correspondingly, a previous report from elsewhere in the world suggested that increased frequencies of OIPIs were observed in individuals with $CD4^+$ T-cell count <200 cells/µl. This may be since opportunistic parasites are known to resolve artlessly with immune restoration among HIV/AIDS patients (Gurunathan *et al.*, 2015).

Similarly, individuals who did not have handwashing after the toilet were 10.4 times more likely to be infected with OIPI. Different studies also reported that the prevalence of OIPIs is associated with poor handwashing after defecation. This may due to contamination hand with faces infected by parasites during defecation, so unwashed hands contain a pathogenic

parasite that causes infections. Especially, *Cryptosporidium* species is 10 to 30 oocytes enough to be pathogenic. Handwashing can prevent about 30% of diarrhea-related sicknesses (https://www.cdc.gov/handwashing/why-handwashing.html).

Also, in this finding 75% of study participants had diarrheal history and this can probably be explained by the fact that these patients were had low $CD4^+$ T-cell counts. Another factor may low attention in the adherence to the treatment regimen. The effectiveness of ART highly depends on good compliance with the treatment (Adamu *et al.*, 2020; Clarke & Mcintyre, 2020 & Bangsberg *et al.*, 2001). However; a low level of adherence to the treatment is a common observation in low-income countries. Similarly, in this finding poor adherence to ART drugs had 7.42 times likely happen when compared to good adherence (AOR=7.427, 95% CI 2.488-22.172, P=0.00). Because of this perfect adherence can have a great positive effect on the reconstitution of immune system in HIV infected patients.

In this finding, residents show significant differences. Patients residing in rural (31.8%) 0.197 less likely risky to OIPI when comparing with urban resident (68.2%) (AOR=0.197, 95% CI: 0.053-0.734, P= 0.015). This result contradicts with different studies in different locations. However, it is comparable with the study in Burkina Faso. The tendency of people clustering around the cities (population density) causes a lack of access to clean water and increases anthropogenic factors that increase the pollution of water in urban. The participant's proportion from rural areas is small. This might explain why the lowest prevalence of parasitism was found in rural areas while the highest prevalence in urban areas.

Drinking water from unprotected sources was as a forecaster factor of OIPIs by indicating 76% of parasite positive HIV/AIDS were using from this source. The outcomes also supported by a study done Dessie Ethiopia (Assefa Missaye *et al.*, 2013), Nigeria(Akinbo *et al.*, 2010), and Malaysia (Sim & Lim, 2011).

Some OIP were not detected for the reason that of the non-availability of reagents and the non-applicability of all techniques for *Microsporidia* and molecular methods to distinguish the species of parasites. Hence, the prevalence of OIPI among the study participants might have been underestimated

6 CONCLUSION

The present studies showed that the overall prevalence rate of OIPI was 17.9%. The predominant OIPI were *Cryptosporidium* species followed by *Cystoisospora belil* and *Cyclospora cayetanensis* was identified among study participants. Non-opportunistic intestinal parasites such as *Entamoeba histolytica/dispar, Giardia lamblia, Ascaris lumbricoides*, *hookworms and Trichuris trichiura,* were also identified in ART patients.

This finding also revealed that residence; sources of drinking water, washing hand after toilet, $CD4^+$ T-cell count, the history of diarrhea, ART adherence, and trimming of the fingernail were significantlly associatiated with OIPI. However, there was no significant association of OIPI with sex and age groups.

7 RECOMMENDATIONS

Based on the results of the study the following recommendations are made:

- Routine screening for OIPI should be made especially for all asymptomatic and symptomatic HIV seropositive individuals to administer early and specific treatment should be given to prevent OIPIs among all HIV/AIDS patients.
- Health extension workers in each kebeles of south Gondar zone should mobilize the community by giving health education regarding these parasites transmission and prevention is important to reduce this parasitic infection among peoples living with HIV.
- Boiled or appropriately treated water should be used for drinking purposes especially by those who are immunocompromised.
- This data suggests that non-adherence to ART is associated with an increased incidence of OIPIs. Therefore, clinicians at HIV treatment centers could adopt a policy to systematically follow-up subjects on antibiotics, to ensure timely and adequate treatment of these OIPIs, while simultaneously using other reminder tools to improve adherence to ART.
- Counselling of patients who have poor adherence to ART treatment, Make awareness on environmental and personal hygiene should also be given to HIV-infected individuals.

8 REFERENCES

Abdel-hafeez, E. H., Ahmad, A. K., Ali, B. A., & Moslam, F. A. (2012). Opportunistic Parasites among Immunosuppressed Children in Minia District, *Egypt* **50**(1), 57–62.

Abdourahamane, Y. (2017). Opportunistic and other intestinal parasites infections among HIVpositive patients in the era of combination antiretroviral therapy and preventive treatment in Ouagadougou, Burkina Faso. *Journal of HIV for Clinical and Scientific Research*, 4, 008–014.(https://doi.org/10.17352/2455-3786.000122).

Abebe Alemu., Yitayal Shiferaw., Gebeyaw Getnet., Aregaw Yalew., Zelalem Addis. (2011). Opportunistic and other intestinal parasites among HIV/AIDS patients attending Gambi higher clinic in BahirDar city, North West Ethiopia. *Asian Pacific journal of tropical medicine* **4**(8): 661-665. (https://doi.org/10.1016/S1995-7645 (11)60168-5).

Abebech Yitagesu. (2018*)*. Prevalence of opportunistic intestinal protozoan parasitic Infections and associated risk factors among HIV seropositive individuals at Dilchora referral hospital, Dire Dawa Town, Eastern Ethiopia (master's thesis, Haramaya University).

Adamu, U. S., Archer, W. R., Braka, F., Damisa, E., Siddique, A., Baig, S., Higgins, J., Sume, G. E., Korir, C. K., Gidado, S., Bammeke, P., Forbi, J. C., Burns, C., Liu, H., Jorba, J., Franka, R., Bolu, O., Adamu, U. S., Archer, W. R., Bolu, O. (2020). Weekly epidemiological record Relevé épidémiologique hebdomadaire. 09, 329–344.

Afera Halefom, Asirat Teshome, Ermias Sisay, Imran Ahmad.(2018) Dynamics of Land Use and Land Cover Change Using Remote Sensing and GIS: A Case Study of Debre Tabor Town, South Gondar, Ethiopia. Journal of Geographic Information System, 10, 165-174. (https://doi.org/10.4236/jgis.2018.102008).

Ajjampur, S.S., Rajendran, P, Ramani, S, Banerjee, I, Monica, B, Sankaran, P, *et al*. (2008). Closing the diarrhea diagnostic gap in Indian children by the application of molecular techniques. *Journal of Medical Microbiology*.57:1364-8.

Akinbo, F. O., Okaka, C. E., & Omoregie, R. (2010). Prevalence of intestinal parasitic infections among HIV patients in Benin City, Nigeria. *Libyan Journal of Medicine* **5**(1). (https://doi.org/10.3402/ljm.v5i0.5506).

Alemsegede Messay, Abebe Reda, Girma. Misa .(2015) Prevalence and Associated Risk Factors of Opportunistic Intestinal Parasites among HIV Positive and Negative Individuals in South Ethiopia: A Case Control Study. *Bio Accent Organization* 1: 001.

Almeria, S., Cinar, H. N., & Dubey, J. P. (2019). *Cyclospora cayetanensis* and Cyclosporiasis : An Update. 1-34.

Al-Qobati, S. A., Al-Nabehi, B.A., Mohamad, A.A., Al-Nabbhi, A.S., & Al-Kadi, M.A. (2018). Enteric protozoal infections among immunocompromised and immunocompetent people, Sana'a Town, Yemen. *European Commission Microbiology* 14.12(1): 837-843.

Amatya, R., Shrestha, R., Poudyal, N., & Bhandari, S. (2011). Opportunistic intestinal parasites and CD4 count in HIV infected people. *Journal of the pathology of Nepal* **1(**2), 118-121.

Anane, S, & Attouchi, H. (2010). Microsporidiosis: Epidemiology, clinical data and therapy. *Gastroentérologi Cliniqu Biologique,* **34**(8–9), 450–464.

and *Cyclospora cayetanensis* infections among people living in a slum area in Kathmandu valley, Nepal. *Biomedical Center Research Notes*, 1–5. (https://doi.org/10.1186/s13104-017-2779-2)

Andrian, N., Acikgoz, Z. C., Turkay, S & Andiran, F. (2006). *Blastocystis hominis an* emerging and imitating cause of acute abdomen in children. *Journal of pediatric surgery* **41**(8): 1489-1491.

Anglaret, X., Chêne, G., Attia, A., Toure, S., Lafont, S., Combe, P., & Manlan, K. (1999). Early chemoprophylaxis with trimethoprim-sulphamethoxazole for HIV-1-infected adults in Abidjan , Côte d ' Ivoire : a randomised trial. **353**, 1463–1468.

Anteneh Mulat. (2012). Prevalence of *Cryptosporidium* species infection among HIV positive persons with and without antiretroviral treatment in Nekemte Hospital M.sc Thesis.

Article, M., Hewitt, R. G., Yiannoutsos, C. T., Higgs, E. S., Carey, J. T., Geiseler, P. J., Soave, R., Rosenberg, R., Vazquez, G. J., Wheat, L. J., Fass, R. J., Antoninievic, Z., Walawander, A. L., & Flanigan, T. P. (2000). Paromomycin : No More Effective than Placebo for Treatment of Cryptosporidiosis in Patients with Advanced Human Immunodeficiency Virus Infection, 1084-1092.

Assefa Missaye., Mulat Dagnew., Abebe Alemu, & Agersew Alemu. (2013). Prevalence of intestinal parasites and associated risk factors among HIV/AIDS patients with pre-ART and on-ART attending Dessie hospital ART clinic, Northeast Ethiopia. *AIDS research and therapy*: **10**(1).

Avilés, J., Yombi, J.C., Erostegui, C., Torrico, M, Rojas M., *et al*. (2020) Prevalence of Opportunistic and Non-Opportunistic Intestinal Parasites in HIV/AIDS Patients in Cochabamba, Bolivia. Current HIV Research: CRHA-120. DOI: 10.29011/2575-7105.100120.

Ayeh-Kumi, F., Quarcoo, S., Kwakye-Nuako, G., Kretchy, P., Osafo-Kantanka, A, & Mortu, S. (2009). Prevalence of Intestinal Parasitic Infections among Food Vendors in Accra, Ghana. *Journal Tropical medicine and parasitology***32**:1-8.

Babu, A.R, Chandra, T.J, Kumari, R.L. (2017). Correlation between stool microscopy and level of immunosuppression in HIV/AIDS patients with diarrhea in East Godavari District, Andhra Pradesh. *Indian Journal Microbiol Research* **4**(4):416-418.

Bachur TP, Vale JM, Coelho IC, Queiroz TR, Chaves Cde S (2008) Enteric parasitic infections in HIV/AIDS patients before and after the highly active antiretroviral therapy. The Brazilian journal of infectious diseases : an official publication of the Brazilian Society of Infectious Diseases **12**: 115-122.

Barcelos, N. B., e Silva, L. de F., Dias, R. F. G., de Menezes Filho, H. R., & Rodrigues, R. M. (2018). Opportunistic and non-opportunistic intestinal parasites in HIV/ AIDS patients in relation to their clinical and epidemiological status in a specialized medical service in Goiás, Brazil. Revista Do Instituto de Medicina Tropical de Sao Paulo, 60(September 2017), 1–9. (https://doi.org/10.1590/S1678-9946201860013).

Basnet, A., Sherchan, B., Rijal, B., Sharma, S., & Khadga, P. (2010). Detection of Coccidian Parasites and their Clinical Manifestation, Treatment and Prophylaxis in HIV Infected Patients in Tribhuvan University Teaching Hospital. Scientific World **8**(8), 51–55. (https://doi.org/10.3126/sw.v8i8.3849).

Belete, H, & Kloos, H. (2005). Intestinal parasitism. In Epidemiology and ecology of health and disease in Ethiopia. Shama Books, Addis Ababa, Ethiopia 518-538.

Bentwich, R. (1998). Decreased CD4 and increased CD8 counts with T cell activation are associated with chronic helminth Infections. *Clinical & Experimental Immunology* **114**(3): 414-421.

Bhattachan, B., Sherch, J. B., Tandukar, S., & Dhoubhadel, B. G. (2017). Detection of *Cryptosporidium parvum*.

Brooker, S., Rowlands, M., Haller, L., Savioli, L., & Bundy, D. A. P. (2000). Towards an atlas of human helminth infection in sub-Saharan Africa: The use of geographical

information systems (GIS). *Parasitology Today* **16**(7), 303–307. Retrieved from (https://doi.org/10.1016/S0169-4758 (00)01687-2).

Buchacz, K., Lau, B., Jin, Y., Bosch, R., Abraham, A. G., Gill, M. J, & Martin, J. N. (2016). Incidence of AIDS-defining opportunistic infections in a multicohort analysis of HIV-infected persons in the United States and Canada, 2000–2010. *The Journal of infectious diseases* **214**(6), 862-872.

Cabada, M. M., & White Jr, A. C. (2010). Treatment of cryptosporidiosis: do we know what we think we know? *Current opinion in infectious diseases* **23**(5), 494-499.

Cama, V. A., Ross, J. M., Crawford, S., Kawai, V., Chavez-Valdez, R., Vargas, D and Ortega, Y. (2007). Differences in clinical manifestations among *Cryptosporidium* species and subtypes in HIV-infected persons. *The Journal of infectious diseases*, **196**(5), 684-691.

Care, P., Gebre, B., Alemayehu, T., Girma, M., Ayalew, F., Tadesse, B. T., & Shemelis, T. (2019). Cryptosporidiosis and Other Intestinal Parasitic Infections and Concomitant Threats Among HIV-Infected Children In Southern Ethiopia Receiving First-Line Antiretroviral Therapy.

Casillas, S. M., Hall, R. L., & Herwaldt, B. L. (2019). Cyclosporiasis Surveillance - United States, 2011-2015. Morbidity and Mortality Weekly Report. Surveillance Summaries (Washington, D.C. : 2002), **68**(3), 1–16. (https://doi.org/10.15585/mmwr.ss6803a1).

Cassano, N., Scoppio, B.M., Loviglio, M.C, and Vena, G.A. (2005). Remission of delayed pressure urticaria after eradication of *Blastocystis hominis*. *Acta Dermato-Venereologica* **85**: 357-358.

Centers for Disease Control and Prevention. (2018). Epidemiology and Risk Factors. Retrieved November 10, 2019, https://www.cdc.gov/parasites/strongyloides/epi.html.

Centers for Disease Control and Prevention. (2019). *Amebiasis*. Retrieved December 10, 2019, from https://www.cdc.gov/pasirates/amebiasis/pathogen.html.

Centers for Disease Control and Prevention. (2019). *Blastocystis*. Retrieved December 10, 2019, from https://www.cdc.gov/dpdx/blastocystis/index.html.

Centers for Disease Control and Prevention. (2019). *Blastocytis hominss*. Retrieved December 10, 2019, from https://www.cdc.gov/dpdx/microsporidiosis/.

Centers for Disease Control and Prevention. (2019). *Cryptosporidiosis*. Retrieved December 10, 2019, from https://www.cdc.gov/dpdx/cryptosporidiosis/index.html.

Centers for Disease Control and Prevention. (2019). *Cyclosporiasis*. Retrieved December 10, 2019, from https://www.cdc.gov/parasites/cyclosporiasis/biology.html.

Centers for Disease Control and Prevention. (2019). *Cystoisospora*. Retrieved December 10, 2019, from https://www.cdc.gov/parasites/cystoisospora/biology.html.

Centers for Disease Control and Prevention. (2019). *Microsporidiosis*. Retrieved December 10, 2019, from https://www.cdc.gov/dpdx/microsporidiosis.

Certad, G. (2003). Isosporiasis in Venezuelan adults infected with human immunodeficiency virus: clinical characterization. *American Journal of Tropical Medicine Hygiene* **69**: 217–222.

Certad, G., Arenas-pinto, A., Pocaterra, L., Ferrara, G., Castro, J., Bello, A., Nu, L. U. Z., Parasitología, C. De, Vargas, E. D. M. J. M., & Jose, S. (2003). *Isosporiasis* in venezuelan adults infected with human immunodeficiency virus: *clinical characterization.* **69**(2), 217–222.

Chacín-bonilla, L. (2010). Acta Tropica Epidemiology of *Cyclospora cayetanensis*: A Review focusing in endemic area. 115, 181-193.

Chappell, C.L., Okhuysen, P.C., Langer-Curry, R., Widmer, G., Akiyoshi, D.E., Tanriverdi, S., *et al*. *Cryptosporidium hominis*: experimental challenge of healthy adults. *American Journal of Tropical Medicine and Hygiene*. 2006 Nov **75** (5):851-7.

Charan, J, & Biswas, T. (2013). How to calculate sample size for different study designs in medical research? *Indian journal of psychological medicine* **35**(2): 121–126.doi:10.4103/0253-7176.116232.

Checkley, W., White Jr, A. C., Jaganath, D., Arrowood, M. J., Chalmers, R. M., Chen, X. M., and Huston, C. D. (2015). A review of the global burden, novel diagnostics, therapeutics, and vaccine targets for *Cryptosporidium*. *The Lancet Infectious Diseases* **15**(1), 85-94.

Chen, S., Lalazar, G., Barak, O., Adar, T., Doviner, V., & Mizrahi, M. (2012). Protein-loosing entropathy induced by unique combination of CMV and HP in an immunocompetent patient. Case Reports in Medicine, 2012, 0–4. (https://doi.org/10.1155/2012/361892).

Chen, Y., Zhang, Y., Yang, B., Qi, T, & Lu, H. (2007). Short report: Seroprevalence of *Entamoeba histolytica* Infections in HIV-infected patients in China. *American Journal of Tropical Medicine and Hygiene* **77**(5): 825-828.

Chen, C.H., Sun, H.Y., Chien, H.F., Lai, H.S.S, & Chou, N.K. (2014). *Blastocystis hominis* infection in a post-cardiotomy patient on extracorporeal membrane oxygenation support: A case report and literature review *International Journal of Surgery Case Reports* **5**(9): 637-639. 49.

Cimerman, S., Infectologia, I. De, Ribas, E., Federal, U., & Paulo, D. S. (1999). Enteric parasites and AIDS. **117**(6), 266–273.

Clark, C. G., van der Giezen, M., Alfellani, M. A., & Stensvold, C. R. (2013). Recent Developments in Blastocystis *Research. Advances in Parasitology*, 1–32. doiI:10.1016/b978-0-12-407706-5.00001-0.

Clarke, S. C., & Mcintyre, M. (2020). The incidence of *Cyclospora cayetanensis* in stool samples submitted to a district general hospital. 1996, 189–193.

Clinical Reevaluation. (2007). December 2013.

Curtis, V. and Cairncross, S. **(**2003**).** Effect of washing hands with soap on diarrhea risk in the community: a systematic review. *Lancet Infect Dis* 3, 275–281.

Da Silva, C. V., Ferreira, M. S., Borges, A. S., & Costa-Cruz, J. M. (2005). Intestinal parasitic infections in HIV/AIDS patients: experience at a teaching hospital in central Brazil. Scandinavian. *Journal of Infectious Diseases* **37**(3), 211-215.

Danchaivijitr, S., Dhiraputra, C., Santiprasitkul, S., & Judaeng, T. (2005). Prevalence and impacts of nosocomial infection in Thailand in 2001. *Journal of the Medical Association of Thailand* **88**(Suppl 10), S1 9.

Daniel Gebretsadik., Haftay Haileslasie & Daniel Feleke. (2018). Intestinal parasitosis among HIV/AIDS patients who are on anti-retroviral therapy in Kombolcha, North Central, Ethiopia: A cross-sectional study. *Biomedical Center Research Notes*, **11**(1), 1–5. (https://doi.org/10.1186/s13104-018-3726-6).

Dawit Ayalew, Endashaw Tekela, & Beyene Petros. (2008). *Cryptosporidium* and Giardia Infections and drinking water sources among children in Lege Dini, Ethiopia. *Tropical medicine and international healt*h **13**(4): 472-476.

DeHovitz, J. A., Pape, J. W., Boncy, M., and Johnson J, W. D. (1986). Clinical manifestations and therapy of *Isospora belli* infection in patients with the acquired immunodeficiency syndrome. *New England Journal of Medicine* **315**(2), 87-90.

Dereje Gedle., Gemechu Kumera., Tewodros Eshete., Kasahun Ketema., Haweni Adugna, & Fetuma Feyera. (2017). Intestinal parasitic Infections and its association withundernutrition and CD4 T cell levels among HIV/AIDS patients on HAART in Butjira, *Ethiopia.Journal of Health, Population, and Nutrition* **36**(1). doi: 10.1186/s41043-017-0092-2.

Dillingham, R. A., Pinkerton, R., Leger, P., Severe, P., Guerrant, R. L., Pape, J. W., & Fitzgerald, D. W. (2009). High Early Mortality in Patients with Chronic Acquired Immunodeficiency Syndrome Diarrhea Initiating Antiretroviral Therapy in Haiti : A Case-Control Study **80**(January 2007), 1060–1064.

Dinku Senbeta., Lemlem Gebremariam, & Guesh Gebremariam. (2017). Intestinal Parasitic Infections among HIV/AIDS Infected and Noninfected Patients in Eastern Tigray, northern Ethiopia. *European journal of pharmaceutical and medical research* **4**(4): 617- 625.

Doherty, T. (2002). Principles and practice of clinical parasitology**. In Transactions of the Royal Society of Tropical Medicine and Hygiene** (Vol. 96, Issue 4). (https://doi.org/10.1016/s0035-9203(02)90401-3).

Doyle, P. W., Helgason, M. M., Mathias, R. G., & Proctor, E. M. (1990). Epidemiology and Pathogenicity of Blastocystis hominis **28**(1), 116–121.

Echoru, I., Herman, L., Micheni, L., & Ajagun-ogunleye, M. O. (2015). Epidemiology of Coccidian Parasites in HIV Patients of Northern Uganda. 7(December 2014), 904–913. (https://doi.org/10.9734/BJMMR/2015/15959).

Endeshaw Tokola. (2005). Opportunistic and other intestinal parasites among HIV/AIDS patients in Ethiopia. Ph.D. dissertation paper 1-123.

Faisal, A.F., Bokhar, A.A. *Microsporidium*. [Updated 2020 Jan 30]. In: StatPearls [Internet]. Treasure Island (FL): StatPearls Publishing; 2020 Jan-. Available from: (https://www.ncbi.nlm.nih.gov/books/NBK537166).

Faye, B., Tine, R. C., Ndiaye, J. L., Kintega, C., Manga, N. M., Sow, P. S., & Gaye, O. (2010). Impact of intestinal parasites on the intensity of HIV Infections in Senegal. *Journal of Antvirals and Antiretrovirals* **2**(1): 11-12.

Fayer, R., Speer, C. A & Dubey, J. P. (2018). General biology of *Cryptosporidium*. In *Cryptosporidiosis of man and animals* (pp. 1-30). CRC press.

Feyisayo Jegede, E., Ibijoke Oyeyi, E. T., Hamisu Bichi, A., Akwen Mbah, H., & Torpey, K. (2014). Prevalence of intestinal parasites among HIV/AIDS patients attending Infectious Disease Hospital Kano, Nigeria. Pan African Medical Journal, 17, 2–8. (https://doi.org/10.11604/pamj.2014.17.295.3707)

Fichtenbaum, C. J., Zackin, R., & Feinberg, J. (2000). Rifabutin but not clarithromycin prevents cryptosporidiosis in persons with advanced HIV infection. February, 2889–2893.

Fithamlak Bistegen, Banchalem Nega , Hailu Chare , Efrata Girma, Tezera Moshago, & Mahlet Admasu. (2018). Spectrum of opportunistic infections and associated factors among people living with HIV/AIDS in the era of highly active anti-retroviral treatment in Dawro Zone hospital: A retrospective study. *Biomedical Center Research Notes*, **11**(1), 1–7. (https://doi.org/10.1186/s13104-018-3707-9)

Fletcher, S. M., Stark, D., & Ellis, J. (2011). Prevalence of gastrointestinal pathogens in Sub-Saharan Africa: systematic review and meta-analysis.

Franzen, C., & Müller, A. (2001). Microsporidiosis: Human diseases and diagnosis. Microbes and Infection, 3(5), 389–400.(https://doi.org/10.1016/S1286-4579(01)01395-8)

Fryauff, D. J., Krippner, R., Prodjodipuro, P., Ewald, C., Kawengian, S., Pegelow, K., & Gross, R. (1999). *Cyclospora cayetanensis* among expatriate and indigenous populations of West Java, Indonesia. *Emerging infectious diseases* **5**(4), 585.

Garcia, L. S., Arrowood, M., Kokoskin, E., Paltridge, G. P., Pillai, D. R., Procop, G. W., & Visvesvara, G. (2018). Laboratory diagnosis of parasites from the gastrointestinal tract. *Clinical microbiology reviews* **31**(1): e00025-17.

Gathe, J.r, J. C., Mayberry, C., Clemmons, J., & Nemecek, J. (2008). Resolution of severe cryptosporidial diarrhea with rifaximin in patients with AIDS. *JAIDS Journal of Acquired Immune Deficiency Syndromes* **48**(3), 363-364.

Getaneh Alemu., Dagninet Alelign & Ashenafi Abossie. (2018). Prevalence of Opportunistic Intestinal Parasites and Associated Factors among HIV Patients while Receiving ART at Arba Minch Hospital in Southern Ethiopia: A Cross-sectional Study. *Ethiopian Journal of Health Sciences* **28**(2), 147–156.

Getaneh Amed., Medhin Girmy, & Shimelis Techalew. (2010). *Cryptosporidium* and Strongyloides stercoralis Infections among people with and without HIV Infections and efficiency of diagnostic methods for Strongyloides in Yirgalem Hospital, southern Ethiopia. *Biomedical Center research notes* **3**(1): 90.

Ghoyounchi, R., Ahmadpour, E., Spotin, A., Mahami-Oskouei, M., Rezamand, A., Aminisani, N., Ghojazadeh, M., Berahmat, R., & Mikaeili-Galeh, T. (2017). Microsporidiosis in Iran: A systematic review and meta-analysis. *Asian Pacific Journal of Tropical Medicine* **10**(4), 341–350. https://doi.org/10.1016/j.apjtm.2017.03.017.

Gillespie, S.H. (2001). Intestinal nematodes Principles and Practice of Clinical Parasitology. Chichester: John Wiley and Sons 561-83.

Goodgame, W.R. (1996). Understanding Intestinal spore-forming protozoa: *Cryptosporidia*, *Microsporidia*, *Isospora*, *Cyclospora*. *Ann. International Medicine* **124**: 429-441.

Gracia, L. S, & Bruckner, D.A. (1997). Diagnostic Medical Parasitology AdvancesI Parasitology **40**:38-72.

Gruenberg K and Guglielmo B. (2019). Anti-Infective Chemotherapeutic & Antibiotic Agents. In: Papadakis MA, McPhee SJ, Rabow M. eds. Current Medical Diagnosis & Treatment. New York, NY: McGraw-Hill.

Guiguet, M., Furco, A., Tattevin, P., Costagliola, D., Molina, J., Database, H., Umr, M. C., & Inserm, U. (2007). HIV-associated *Isospora belli* infection: incidence and risk factors in the French Hospital Database on HIV 124–130.

Gurunathan, S., Habib, R. El, Baglyos, L., Meric, C., Plotkin, S., Dodet, B., Corey, L., & Tartaglia, J. (2015). Use of predictive markers of HIV disease progression in vaccine trials **27**(2009), 1997–2015. https://doi.org/10.1016/j.vaccine.2009.01.039.

Habtom Kiros., Endalekachew Nibret, Abyainh Munshea., Bizuayehu Kerisew & Melaku Adal. (2015). Prevalence of intestinal protozoan infections among individuals living with HIV/AIDS at Felegehiwot Referral Hospital, Bahir Dar, Ethiopia. *International Journal of Infectious Diseases* **35** (80–86).

Hafiz Ahmad.(2018). Opportunistic intestinal parasitic infections in the immunocomprised HIV/AIDS patients, RAK-Medical and Health Sciences University, UAE Dubai

Haileeyesus Adamu, Teklu Wegayehu & Beyene Petros. (2013). High Prevalence of Diarrhoegenic Intestinal Parasite Infections among Non-ART HIV Patients in Fitch Hospital, Ethiopia. PLoS One Journal **8**(8):726-734.

Haileeysus Adamu, & Beyne Petros. (2009). Intestinal protozoan Infections among HIV positive persons with and without Antiretroviral Treatment (ART) in selected ART centers in Adama, Afar and Dire-Dawa, Ethiopia. *Ethiopian Journal of Health Development* **23**(2).

Hailemariam Getachew., Kassu Afewerk., Abebe Gemeda., Abate Ebba., Damite Demkech., Mekonnen Endris, & Ota Fusao. (2004). Intestinal Parasitic Infections in HIV/AIDS and HIV Seronegative Individuals in a Teaching Hospital, Ethiopia. *Journal of Infectious Diseases* **57**:41-43.

Harms, G & Feldmeier, H. (2002). HIV infection and tropical parasitic diseases - deleterious interactions in both directions. *Tropical medicine & international health*: TM & IH. 7.

479-88. 10.1046/j.1365-3156.2002.00893.

Hoge, C. W., Echeverria, P., Shlim, D. R., Rajah, R., Shear, M., Rabold, J. G., & Triplett, J. (1993). Epidemiology of diarrhoeal illness associated with coccidian-like organism among travellers and foreign residents in Nepal. *The Lancet* **341**(8854), 1175-1179.

Holmberg, S. D., Moorman, A. C., Bargen, J. C. Von, Palella, F. J., Loveless, M. O., Ward, D. J., & Navin, T. R. (1998). Possible Effectiveness of Clarithromycin and Rifabutin for Cryptosporidiosis Chemoprophylaxis in HIV Disease. **279**(5), 384–386.

Horne, S., Sibal, B., Sibal, N., and Green, H. K. (2017). *Cryptosporidium* outbreaks: identification, diagnosis, and management. *British Journal of General Practice* **67**(662), 425-426.

http://accessmedicine.mhmedical.com/content.aspx?bookid=2449&sectionid=194442959Accessed February 09, 2020.

https://www.jstor.org/stable/4454841 Human Microsporidiosis and AIDS. **11**(2), 203–207.

Huang, D. B., & Zhou, J. (2007). Effect of intensive handwashing in the prevention of diarrhoeal illness among patients with AIDS: a randomized controlled study Printed in Great Britain. 659-663. https://doi.org/10.1099/jmm.0.46867-0.

Jegede, E. F., Oyeyi, E. T. I., Bichi, A. H., Mbah, H. A, & Torpey, K. (2014). Prevalence of intestinal parasites among HIV/AIDS patients attending Infectious Disease Hospital Kano, Nigeria. *The Pan African medical journal* **17**.

Jelinek, T., Peyeri G., Loscher T., von Sonnenburg F., M.D. Nothdurtt, 1997. The role of *Blastocystis hominis* as a possible intestinal pathogen in travellers. *Journal of Infection*. **35**: 63-66.

Keusch, G.T., Hamer, D., Joe, A., Kelley, M., Griffiths, J, & Ward, H. (1995) "*Cryptosporidia*--who is at risk?" Schweiz Med Wochenschr **125** (18): 899-908.

Khalil, S., Mirdha, B. R., Sinha, S., Panda, A., Singh, Y., Joseph, A., & Deb, M. (2015). Intestinal parasitosis in relation to anti-retroviral therapy, CD4+ T-cell count and diarrhea in HIV patients. *Korean Journal of Parasitology* **53**(6), 705–712. (https://doi.org/10.3347/kjp.2015.53.6.705).

Klassen-fischer, M. K., Neafie, R. C., Wear, D. J., & Meyers, W. M. (2008). Cryptosporidiosis, Isosporiasis, Cyclosporiasis & Sarcocystosis.

Kłudkowska, M., Pielok, Ł., Frąckowiak, K., & Paul, M. (2017). Intestinal coccidian parasites as an underestimated cause of travellers diarrhoea in Polish immunocompetent patients **62**(3), 630–638. https://doi.org/10.1515/ap-2017-0077.

Knobloch, r., zelck, u. E., bialek, r., binder, n., & dietz, k. (2002). Comparison of autofluorescence and iodine staining for detection of *Isospora belli* in feces **67**(3), 304–305.

Laksemi, D.A, Suwanti, L.T, Mufasirin. M, Suastika K, Sudarmaja M. (2020). Opportunistic parasitic infections in patients with human immunodeficiency virus/acquired immunodeficiency syndrome: *A review*, *Veterinary World* **13**(4): 716-725.

Lindsay, D. S., Dubey, J. P., & Blagburn, B. L. (1997). Biology of Isospora spp. from humans, nonhuman primates, and domestic animals. *Clinical Microbiology Reviews* **10**(1), 19–34. https://doi.org/10.1128/cmr.10.1.19.

Lobo, M.L, Xiao L, Antunes F, Matos.O. (2012). Microsporidia as emerging pathogens and the implication for public health: a 10-year study on HIV-positive and -negative patients. *International Journal for Parasitology***42**(2):197-205.

Maggi, P, Larocca, A. M. V, Quarto, M., Serio, G., Brandonisio, O., Angarano, G., & Pastore, G. (2000). Effect of Antiretroviral Therapy on Cryptosporidiosis and Microsporidiosis in Patients Infected with Human Immunodeficiency Virus Type 1. 213–214.

Maggi, Paolo, Larocca, A. M. V., Ladisa, N., Carbonara, S., Brandonisio, O., Angarano, G., & Pastore, G. (2001). Opportunistic Parasitic Infections of the Intestinal Tract in the Era of Highly Active Antiretroviral Therapy: Is the CD4 + Count So Important? *Clinical Infectious Diseases*, 33(9), 1609–1611. (https://doi.org/10.1086/323017)

Maizels, Rick. (2009). Parasite immunomodulation and polymorphisms of the immune system. Journal of biology **8**(6). Doi.org/10.1186/jbiol166.

Mansfield, L. S., & Gajadhar, A. A. (2004). Cyclospora cayetanensis, a food- and waterborne coccidian parasite. *Veterinary Parasitology* **126**(1-2 SPEC.ISS.), 73–90. (https://doi.org/10.1016/j.vetpar.2004.09.011)

Marcos, Luis & Gotuzzo, Eduardo. (2013). Intestinal protozoan infections in the immunocompromised host. Current opinion in infectious diseases. **26**(302-308).

Mazigo.D., Waihenya. R., Lwambo. J., Mnyone. L., Mahande. M., Seni. J., Zinga. M., Kapesa. A., Kweka. J., Mshana. E., Heukelbach. J and Mkoji M. (2010). Co-infections with Plasmodium falciparum, *Schistosoma mansoni* and intestinal helminths among schoolchildren in endemic areas of northwestern Tanzania. Parasites & Vectors 3:44.

Mbuh.V., Ntonifor. N., & Ojong T. (2010) the incidence, intensity and host morbidity of human parasitic protozoan Infections in gastrointestinal disorder outpatients in Buea Sub Division, *Cameroon. Journal Infec Developing Countries* **4**: 38-43.

Mehdi Mohebali, Yonas Yimam & Ambachew Woreta (2020): *Cryptosporidium* infection among people living with HIV/AIDS in Ethiopia: a systematic review and meta-analysis, Pathogens and Global Health. doi: 10.1080/20477724.2020.1746888.

Mekonnen Girma, Wondu Teshome, Beyene Petros and Tekola Endeshaw (2014). Cryptosporidiosis and Isosporiasis among HIV-positive individuals in south Ethiopia : a cross sectional study. 1–6.

Michiels, J. F., Hofman, P., Bernard, E., Saint Paul, M. C., Boissy, C., Mondain. V, & Loubiere, R. (1994). Intestinal and extraintestinal *Isospora belli* infection in an AIDS patient: a second case report. *Pathology-Research and Practice*, **190**(11), 1089-1093.

Missaye, A., Dagnew, M., Alemu, A., & Alemu, A. (2013). Prevalence of intestinal parasites and associated risk factors among HIV/AIDS patients with pre-ART and on-ART attending dessie hospital ART clinic, Northeast Ethiopia. AIDS Research and Therapy, 10(1), 0–9. (https://doi.org/10.1186/1742-6405-10-7)

Mohamed Awole., Solomon Gebre-Selassie., Tesfaye Kassa, & Gebre Kibru. (2003). Prevalence of Intestinal parasites in HIV infected adult patients in South Western Ethiopia. *Journal of Health Development* **17**(1):71 – 78.

Muktar Abadiga. (2019). Adherence to antiretroviral therapy and associated factors among hiv positive adults attending treatment at Nekemte Referral Hospital, West Ethiopia, *Research square*.

Nader, J.L, Mathers, T.C., Ward, B.J, Pachebat, J.A, Swain, M.T, Robinson G, *et al*. (2019). Evolutionary genomics of anthroponosis in *Cryptosporidium*. *Nature Microbiology*. **4** (5):826-836.

Namaji, M.S, Pathan, S.H,& Balki, A.M. (2020). Profile of intestinal parasitic infections in human immunodeficiency virus/acquired immunodeficiency syndrome patients in Northeast India. *Indian Journal of Sexual transmitted diseases and AIDS* **41** 93-6.

Naseer, M., Dailey, F. E., Al Juboori, A., Samiullah, S., & Tahan, V. (2018). Epidemiology, determinants, and management of AIDS cholangiopathy: A review. *World journal of gastroenterology* **24**(7), 76.

National committee for clinical laboratory standards (NCCLS). 1997. Procedures for the Recovery and Identification of Parasites from the Intestinal Tract, Approved 300

Emerging Protozoan Pathogens Guideline, M28-A. National Committee for Clinical Laboratory Standards, Villanova. *Clinical Journal of Parasitology*, 122:573-595.

National Institutes of Health. (2019). Guidelines for the Prevention and Treatment of Opportunistic Infections in Adults and Adolescents with HIV. *AIDS information*, 2020. (https://aidsinfo.nih.gov/contentfiles/lvguidelines/AdultOITablesOnly.pdf)

National Institutes of Health. (2019). Guidelines for the Prevention and Treatment of Opportunistic Infections in Adults and Adolescents with HIV. AIDSinfo, 2020. (https://aidsinfo.nih.gov/contentfiles/lvguidelines/AdultOITablesOnly.pdf)

Ngum, N. H., Ngum, A. N., & Jini, S. S. (2015). Opportunistic intestinal protozoan infections in HIV/AIDS patients on antiretroviral therapy in the North West Region of Cameroon. *Microbiology Research Journal International*, 269-275.

Nigro, L., Larocca, L., Massarelli, L., Patamia, I., Minniti, S., Palermo, F., & Cacopardo, B. (2003). A placebo-controlled treatment trial of *Blastocystis hominis* Infections with metronidazole, *Journal of Travel Medicine***10**: 128–130.

Niguse Telele. (2010). Intestinal Parasitic Infections among HIV Seropositive and Seronegative Adult Patients Presented with Diarrhoea in Gondar , Northwest.

Nissapatorn, V., & Sawangjaroen, N. (2011). Parasitic infections in HIV infected individuals: Diagnostic & therapeutic challenges. Indian Journal of Medical Research, **134**(12), 878–897. (https://doi.org/10.4103/0971-5916.92633)

Noor, R., Saha, S. R., Rahman, F., Munshi, S. K., Uddin, M. A., & Rahman, M. M. (2012). Frequency of opportunistic and other intestinal parasitic infections in patients infected with human immunodeficiency virus in Bangladesh. *Tzu Chi Medical Journal*, **24**(4), 191-195.

Nsagha, D. S., Njunda, A. L., Assob, N. J. C., Ayima, C. W., Tanue, E. A., kibu, O. D., & Kwenti, T. E. (2016). Intestinal parasitic infections in relation to CD4+ T cell counts and diarrhea in HIV/AIDS patients with or without antiretroviral therapy in Cameroon. *Biomedical Center Infectious Diseases*, ***16***(1), 1–10. (https://doi.org/10.1186/s12879-016-1337-1)

Nsagha, D. S., Njunda, L. A., Assob, N. J. C., Ayima, C. W., Tanue, E. A., Kibu, O. D., & Kwenti, T. E. (2017). Prevalence and Predisposing Factors to Intestinal Parasitic Infections in HIV/AIDS Patients in Fako Division of Cameroon. *American Journal of Epidemiology* **5**(3): 42-49.

Oddó, D., Méndez, G. P., Retamal, Y., & Oddó, A. (2018). Intestinal isosporiasis in patients with acquired immunodeficiency syndrome (AIDS). Pathologic diagnosis in small intestinal mucosal biopsies. Annals of Diagnostic Pathology **33**(17–22). (https://doi.org/10.1016/j.anndiagpath.2017.11.002)

Okhuysen, P.C., Chappell, C.L., Crabb, J.H., Sterling, C.R., DuPont, H.L. Virulence of three distinct *Cryptosporidium parvum* isolates for healthy adults. *Journal of Infectious Diseases*. 1999 Oct. **180** (4):1275-81.

Okodua M, Adeyeba OA, Tatfeng YM, Okpala HO. (2003). Age, & Sex Distribution of Intestinal Parasitic Infection among HIV Infected Subjects in Abeokuta, Nigeria. *Online J Health Allied Scs* 4:3.

Ortega, Y. R., Nagle, R., Gilman, R. H., Watanabe, J., Miyagui, J., Quispe, H., Kanagusuku, P., Roxas, C., & Sterling, C. R. (1997). Pathologic and Clinical Findings in Patients with Cyclosporiasis and a Description of Intracellular Parasite Life- Cycle Stages. *The Journal of Infectious Diseases* **176**(6), 1584–1589.
Retrieved from https://doi.org/10.1086/514158.

Ortega, Y. R., Nagle, R., Gilman, R. H., Watanabe, J., The, S., Diseases, I., Dec, N., Ortega, Y. R., Nagle, R., Gilman, R. H., Watanabe, J., Miyagui, J., Quispe, H., Kanagusuku, P., Roxas, C., & Sterling, C. R. (2020). Pathologic and Clinical Findings in Patients

with Cyclosporiasis and a Description of Intracellular Parasite Life-Cycle Stages Miyagui, Hugo Quispe , Patricia Kanagusuku , Concepcion Roxas and Charles R . Sterling Published by: Oxford University Press S. **176**(6), 1584–1589.

Ortega, Y., & R. Adam, 1997. Giardia: an overview and update. *Clinical Infectious Diseases* **25**(3):545549.

Pape, J. W. (1994). *Cyclospora* Infection in Adults Infected with HIV: Clinical Manifestations, Treatment, and Prophylaxis. *Annals of Internal Medicine* **121**(9), 654. doi:10.7326/0003-4819-121-9-199411010-00004.

Pavie, J., Menotti, J., Porcher, R., Donay, J. L., Gallien, S., Sarfati, C., Derouin, F., & Molina, J. M. (2012). Prevalence of opportunistic intestinal parasitic infections among HIV-infected patients with low CD4 cells counts in France in the combination antiretroviral therapy era. International Journal of Infectious Diseases, 16(9), 677–679. (https://doi.org/10.1016/j.ijid.2012.05.1022)

Rao, R. P. (2016). Study of Opportunistic Intestinal Parasitic Infections in HIV Seropositive Patients at a Tertiary Care Teaching Hospital in. *International Journal of Contemporary Medical Research* **3**(8), 2219–2222.

Reina, F. T. R., Ribeiro, C. A., de Araújo, R. S., Matté, M. H., Castanho, R. E. P., Tanaka, I. I., Viggiani, A. M. F. S., & Martins, L. P. A. (2016). Intestinal and pulmonary infection by *cryptosporidium parvum* in two patients with HIV/AIDS. Revista Do Instituto de Medicina Tropical de Sao Paulo **58**(2), 2–5.
Retrieved from https://doi.org/10.1590/S1678-9946201658021.

Rodriguez-Morales, A. J., and Castañeda-Hernández, D. M. (2019). Protozoa: *Cystoisospora belli* (Syn. *Isospora belli*).

Rodríguez-Pérez, E. G., Arce-Mendoza, A. Y., Montes-Zapata, É. I., Limón, A., Rodríguez, L. É., & Escandón-Vargas, K. (2019). Opportunistic intestinal parasites in immunocompromised patients from a tertiary hospital in monterrey, mexico. *Infezioni in Medicina* **27**(2), 168–174.

Roellig, D. M., Yoder, J. S., Madison-Antenucci, S., Robinson, T. J., Van, T. T., Collier, S. A., Shea, S. (2017). Community laboratory testing for *Cryptosporidium*: multicenter study retesting public health surveillance stool samples positive for *Cryptosporidium* by rapid cartridge assay with direct fluorescent antibody testing PloS one **12**(1).

Rossignol, J. (2006). Nitazoxanide in the treatment of acquired immune deficiency syndrome-related cryptosporidiosis: results of the United States compassionate use program in 365 patients. June, 887–894.
Retrived from https://doi.org/10.1111/j.1365-2036.2006.03033.x.

Ryan, U., Paparini, A., & Oskam, C. (2017). New Technologies for Detection of Enteric Parasites. In Trends in Parasitology.(https://doi.org/10.1016/j.pt.2017.03.005)

Sadaf, H.S., Khan, S.S., Urooj, K.S., Asma, B., & Ajmal, S.M. (2013) Blastocystis hominis-potential diarrheal agent: *A review. International Research Journal of Pharmaceutical* **4**(1): 1-5. 48.

Sangaré, I., Bamba, S., Cissé, M., Zida, A., Bamogo, R., Sirima, C., Yaméogo, B. K., Sanou, R., Drabo, F., Dabiré, R. K., & Guiguemdé, R. T. (2015). Prevalence of intestinal opportunistic parasites infections in the University hospital of Bobo-Dioulasso, Burkina Faso. Infectious Diseases of Poverty **4(**1), 1–6. (https://doi.org/10.1186/s40249-015-0065-x)

Sayyari, A. A., Aminzade. F., Bagheri Yazdi, S. A., Karami, H, & Yaghoobi, M. (2005). Prevalence of intestinal parasitic infections in the Islamic Republic of Iran. *Eastern mediteranian health journal* **11**(3) 377-383.

Scanlan, P. D. (2012). Blastocystis: past pitfalls and future perspectives. *Trends in Parasitology* **28**(8), 327–334.doi:10.1016/j.pt.2012.05.001.

Schmidt, W., Wahnschaffe, U., Schäfer, M., Zippel, T, and, M., Meyerhans, A., and Ullrich, R. (2001). The rapid increase of mucosal CD4 T cells followed by clearance of intestinal cryptosporidiosis in an AIDS patient receiving highly active antiretroviral therapy. *Gastroenterology* **120**(4), 984-987.

Shadduck, J. A., & Pathobiology, V. (2019). Human Microsporidiosis and AIDS Author (s): John A. Shadduck Published by Oxford University Press Stable.

Shenoy, N., Ramapuram, J. T., Shenoy, A., Ahmed, J., & Srikant, N. (2017). Incidence of Opportunistic Infections among HIV-Positive Adults on Highly Active Antiretroviral Therapy in a Teaching Hospital, India: Prospective Study. *Journal of the International Association of Providers of AIDS Care* (JIAPAC), **16**(3), 309–311.doi: 10.1177/2325957416686192.

Shenoy, N., Ramapuram, J. T., Shenoy, A., Ahmed, J., & Srikant, N. (2017). Incidence of Opportunistic Infections among HIV-Positive Adults on Highly Active Antiretroviral Therapy in a Teaching Hospital, India: Prospective Study. Journal of the International Association of Providers of AIDS Care **16**(3), 309–311. (https://doi.org/10.1177/2325957416686192)

Shey Nsagha, D., Anna Njunda, L., Jules Clement Assob, N., Wenze Ayima, C., Asangbeng Tanue, E., Dzemo Kibu, O., & Emmanuel Kwenti, T. (2017). Prevalence and Predisposing Factors to Intestinal Parasitic Infections in HIV/AIDS Patients in Fako Division of Cameroon. American Journal of Epidemiology and Infectious Disease **5**(3), 42–49. (https://doi.org/10.12691/ajeid-5-3-1)

Shields, J. M., & Olson, B. H. (2003). *Cyclospora cayetanensis :* a review of an emerging parasitic coccidian **33**, 371–391. https://doi.org/10.1016/S0020-7519(02)00268-0.

Shimelis Assefa., Berhanu Erko., Girmay Medhin., Zelalem Assefa & Shimelis Techalew. (2009). Intestinal parasitic Infections concerning HIV/AIDS status, diarrhea, and CD4 T-cell count. *Biomedical Center infectious diseases* **9**(1): 155. (https://doi.org/10.1186/1471-2334-9-155)

Siberry, G. K., Abzug, M. J., Nachman, S., Brady, M. T., Dominguez, K. L., Handelsman, E., Mofenson, L. M., & Nesheim, S. (2013). Guidelines for the Prevention and Treatment of Opportunistic Infections in HIV-Exposed and HIV-Infected Children. The Pediatric Infectious Disease Journal **32**(2**)**.

Sim, L. H., & Lim, A. L. (2011). How common is intestinal parasitism in HIV-infected

patients in Malaysia ? **28**(2), 400–410.

Sissay Menkir, & Hiwtie Mengestie. (2014). Prevalence of intestinal parasitic infections among people with and without HIV infection and their association with diarrhea in Debre Markos Town, East Gojjam Zone, Ethiopia (Doctoral dissertation, Haramaya University).

Soave, R. (2020). *Cyclospora :* An Overview Author (s): Rosemery soave published by oxford university press stable URL :https:// www.jstor.org/stable/4459650 Cyclospora : An Overview **23**(3), 429–435.

Stenzel, D. J., & Boreham, P. F. L. (1996). Blastocystis hominis revisited. *Clinical Microbiology Reviews* **9**(4), 563–584. (https://doi.org/10.1128/cmr.9.4.563)

Tadel Girum., Abebaw Wasie., & Abdulsmed Worku. (2020). Trend of HIV / AIDS for the last 26 years and predicting achievement of the 90 – 90-90 HIV prevention targets by 2020 in Ethiopia : a time series analysis. 2018, 1–10.

Tan, K.S., (2008). New insights on classification, identification, and clinical relevance of *Blastocystis* species. *Clinical Microbiology Reviews* **21**(4), pp.639-665.

Tan, T. C., & Suresh, K. G. (2006). Amoeboid form of Blastocystis hominis a detailed ultrastructural insight. 737–742. (https://doi.org/10.1007/s00436-006-0214-z)

Taye, B., Desta, K., Ejigu, S., & Dori, G. U. (2014). The magnitude and risk factors of intestinal parasitic infection in relation to Human Immunodeficiency Virus infection and immune status, at ALERT Hospital, Addis Ababa, Ethiopia. Parasitology International, **63**(3), 550–556. (https://doi.org/10.1016/j.parint.2014.02.002)

Techalew Shimelis, Yayehyirad Tassachew & Tariku Lambiyo. (2016). *Cryptosporidium* and other intestinal parasitic infections among HIV patients in southern Ethiopia: the significance of improved HIV-related care. *Parasites Vectors* **9**, 270. (https://doi.org/10.1186/s13071-016-1554-x)

Tegegne Eshetu., Getinet Sibhatu., Mohammed Megiso., Abrham Abere., Habtamu Wondifraw Baynes., Belete Biadgo and Ayalew Jejaw Zeleke. (2017). Intestinal parasitosis and their associated factors among people living with HIV at the University of Gondar Hospital, Northwest-Ethiopia. *Ethiopian Journal of health sciences* **27**(4): 411-420.

Teklay Gebrecherkos., Haftom Kebede &Abebaw Addis Gelagay. (2019). Intestinal parasites among HIV/AIDS patients attending University of Gondar Hospital, northwest Ethiopia. *Ethiopian Journal of Health Development* **33**(2).

Termmathurapoj, S., Leelayoova, S., Aimpun, P., Thathaisong, U., Nimmanon, T., Taamasri, P., & Mungthin, M. (2004). The usefulness of short-term in vitro cultivation for the detection and molecular study of Blastocystis hominis in stool specimens. Parasitology Research **93**(6), 445–447. (https://doi.org/10.1007/s00436-004-1157-x)

Thom, Kerri & Forrest, Graeme. (2006). Gastrointestinal infections in immunocompromised hosts. *Current opinion in gastroenterology*. 22. 18-23. 10.1097/00001574-200401000-00005. African journal of science and research **4**(5), 13-17.

Tigist Gerzmu., Elsabet Fantahun., Esteselam Hailu., Hamelmal Kibe., Ousman Fesseha., Wanzahun Godana, &Woiynshet G/Tsadik. (2015). Prevalence of intestinal parasitosis among HIV/AIDS patients attending ART clinic of Arbaminch hospital African journal of science and research **4**(5), 13-17.

Tuom Gebrewahid., Gebretsadkan Gebrekirstos., Mebrahtu Teweldemedhin., Hailay Gebreyesus., Abrham Awala, & Kiros Tadla. (2019). Intestinal parasitosis in relation to CD4 count and anemia among ART initiated patients in St. Mary Aksum general hospital, Tigray, Ethiopia. *Biomedical Center Infectious Diseases*, **19**(1), 1–9.

UNAIDS. (2019). AIDSinfo.unaids.org

Varatharajalu, R., & Kakuturu, R. (2016). Strongyloides stercoralis: current perspectives. Reports in Parasitology 5, 23. (https://doi.org/10.2147/RIP.S75839)

Velasco-Hernandez, J. X., Gershengorn, H. B., & Blower, S. M. (2002). Could widespread use of combination antiretroviral therapy eradicate HIV epidemics? *The Lancet infectious diseases* **2**(8), 487-493.

Walther, Z., & Topazian, M. D. (2009). Isospora cholangiopathy: case study with histologic characterization and molecular confirmation. Human Pathology **40**(9), 1342–1346. (https://doi.org/10.1016/j.humpath.2009.01.020)

Wang, Z., Liu, Q., Liu, H., Li, S., Zhang, L., Zhao, Y., & Zhu, X. (2018). Prevalence of *Cryptosporidium, microsporidia* and *Isospora* infection in HIV-infected people: a global systematic review and meta-analysis. 1-19. (https://doi.org/10.1186/s13071-017-2558-x)

Wanyiri, J. W., Kanyi, H., Maina, S., Wang, D. E., Steen, A., Ngugi, P., Kamau, T., Waithera, T., Connor, R. O., Gachuhi, K., Wamae, C. N., Mwamburi, M., & Ward, H. D. (2014). Cryptosporidiosis in HIV / AIDS Patients in Kenya: Clinical Features, Epidemiology, Molecular Characterization and Antibody Responses 91(2), 319–328. (https://doi.org/10.4269/ajtmh.13-0254)

Wawrzyniak, I., Poirier, P., Viscogliosi, E., Dionigia, M., Texier, C., Delbac, F. , & Alaoui,

H.E. (2013). *Blastocystis*, an unrecognized parasite: An overview of pathogenesis and diagnosis. *Therapy Adv. Infect. Dis* **1**(5): 167-178.

White, A. C., Chappell, C. L., Hayat, C. S., Kimball, K. T., Flanigan, T. P., & Goodgame, R. W. (1994). Paromomycin for Cryptosporidiosis in AIDS: A Prospective, Double-Blind Trial.419–424.

WHO, http://www.who.int/mediacentre/factsheets/fs3 60/en/. HIV/AIDS Fact sheet Updated November 2020.

Wiwanitkit, V. (2001). Intestinal parasitic infections in Thai HIV-infected patients with different immunity status. *Biomedical Center Gastroenterology*, 1, 3–5. Retrieved from https://doi.org/10.1186/1471-230X-1-3.

World Health Organization.(1997). Basic laboratory methods in medical parasitology.WHO, Geneva.

World Health Organization. (2002), Author Provisional WHO clinical case study of Intestinal parasites in AIDS patients. Weekly Epidemiological Record (10):303–306.

World Health Organization. (2002). Social Mobilization and Training Control, Prevention and Eradication Department Communicable Diseases Cluster. Geneva.Switzerland. Pp 6-10.

World Health Organization. (2019). Bench aids of diagnosis of intestinal parasites, 2nd ed World Health Organization. (Http://apps.who.int/iris/handle/106665/324883. License: CC BY NC-SA 3.0 IGO)

World Health Organization/Department of Neglected Tropical Diseases. (2019). *Bench aids*.

Wurtz, R. (1994). Cyclospora: a newly identified intestinal pathogen of humans. *Clinical infectious diseases* **18**(4), 620-623.

Xiao, L., & U., M. Ryan. (2008). Molecular epidemiology. In: *Cryptosporidium* and Cryptosporidiosis, (Fayer, R. and Xiao, L., 2nd eds). CRC Press and IWA Publishing, USA, pp. 387-410.

Yeshanew Solomon., Tadesse Teshome. (2017). Prevalence of intestinal parasites among HIV seropositive individuals at Mettu Karl Hospital, Southwest Ethiopia (preliminary study). *Inernationa Journal of Health Scecei Research* **7**(2):275-280. *A Clinical Reevaluation*. (2007). December 2013.

Yonatan kindie and Shiferaw Bekel. (2016). Prevalence and Risk Factors for Intestinal Parasite Infections in HIV/AIDS Patients with Anti-Retroviral Treatment in South West Ethiopia. *Journal of Tropical Disease* **4**: 210.doi:10.4172/2329-891X.1000210.

Zelalem Tekel-Mariam., Abebe, G., & Mulu, A. (2008). Opportunistic and other intestinal parasitic infections in AIDS patients, HIV seropositive healthy carriers and HIV seronegative individuals in southwest Ethiopia. *East African Journal of Public Health* **5**(3), 169–173. (https://doi.org/10.4314/eajph.v5i3.38998).

Zelalem Teklemariam., Degu Abate., Habtamu Mitiku., & Yadeta Dessie. (2013). Prevalence of Intestinal Parasitic Infection among HIV Positive Persons Who Are Naive and on Antiretroviral Treatment in Hiwot Fana Specialized University Hospital, Eastern Ethiopia. Isrn Aids, 2013, 1–6.
(https://doi.org/10.1155/2013/324329).

Zeynudin Ahmed, K., Hemalatha, S., Kannan. (2013). Prevalence of opportunistic intestineal parasitic infection among HIV infected patients who are taking antiretroviral treatmentat Jimma Health Center, Jimma, Ethiopia. *European Revolution Medicine Pharmacology Science* **17** (4): 513-516.

9 APPENDICES

Appendix A: The prevalence of opportunistic intestinal protozoan parasites in Ethiopia.

		Prevalence OIPP in percent (%)						
**Study district**	**Sample size**	***Cryptosporidium spp***	***Cystoispora belli***	***Cyclospora cayetanensis***	***Blastocyts***	**Factors Identified**	**Reference**	**Methods of diagnosis**
South western Ethiopian	372	11	7.4`	3.7	X		Mohamed Awole *et al.*, 2003	A, C, D
St. Paul Hospitals in Addis Ababa.	330	28.6	22.5	-		X	Endeshaw Tokola, 2005	A, C, D, E, F
Lege Dini Eastern, Ethiopia	655	12.2	-	-		Drinking water from protected and unprotected sources	Dawit Ayalew, 2008	A, C, D
Nekemte Hospital, West Ethiopia	296	13.9	4.7	-		X	Mebrate Dufera *et al.*, 2008	D
Adama, Afar and Dire-Dawa	200	5	1.5	-	2.5	Lower $CD4^+$ T cell count (<200 cells/µL)	Haile-eyesus Adamu and Beyene Petros, 2009	A, C, D
Hawassa Teaching and Referral Hospital	214	20.1	12.2	-	0.9	$CD4^+$ T CELL	(Shimelis Assefa *et al.*, 2009)	A, C, D
Gondar, Northwest Ethiopia	284	8.4	-	-		X	(Nigus Telele, 2010)	A, C. D

Gambie Higher clinic Bahir Dar	248	43.6	15.3	-	10.5	X	Abebe Alemu *et al.*, 2011	A, B, C, D
Nekemte Hospital	259	25.1	-	-		$CD4^{+}$ cell count	(Anteneh Mulat, 2012)	A, C, D,
Jimma Health Center, Jimma, Ethiopia	397	13.3	10	-		The habit of handwashing before the meal, usages of latrine and duration treatment	Zeynudin Ahmed *et al.*, 2013	A,C, D
Northeast Ethiopia. Dessie Hospital	272	1.5	0.7	-		lower CD4 <200cells/cells/µl, Absence of toilet, s source of water, living condition, WHO stage and ART status	Assefa Missaye *et al.*, 2013	A, C, D
Addis Ababa Hospitals	222	8.1	2.3	-		X	Haileeyesus Adamu *et al.*, 2013	A.C.D
Jimma Hospital, Mother Theresa Missionary Charity Centre	160	15.4	3.9	-		X	(Teklemariam Zelalem *et al.*, 2013)	A, B, C, D
Hiwot Fana Specialized University Hospital	371	2.2	1.3	-		$CD4^{+}$ T CELL	(Zelalem Teklemariam *et al.*, 2013)	A, C, D
ALERT Hospital Addis Ababa	140	1.43	0.71			$CD4^{+}$ cell <350	(Birhualem Taye *et al.*, 2014)	A, C, D
Debre Markose Hospital	384	6.2	4.2	-	-	X	(Sissay Menkir & Hiwtie Mengestie, 2014)	A, C,D
Yirgalem Hospital	268	34.3	1.5	-	-	-	(Mekonen Girma *et al.*, 2014)	A, C, D
Othona Hospital, Sodo	422	14.2	8.5	2.8	2.6	X	Menbereleul Mathwose *et al.*, 2014	A, C,D
Yirgalem Hospital	139	9.4	5	-		contact with animals	(Alemsegede Mesay, 2015)	A, C, D
Felege Hiwot Referral Hospital, Bahir Dar	399	5.85	1.3	-		X	(Habtom Kiros *et al.*, 2015)	A, C, D

Hawassa University	491	13.2	2.2	0.4	-	$CD4^+$ T cell count < 200 cells/µl	Techalew Shimelis *et al.*, 2016	A, C, D
Jimma university specialize Hospital	120	5.0				$CD4^+$ cell counts less than 200 cells/µl,	Yonatan kindie and Shiferaw Bekel, 2016	A, C, D
Eastern Tigray	384	15.68	7.1	0.42		X	Dinku Senbeta *et al.*, 2017	A,C,D
Butajira	323	17.16	14.4			The presence of animals; using river water; undernutrition, and level of immunosuppression	Dereje Gedel *et al.*, 2017	A, C. D
Gondar Hospital	223	3.1	2.7	-		Absence of toilet improper handwashing before a meal and $CD4^+$ count < 200 cells/cells/µl	Tegegne Eshetu e*t al., 2017*	A, C, D
Dilchora Referral Hospital, Dire Dawa Town, Eastern	384	10.4	2.5	-		chewing Khat, eating unwashed and raw vegetables, contact with the animal, poor sanitation practice, low level of educational status and poor biomedical knowledge	(Abebech Yitagesu, 2018)	A, C, D
Komblecha	31	1.4	0.5	-	-	Utilization of water treating chemical rarely 0.4 (0.1–1.1)	(DanielGebretsadik *et al.*, 2018)	A, C, D
Arba Minch Hospital	220	8.63	1.36	5.90		Domestic animals and $CD4^+$ T-cell counts	(Gethanu Alemu *et al.*, 2018)	A, C, D
Wolyita Sodo Referral Hospital in South Ethiopia	384	9.6	-	-		$CD4^+$ Count Drinking spring water and animal contacts but not significant	(Care *et al.*, 2019)	A.C. D
Gondar Hospital, northwest Ethiopia	150	14.4	4	-	-	Have no toilet source of water from stream have diarrhea WHO stage III	(Teklay Gebrecherkos *et al.*, 2019)	A, C, D
St. Mary Aksum general hospital, Tigray	242	6.30	-	-	-	lack of handwashing before the meal, eating uncooked vegetables, history of taking anti-parasite medication, stool consistency, and anemia	(Tuom Gebrewahid *et al.*, 2019)	A, C, D

Note: A = Direct Wet Mount using Saline; B = Direct wet Mount with Saline and Iodine C = Formol-ether Sedimentation Method; D = Modified Ziel Neelson Staining E = ELISA Test; F = Autofluorescence; OIPP = Opportunistic Intestinal Protozoan Parasite, X = Not reported; "-" = Negative result

Appendix B: Questionnaire to be completed by Study Participants (English version)

Kindly fill the questionnaire taking into consideration that this data will employee only for scientific research. It is abiding by top confidentiality and privacy. Be informed that, it is your special response, which will help to conduct the study aimed to determine the prevalence and associated risk factors of OIPIs among HIV/AIDS patients attending ART at Debre Tabor General Hospital. The questionnaire is designed to find out the major risk factors that predispose HIV/AIDS patients to opportunistic intestinal parasitic infections attending at Debre Tabor General Hospital.

Direction- Circle your choice from the given alternatives below if the questions have options and write your immediate response to the space provided if there is no option.

N.B you can select/give more than one answers

Thank you in advance for your cooperation

Date DD_______ MM______ YY___________

Study code ------ Data collector name-----

Part one: Socio-demographic data

1. Sex___________
2. Age _______
3. Marital status
   A. Married B. Single C. Divorced D. Widowed
4. What is your educational status?
   A. No education B. No formal education but read and writes C. If you have formal educations write your specific grade level______ D. Diploma. Degree
5. What is your occupation _________________?
6. Residence A. Urban, B. Rural
7. Persons per household______________

Part two: Basic research questions

1. Source of drinking water mostly uses?
   A. Tap B. Well C. River/Stream D. Rain E. Borehole F. Spring
2. What type of water use for washing clothes, household activities and shower?
   A. Tap water B. River/steam C. Well water D. Spring, E. borehole
3. The habit of swimming in the river or other water bodies

A. Always B. Sometimes C Never

4. Toilet

A. Private water B. Public pit latrine D. Open field latrine

5. Handwashing habit after toilet

A. Always B. Sometimes C. Never

6. Handwashing habit before eating food

A. Always B. Sometimes C. Never

7. Are there animals around your residence? A. Yes B. No

8. Do you have close contact with an animal or their feces?

A. Yes B. No

9. Source of water for washing vegetables A. protected B. unprotected

10. Source of water for washing fruits A. protected B. unprotected C. No

11. When you travel to another town or place, do you consider the safety of the water that you drink? A Yes B. No

12. The habit of trimming fingernails A. Always B. Sometimes C. Never

13. Is there any special area in your village for the disposal of waste A.? Yes B. No—

14. How to dispose solid and liquid wastes____________

15. ART Started timeDD___MM_____YY______

16. Are you withdrawing ART by any means/factor A. Yes, B No

17. If your response option on question number "23" is 'A' how long withdraw it and by what reason? _____

18. Are you having diarrheal conditions for the last three months? A. Yes B. No

19. For how many weeks you have diarrhea

A. Less than two B. Between two and four C. Above four

Appendix C: Written Consent Form (English version)

Dear participants:

I am a student of master's in biomedical science at Bahir Dar University College of Science. I am going to conduct academic research in partial fulfillment of the requirement for the degree of Master of Science on the prevalence of opportunistic intestinal parasitic infections and its associated risk factors among HIV/AIDS patients at Debre Tabor General Hospital, Amhara, Ethiopia. Therefore, you are invited to be a participant of this study by giving a stool sample and an appropriate response to questionnaires on this issue. If you agree to participate in the study, please put your answers to questions giving to you. You have the right to refuse from participating in this study at any time, and refusal to participate will not result in the loss of any benefit you receive. Your information is kept by top confidentiality and privacy and collected in a unique code number.

If you understand the explanation, I request you to participate in the study.

Put your signature as illustrated below

Participant's response:

"I am clear about the study and agree to participate"

Participant's signature ________________date___________

Appendix D: Questionnaire (Amharic version)

ጥናታዊ ማጠይቅ

አባሪ ሀ፡ ጥናት ተሳታፊዎች የሚሞላ የጽሑፍ ማጠይቅ

የዚህ ማጠይቅ ዋና አላማበደብረ ታቦር ጠቅላላ ሆስፒታል የፀረ-የኤች አይ ቪ/ ኤድስ ህክምና እየተከታተሉ ያሉ ታካሚዎችን ለዕድለኛ የአንጀት ጥገኛ በሽታ አምጪ ተሕዋስያን እንዲጋለጡ/እንድያዙ ምቹ ሁኔታ የሚፈጥሩትን ዋና ዋና ምክንያቶች ላይ መረጃ ለማሰብሰብ ነው፡፡ ይህ ማጠየቅ ለሳይንሳዊ ምርምር ብቻ እንደሚሠራ ከግምት ወስጥ በማስገባት ማጠይቁን በቀና መንፈስ ይሙሉ፡፡ እርሰዎ የሚሰጡት መረጃ በከፍተኛ ሚስጥራዊነት እና ግላዊነት የሚጠበቅ ይሆናል።

አቅጣጫ ቀጥሎ የቀረቡትን ጥያቄዎች በማንበብ ጥያቄዉ ምርጫከሆነ የአርሰዎን ምላሽ የያዘዉን ፊደል ያክብቡ ክፍት ቦታ ከሆነ ደግሞ ሃሳበዎን ክፍት ቦታዉ ላይ ይፃፉ፡፡ ከአንድ በላይ ሚልሶችን መምረጥ / ማስጠት ይችላሉ፡፡

ስለ ትብብርዎ በቅድሚያ አማሰግናለሁ

ቀን-------ወር--------ዓመት-------የተሳትፊ ማለያ ኮድ------ የመረጃ ሰብሳቢ ስም-----

ክፍል አንድ: ማህበራዊና ሰነ ህዝባዊ መረጃዎች

1. ፆታ------
2. ዕድሜ---
3. የትዳር ሁኔታ ሀ. ያላገባ ለ. ያገባ ሐ. የፈታ መ. የሞተበት
4. የትምህርት ደረጃዎ ምንድነ ወ? ሀ. መደበኛ ያልሆነ ለ. ከተማሩ የክፍል ደረጀዉን ይፃፉ--- ------
   ሐ. ዲፕሎማመ. ዲግሪና በላይ
5. ሥራህ /ሽ ምንድን ነው___________________
6. መኖሪያ ሀ. ከተማ ለ. ገጠር
7. የቤተሰብ አባል ብዛት________

ክፍል ሁለት- ማሰረታዊ የምርምር ጥያቄዎች

1. አብዛኛወን ጊዜ ለማጠጥ የሚጠቀሙት የወሃ ምንጭከየቱ ነ ወ? ሀ. ከቧንቧለ. ከጉድጓድ ሐ. ከወንዝ / ጅረት መ. ከዝናብ ሠ. ከጥልቅ ጉድጓድ ረ. ከምንጭ
2. በወንዝ ወይምበሌሎች የወሃ አካላት ወስጥ የመዋኘት ልማድ ሀ. ሁል ጊዜ ለ. አንዳንድ ጊዜ ሐ. በጭራሽ
3. መጸዳጃ ቤት ሀ. የግል ለ. የህዝብ ጉድጓድ መጸዳጃ ቤት ሐ. ክፍት የማስክ መፀዳጃ
4. ከመፀዳጃ በኋላ የእጅ መታጠብ ልማድ ሀ. ሁል ጊዜ ለ. አንዳንድ ጊዜ ሐ. በጭራሽ
5. ምግብ ከመብላትዎ በፊት የእጅ መታጠብ ልማድ
   ሀ. ሁል ጊዜ ለ. አንዳንድ ጊዜ ሐ. በጭራሽ
6. በሚኖሩበት አካባቢ እንስሳት አሉ? ሀ. አዎ ለ. የለም
7. ከቤት እንስሳትወይምከፅዳጃቸዉጋር ንክኪ ያድርጋሉ?
   ሀ. አዎ ለ. የለም

8. አትክልትና ፍራፍርይ የመማ ብ ልማድ ሀ. ሁል ጊዜ ለ. አንዳንድ ጊዜ ሐ. በጭራሽ
9. ለምግብ የሚውሉ ቅጠላቅጠሎችን ለማጠብ የሚጠቀሙት የ ወሃ ምንጭሀ/የ ተጠበቀ ለ/ያልተጠበቀ
10. ለምግብ የሚውሉ ፍራፍርይ ለማጠብ የሚጠቀሙት የ ወሃ ምንጭሀ/የ ተጠበቀ ለ/ያልተጠበቀ ሐ. የ ለም
11. ወደ ሌላ ከተማ ወይም ቦታ ሲጓዙ፣ የሚጠጡትን የ ወሃ ደህንነ ት ከግምት ወስጥ ያስገ ባሉ?
ሀ. አዎን ለ. የ ለም
12. የ ጣት ጥፍሮችን የ መቁረጥ ልማድ ሀ. ሁልጊዜ ለ. አልፎአልፎ መ. በጭራሽ
13. ለቆሻሻ ማስወገ ጃ ተብሎ በሰፈራችሁ የ ተዘ ጋጀ ቦታ አለ ሀ. አወ ለ. የ ለም
14. ደረቅና ፈሳሽ ቆሻሻ በምን ሚልኩ ነ ውየ ምታስወግዱት----------------
15. የ ፀረ - ኤች አይ ቪ /ኤድስ መድሃ ኒት ማከታታለል የ ጀመሩበት ጊዜ
ቀን ____ወር ______ዓመት______
16. የ ፀረ -ኤች አይ/ቪ መድሃ ኒ ቱን መወሰድ ከጀመሩ በኋላ አቋርጠዉያዉቃሉ ሀ. አዎ ለ.የ ለም
17. በጥያቄ ቁጥር "21" ላይ ሚልሰዎ “ሀ” ከሆነ ለምን ያክል ጊዜ____
18. ከሶስት ወር ወዲህ ፈሳሽ የ አዘል ሰገ ራ በቀን ከሶስት ጊš በላይ ያስቅምጠዎት ነ በር? ሀ. አዎ ለ. የ ለም
19. በጥያቄ ቁጥር “23” ላይ ሚልሰዎ ሀ ከሆነ ምን ያክል ሳምንት ተቅማጡቆይቶብሃል/ሻል ሀ. ከሁለት በታች ለ. ከሁለት እስከ አራት ሐ. ከአራት በላይ

Appendix E: Consent Form (Amharic Version)

የ ፍቃድ ፎርም

የተሳታፊ መለያ ኮድ----------------

የተከበራችሁ ተሳታፊዎች

እኔ በባህር ዳር ዩኒቨርስቲ በሳይንስ ኮሌጅ የባዮሜዲካል ሳይንስ የማስተርስ ተማሪ ስሆን የሁለተኛ ዲግሪ ትምህርቴን ለማጠናቀቅ ጥናታዊ ፁሑፍ በማስራት ላይ እገኛለሁ፡፡ ጥናቱም የሚካሄደው በአማራ ብሔራዊ ክልላዊ መንግስት በደብረታቦር ጠቅላላ ሆስፒታል የፀረ-ኤችአይቪ/ኤድስ ህክምናን የሚከታተሉ ታካሚዎች ላይ ሲሆን ዓላማውም አጋጣሚ የአንጀት ጥገኛ ኢንፌክሽን ማስፋፋትንና ለዚህ በሽታ የሚያጋላጡ ተጓዳኝ ምክንያቶች ለማወቅና ለማየት ነው፡፡

ስለዚህ የእረሰዎ በፍቃደኝነት የሰገራ ናሙና ማስጠት እና ከዚህ ጉዳይ ጋር ለተያያዙ ማጢይቆች ተገቢውን ምላሽ ማስጠት ለጥናቱ ማሳካት ጉልህ አስተዋፅዖ ይኖረዋል፡፡ የእርሰዎ ማንኛውም ዓይነት መረጃ ለዚህ ጥናታዊ ምርምር ካልሆነ በስተቀር ለሌላ ጉዳይ ስለማይወል እርሰዎ ከዚህ ስጋት ነፃ ሁነዉበራስ በመተማመን ግልፅ የሆነ መረጃ ይሰጡኝ ዘንድ በትህትና እጠይቃለሁ፡፡ በዚህ ጥናት ወስጥ በማንኛውም ጊዜ ከመሳተፍ እምቢ የማለት መብት አልዎት፡፡ ለመሳተፍ እምቢ በማለትዎ የሚያገኙትን ማንኛውንም ጥቅም አያጡም፡፡ እርሰዎ የሚሰጡት መረጃ በከፍተኛ ሚስጥራዊነት እና ግላዊነት የሚጠበቅ ይሆናል።ከዚህ በተጨማሪ የእርስው መረጃ የሚሰበሰበዉበልዩ ኮድ ቁጥር ይሆናል።

የተሳታፊ ምላሽ-----------------------------

ስለ ጥናቱ ግልጽ ስለሆንኩ ለመሳተፍ እስማማለሁ”

የተሳታፊ ፊርማ___________________ ቀን________

Appendix F: Ethical clearance

ሳይንስ ኮሌጅ
የድህረ ምረቃ፤ምርምርና ማህበረሰብ
አገልግሎት ም/ዲን
ባሕር ዳር ዩኒቨርሲቲ
ባሕር ዳር - ኢትዮጵያ

Wisdom at the source of the Blue Nile

Science College
The Graduate, Research
& Community Services V/Dean
Bahir Dar University
Bahir Dar – Ethiopia

✉ 79
251 (582) 226 6597
ፋክስ Fax: 251 (582) 220- 20- 25
e-mail: negatassie@yahoo.com
website: www.bdu.edu.et

ቁ. ቁጥር: PGRCSVD/106/2012

ቀን: 11/ 07/2012 ዓ.ም.

Ethical Clearance Approval Form

Applicant's Name: Yitbarek Mulie

Research Title	Prevalence and associated risk factors of opportunistic intestinal parasitic infections among HIV/AIDS patients attending antiretroviral therapy in Debre Tabor General Hospital , Amhara, Ethiopia
Researcher (s) Name (s)	Yitbarek Mulie

Thank you for submitting your application for ethical clearance, which was considered at the College of Science Research Ethics Committee meeting on 20 March 2020. The committee has reviewed your ethical application, issues pertaining to participants, consent form, debriefing, and relevant questionnaires.

The researcher should keep the confidentiality of the identity of research participants and data that will be obtained from them. Any serious adverse events or significant changes which occur in connection with this study and /or which may alter its ethical consideration must be reported immediately to the committee for a possible ethical amendment.

We are therefore pleased to inform you that the College's Ethical Clearance Committee has approved your study from an ethical point of view.

With kind regards

CC//

- Dean office
- The Graduate, Research and Community Services V/Dean
- Biology Department
- **College of Science**
